# Mitochondrial complex I promotes kidney cancer metastasis

**DOI:** 10.1038/s41586-024-07812-3

**Published:** 2024-08-14

**Authors:** Divya Bezwada, Luigi Perelli, Nicholas P. Lesner, Ling Cai, Bailey Brooks, Zheng Wu, Hieu S. Vu, Varun Sondhi, Daniel L. Cassidy, Stacy Kasitinon, Sherwin Kelekar, Feng Cai, Arin B. Aurora, McKenzie Patrick, Ashley Leach, Rashed Ghandour, Yuanyuan Zhang, Duyen Do, Phyllis McDaniel, Jessica Sudderth, Dennis Dumesnil, Sara House, Tracy Rosales, Alan M. Poole, Yair Lotan, Solomon Woldu, Aditya Bagrodia, Xiaosong Meng, Jeffrey A. Cadeddu, Prashant Mishra, Javier Garcia-Bermudez, Ivan Pedrosa, Payal Kapur, Kevin D. Courtney, Craig R. Malloy, Giannicola Genovese, Vitaly Margulis, Ralph J. DeBerardinis

**Affiliations:** 1 Children’s Medical Center Research Institute, University of Texas Southwestern Medical Center, Dallas, TX USA; 2 Department of Genitourinary Medical Oncology, University of Texas MD Anderson Cancer Center, Houston, TX USA; 3 Quantitative Biomedical Research Center, University of Texas Southwestern Medical Center, Dallas, TX USA; 4 Department of Urology, University of Texas Southwestern Medical Center, Dallas, TX USA; 5 Department of Pediatrics, University of Texas Southwestern Medical Center, Dallas, TX USA; 6 Department of Radiology, University of Texas Southwestern Medical Center, Dallas, TX USA; 7 Kidney Cancer Program, University of Texas Southwestern Medical Center, Dallas, TX USA; 8 Department of Pathology, University of Texas Southwestern Medical Center, Dallas, TX USA; 9 Department of Internal Medicine, University of Texas Southwestern Medical Center, Dallas, TX USA; 10 Advanced Imaging Research Center, University of Texas Southwestern Medical Center, Dallas, TX USA; 11 Howard Hughes Medical Institute, University of Texas Southwestern Medical Center, Dallas, TX USA

**Keywords:** Cancer metabolism, Renal cell carcinoma, Metastasis

## Abstract

Most kidney cancers are metabolically dysfunctional^1–4^, but how this dysfunction affects cancer progression in humans is unknown. We infused ^13^C-labelled nutrients in over 80 patients with kidney cancer during surgical tumour resection. Labelling from [U-^13^C]glucose varies across subtypes, indicating that the kidney environment alone cannot account for all tumour metabolic reprogramming. Compared with the adjacent kidney, clear cell renal cell carcinomas (ccRCCs) display suppressed labelling of tricarboxylic acid (TCA) cycle intermediates in vivo and in ex vivo organotypic cultures, indicating that suppressed labelling is tissue intrinsic. [1,2-^13^C]acetate and [U-^13^C]glutamine infusions in patients, coupled with measurements of respiration in isolated human kidney and tumour mitochondria, reveal lower electron transport chain activity in ccRCCs that contributes to decreased oxidative and enhanced reductive TCA cycle labelling. However, ccRCC metastases unexpectedly have enhanced TCA cycle labelling compared with that of primary ccRCCs, indicating a divergent metabolic program during metastasis in patients. In mice, stimulating respiration or NADH recycling in kidney cancer cells is sufficient to promote metastasis, whereas inhibiting electron transport chain complex I decreases metastasis. These findings in humans and mice indicate that metabolic properties and liabilities evolve during kidney cancer progression, and that mitochondrial function is limiting for metastasis but not growth at the original site.

## Abstract (web summary)

Studying changes in the metabolic properties of kidney cancer in patients reveals an increased need for mitochondrial metabolism as tumors metastasize from the kidney to distant organs.

## Main

Mitochondrial alterations are a common feature of renal cell carcinomas (RCCs), but the mechanisms underlying mitochondrial anomalies vary among RCC subtypes. In clear cell RCC (ccRCC), the most common form of RCC, approximately 90% of tumours have biallelic inactivation of the gene encoding the tumour suppressor von Hippel–Lindau (*VHL*). Loss of VHL leads to pseudohypoxic stabilization of HIFα subunits and chronic activation of HIF target genes^5,6^, many of which promote glycolysis and suppress glucose oxidation^7,8^. A subset of chromophobe RCCs contain mutations in complex I of the electron transport chain (ETC)^2^, and oncocytomas accumulate defective mitochondria through somatic mutations in complex I and impaired mitochondrial elimination programs^9,10^. Pathogenic defects in the metabolic enzymes fumarate hydratase (FH) and succinate dehydrogenase (SDH) are also initiating events in some renal cancers^11,12^. These data imply that many RCCs select for reduced mitochondrial metabolism during their initiation and growth in the kidney.

Despite genetic evidence for mitochondrial dysfunction, how these mutations affect nutrient metabolism in human RCCs in vivo is unknown. Intraoperative infusion of ^13^C-labelled nutrients and analysis of ^13^C labelling in metabolites extracted from surgically resected samples can reveal metabolic differences between tumours and adjacent tissue or among tumours from different patients^13^. We previously reported suppressed contribution of glucose carbon to TCA cycle intermediates in five human ccRCCs^1^, implying reduced glucose oxidation in these tumours. Here we studied why this phenotype occurs, whether it generally characterizes primary kidney tumours and how metabolic properties evolve during ccRCC progression to metastatic disease.

## Kidney cancers use glucose variably

**Fig. 1:**
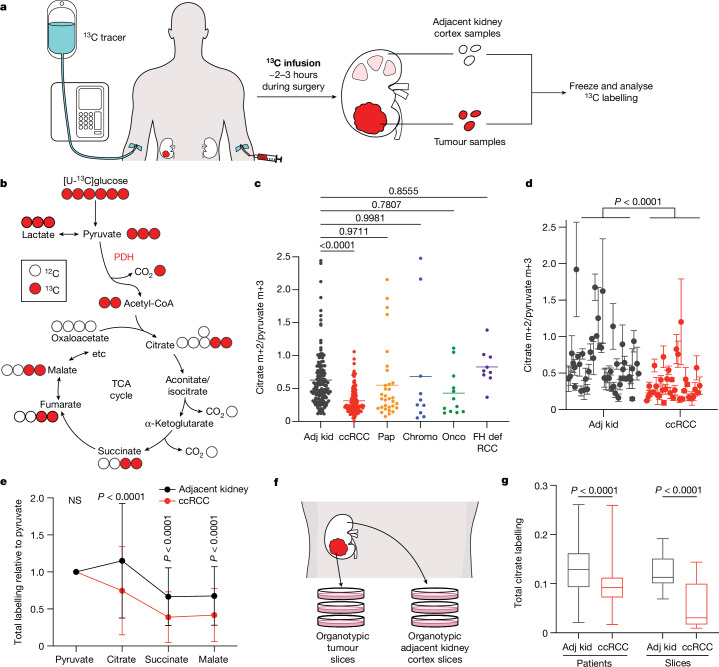
Glucose metabolism varies among kidney cancer subtypes. **a**, Schematic of intraoperative infusions. **b**, Isotopologue labelling in the TCA cycle from [U-^13^C]glucose via PDH. **c**, Citrate m+2/pyruvate m+3 ratio from [U-^13^C]glucose-infused patients. Each point reflects one tissue fragment (138 adjacent kidney (Adj kid) fragments from 48 patients, 109 ccRCC fragments from 37 patients, 32 papillary RCC (Pap) tissue fragments from 11 patients, 10 chromophobe RCC (Chromo) fragments from 4 patients, 12 oncocytoma (Onco) fragments from 4 patients, and 9 FH-deficient RCC (FH def RCC) fragments from 3 patients). **d**, Nested analysis of citrate m+2/pyruvate m+3 ratios separated by patient. Each data point represents a different patient. Error bars reflect the standard deviation from three fragments, tissue permitting, from the same patient. *n* = 48 patients for adjacent kidney and 37 patients for ccRCC. **e**, Total isotopologue labelling (1 − [m+0]) of TCA cycle intermediates divided by total isotopologue labelling of pyruvate. *n* = 48 patients for adjacent kidney and 37 patients for ccRCC. **f**, Schematic of organotypic cultures. Approximately 300-μm tissue sections were cultured on polytetrafluoroethylene inserts in 5% O_2_. **g**, Total citrate labelling (1 − [m+0]) from [U-^13^C]glucose in patients or tissue slices after 3 h of labelling. *n* = 48 patients for adjacent kidney, 37 patients for ccRCC, and 6 patients for adjacent kidney and ccRCC slices. All data represent mean ± s.d. Whiskers of box plots indicate minimum and maximum values. Statistical significance was assessed using a one-way analysis of variance (ANOVA) with a multiple comparison adjustment using Tukey’s methods (**c**), nested two-sided *t*-test (**d**) or unpaired two-sided *t*-tests with a Benjamini–Hochberg’s multiple comparison adjustment (**e**,**g**). Significant *P* values are indicated on figure panels. NS, not significant (*P* > 0.05). Source Data

**Extended Data Fig. 1:**
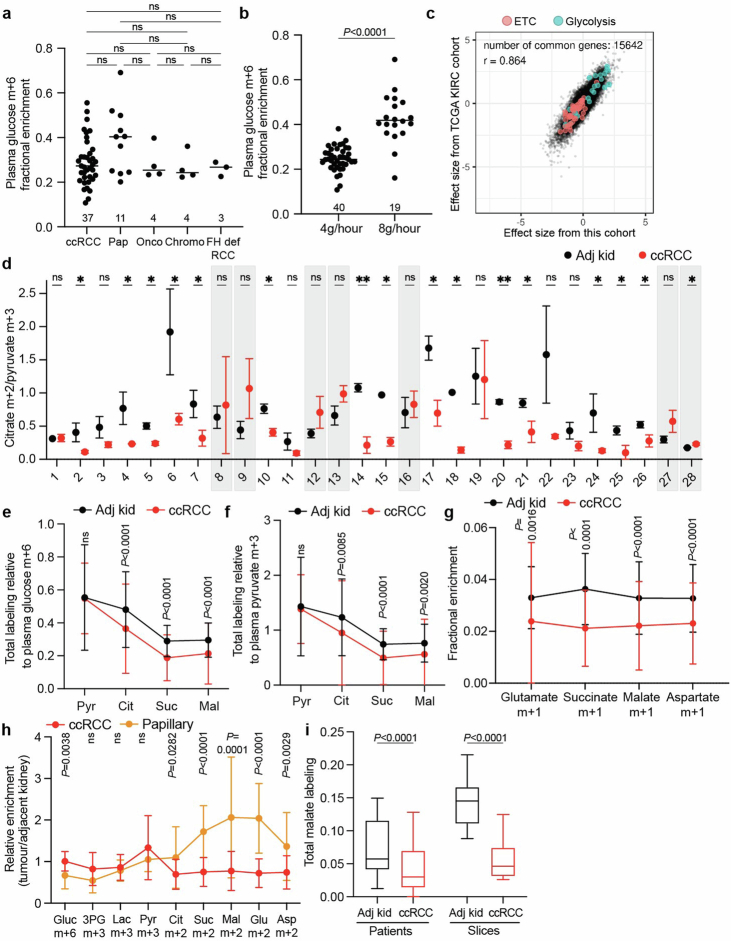
Clinical cohort reflects heterogenous ccRCC biology. Plasma m+6 glucose enrichment from patients infused with [U-^13^C]glucose separated by **(a)** kidney cancer subtype or **(b)** continuous infusion rate. The number of patients per group is indicated on the graph. **(c)** Correlation of RNA sequencing data from the TCGA KIRC cohort reporting ccRCC tumours versus ccRCC tumours infused with [U-^13^C]glucose reported in this study. Data are plotted as the effect size (Cohen’s d) reflecting the increase (d > 0) or decrease (d < 0) in mRNA abundance in tumours relative to adjacent kidney. Genes involved in glycolysis and the electron transport chain (ETC) are highlighted. **(d)** Matched citrate m+2/pyruvate m+3 ratio from patients infused with [U-^13^C]glucose. The x-axis indicates 28 patients in whom both tumour and kidney tissue were available. Patients in whom the average citrate m+2/pyruvate m+3 ratio was higher in ccRCC tissue are highlighted in grey boxes; this difference reached statistical significance only in patient 28. **(e)** Total labeling relative to plasma glucose m+6 and **(f)** plasma pyruvate m+3 for TCA cycle metabolites. (48 patients for adjacent kidney, 37 patients for ccRCC.) **(g)** Fractional enrichment in m+1 TCA cycle isotopologues. (48 patients for adjacent kidney, 37 patients for ccRCC.) **(h)** Enrichment in glycolytic and TCA cycle intermediates associated with glucose oxidation for ccRCC and papillary tumours. Labelling is normalized to the matched adjacent kidney (28 ccRCC patients, 9 papillary RCC patients). **(i)** Total malate labeling (1-[m+0]) from [U-^13^C]glucose in patients or tissue slices after 3 h of labeling (48 patients for adjacent kidney, 37 patients for ccRCC, 6 patients for adjacent kidney and ccRCC slices). All data represent mean ± standard deviation. Statistical significance was assessed using one way analysis of variance (ANOVA) with a multiple comparison adjustment using Tukey’s methods, (**a**) or two-sided unpaired t-tests (**b, d-i**). A Benjamini-Hochberg’s multiple comparison adjustment was made for **d-i**. Significant *P* values are indicated or ns = not significant (*P* > 0.05) on panels (**a, b, e-i**). ns = not significant (*P* > 0.05), *P < 0.05, **P < 0.01. Adj Kid = adjacent kidney, ccRCC = clear cell renal cell carcinoma, Pap = papillary renal cell carcinoma, Chromo = chromophone renal cell carcinoma, Onco = oncocytoma, FH def. RCC = FH deficient renal cell carcinoma. Gluc = glucose, 3PG = 3-phosphoglycerate, Lac = lactate, Pyr = pyruvate, Cit = citrate, Suc = succinate, Mal = malate, Glu = glutamate, Asp = aspartate. Source Data

**Extended Data Fig. 2:**
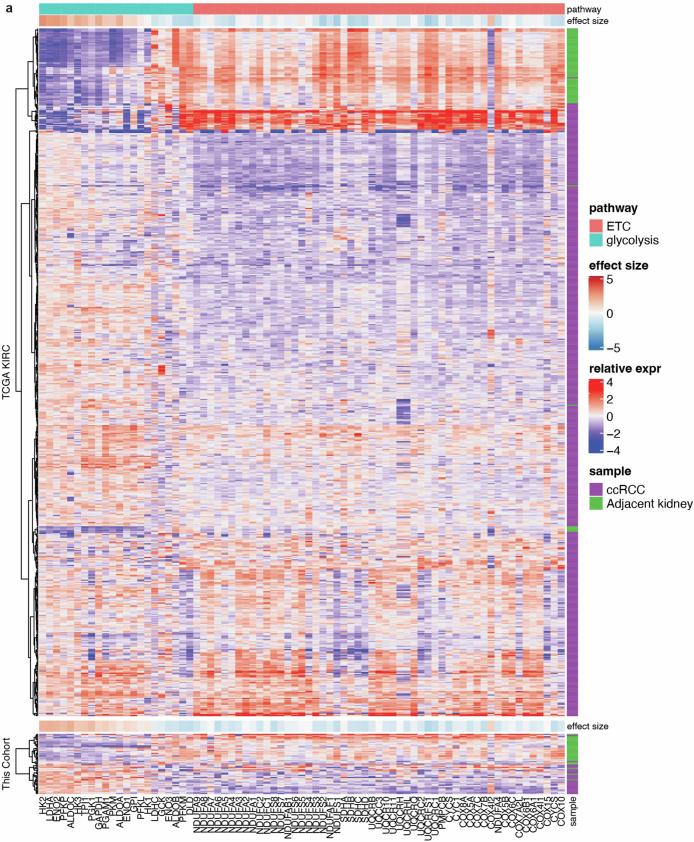
mRNA abundance of ETC and glycolysis genes in primary ccRCC tumours. **(a)** mRNA abundance for genes related to glycolysis and the electron transport chain (ETC) in the TCGA KIRC cohort and the cohort infused with [U-^13^C]glucose in this study. The ETC genes were selected from the gene ontology cellular component (cc) library combining complex I-IV genes. The glycolysis genes are shared genes among the following four gene sets: KEGG_GLYCOLYSIS_GLUCONEOGENESIS, REACTOME_GLYCOLYSIS, HALLMARK_GLYCOLYSIS, WP_GLYCOLYSIS_AND_GLUCONEOGENESIS. Source Data

**Extended Data Fig. 3:**
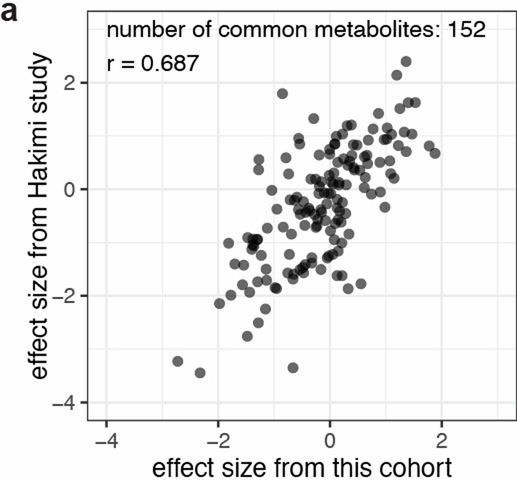
Consistency of metabolomics data between this cohort and a published cohort from a different center. **(a)** Correlation of metabolomics data from a published cohort with metabolomics from this study. Data are plotted as the effect size (Cohen’s d) reflecting the increase (d > 0) or decrease (d < 0) in metabolite abundance in ccRCC tumours relative to the adjacent kidney. Source Data

Patients undergoing partial or radical nephrectomy for kidney cancer were administered a ^13^C-labelled nutrient through a peripheral intravenous line during surgery (Fig. 1a). After resection (typically 2–3 h after the beginning of the infusion), tissue samples for metabolic analysis were chosen in consultation with the attending pathologist or pathology assistant. Using this approach, we studied 59 patients infused with [U-^13^C]glucose with various RCC subtypes, including 37 patients with ccRCC (clinical features of patients with primary RCC are in Supplementary Table 1). At the time of resection, plasma glucose labelling varied with the infusion rate (4 g h^−1^ versus 8 g h^−1^) but was unrelated to the cancer subtype (Extended Data Fig. 1a,b). ccRCCs infused with [U-^13^C]glucose exhibited high transcriptional correlation (*R* = 0.864) with The Cancer Gene Atlas (TCGA) Kidney Renal Clear Cell Carcinoma (KIRC) dataset (Extended Data Figs. 1c and 2 and Supplementary Table 2), and similar metabolomics features with tumours studied at another centre^14^ (Extended Data Fig. 3 and Supplementary Table 3). Therefore, the tumours we analysed with ^13^C infusions share molecular and metabolic characteristics with ccRCCs reported elsewhere.

The labelling ratio of citrate m+2 (that is, the fraction of citrate molecules containing two ^13^C nuclei) to pyruvate m+3 was lower in ccRCC samples than in adjacent kidney, indicating a reduced contribution of glucose through the pyruvate dehydrogenase (PDH) reaction (Fig. 1b,c; isotopologues are in Supplementary Table 4). Labelling was variable in the tumours and renal cortex (hereafter, adjacent kidney), reflecting both interpatient variability and regional labelling differences among samples from the same patient (Fig. 1d). When citrate m+2/pyruvate m+3 ratios were compared between ccRCC and adjacent kidney in the same patient, 17 patients had a statistically significant decrease in the tumour, and only 1 patient had a statistically significant increase (Extended Data Fig. 1d). Nine patients did not have matched adjacent kidney available for analysis. In addition to suppressed citrate m+2/pyruvate m+3 labelling ratios, total labelling of citrate and other TCA cycle intermediates (1 − [m+0]) was also suppressed in ccRCCs (Fig. 1e); this metric incorporates all routes of label entry into the TCA cycle and multiple turns of the cycle. Normalizing total labelling to glucose m+6 or pyruvate m+3 in the plasma produced similar results (Extended Data Fig. 1e,f), and m+1 intermediates produced from multiple TCA cycle turns were reduced in ccRCC tumours compared with the adjacent kidney (Extended Data Fig. 1g). Suppressed labelling of TCA cycle intermediates was not observed in all RCC subtypes (Fig. 1c and Extended Data Fig. 1h). We also generated viable agarose-embedded slices of kidney or ccRCC tissues from six patients and labelled them with [U-^13^C]glucose ex vivo in medium formulated to contain a nutrient content similar to human plasma^15^ (Fig. 1f). This revealed a similar degree of labelling suppression in citrate and malate as what was observed in the patients (Fig. 1g and Extended Data Fig. 1i). Together, the data indicate that low TCA cycle labelling from [U-^13^C]glucose is a specific, intrinsic property of ccRCC tumours that does not directly result from residence in the kidney and is not an artefact of surgery.

## Acetate and glutamine feed the TCA cycle

**Fig. 2:**
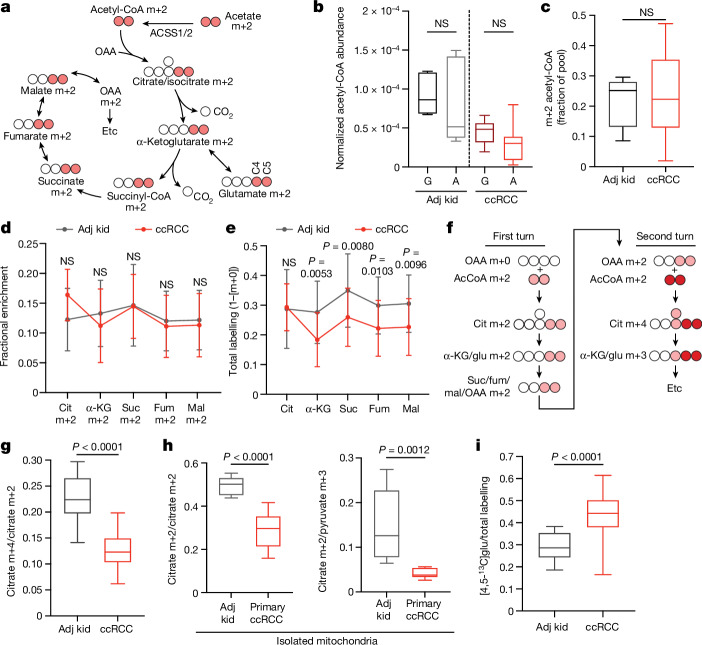
TCA cycle metabolism is suppressed in ccRCCs. **a**, Isotopologue labelling from [1,2-^13^C]acetate. ACSS1/2, acetyl-CoA synthetase 1 or 2; OAA, oxaloacetate. **b**, Total ion count-normalized acetyl-CoA abundance after infusion with [U-^13^C]glucose (G) or [1,2-^13^C]acetate (A). For [U-^13^C]glucose infusions, *n* = 6 adjacent kidney fragments from 2 patients and 10 ccRCC fragments from 4 patients. For [1,2-^13^C]acetate infusions, *n* = 15 adjacent kidney fragments from 5 patients and 32 ccRCC fragments from 12 patients. **c**, Enrichment of m+2 acetyl-CoA in adjacent kidney and ccRCC. *n* = 15 adjacent kidney fragments from 5 patients and 31 ccRCC fragments from 12 patients. **d**, m+2 isotopologues of TCA cycle intermediates from patients infused with [1,2-^13^C]acetate. Cit, citrate; fum, fumarate; mal, malate; suc, succinate. **e**, Total labelling (1 − [m+0]) of TCA cycle intermediates from [1,2-^13^C]acetate-infused patients. *n* = 15 adjacent kidney fragments from 5 patients and 33 ccRCC fragments from 12 patients (**d**,**e**). **f**, ^13^C labelling from [1,2-^13^C]acetyl-CoA through two TCA cycle turns. AcCoA, acetyl-CoA; glu, glutamate. **g**, Citrate m+4/citrate m+2 ratios from adjacent kidney and ccRCC. *n* = 15 adjacent kidney fragments from 5 patients and 33 ccRCC fragments from 12 patients. **h**, Citrate m+4/citrate m+2 and citrate m+2/pyruvate m+3 ratios from mitochondria isolated from adjacent kidney or ccRCC. *n* = 9 fragments from 3 patients for both adjacent kidney and ccRCC. **i**, [4,5-^13^C]glutamate ([4,5-^13^C]glu) labelling as a fraction of total glutamate labelling after infusion with [1,2-^13^C]acetate. *n* = 18 adjacent kidney fragments from 5 patients and 81 ccRCC fragments from 12 patients. All data represent mean ± s.d. Whiskers of box plots are minimum and maximum values. Statistical significance was assessed using unpaired two-sided parametric *t*-tests (**b**–**e**,**g**–**i**). A Benjamini–Hochberg’s multiple comparison adjustment was made in panels **d**,**e**. Significant *P* values are indicated on the figure panels. Source Data

We next infused 12 patients with ccRCC with [1,2-^13^C]acetate (m+2), which can be converted to acetyl-CoA m+2 by acetyl-CoA synthetases (Fig. 2a). Unlike pyruvate, which can enter the TCA cycle through both acetyl-CoA and oxaloacetate to produce complex labelling on even the first TCA cycle turn, acetate only enters through acetyl-CoA to exclusively produce m+2 labelling in the first turn. [1,2-^13^C]acetate also contributes ^13^C to the TCA cycle independently of PDH, so it provides an approach to test TCA cycle labelling perturbations beyond PDH suppression. The conditions that we used to infuse [1,2-^13^C]acetate did not alter acetyl-CoA levels in tumours or adjacent kidneys and produced similar levels of acetyl-CoA labelling in both tissues (Fig. 2b,c and see Supplementary Table 5 for full isotopologue distributions). Fractional enrichments of m+2 TCA cycle intermediates in ccRCC tumours were also similar to adjacent kidney (Fig. 2d), indicating similar contributions to the TCA cycle under these conditions. However, total labelling (1 − [m+0]) of these metabolites revealed decreased labelling in tumours compared with in the kidney, consistent with reduced labelling beyond turn 1 of the TCA cycle (Fig. 2e).

**Extended Data Fig. 4:**
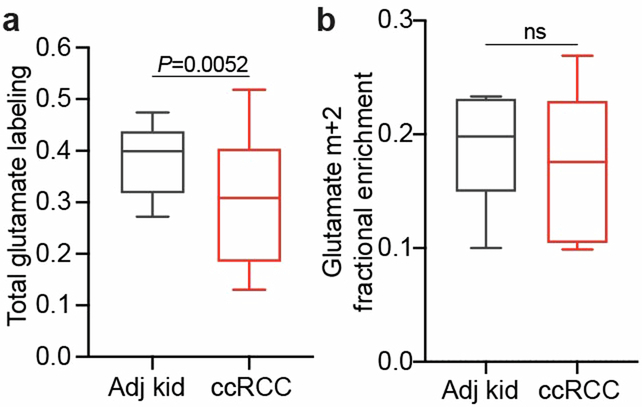
Glutamate enrichment in [1,2-^13^C]acetate-infused patients. **(a)** Total labeling (1-[m+0]) of glutamate from ccRCC patients infused with [1,2-^13^C]acetate (18 fragments from 5 patients for adjacent kidney, 81 fragments from 12 ccRCC patients). **(b)** Fractional enrichment of glutamate m+2 from ccRCC patients infused with [1,2-^13^C]acetate (15 fragments from 5 patients for adjacent kidney, 32 fragments from 12 ccRCC patients). All panels show mean ± standard deviations. Statistical significance was assessed using two-sided unpaired t-tests (**a,b**). Significant *P* values are indicated on figure panels. ns = not significant (*P* > 0.05). Adj Kid = adjacent kidney, ccRCC = clear cell renal cell carcinoma. Source Data

We then examined TCA cycle turnover in three ways. First, the high enrichment in acetyl-CoA (average of 20–25%) allowed us to observe higher-order labelling in TCA cycle intermediates from subsequent rounds of incorporation of acetyl-CoA m+2 (Fig. 2f). The ratio of citrate m+4 to citrate m+2, a marker of ^13^C retention through two cycles, was reduced by about 50% in tumours relative to kidneys (Fig. 2g). Second, we examined TCA cycle labelling in fresh mitochondria isolated from these resected tissues and cultured with [U-^13^C]pyruvate. Both the citrate m+2/pyruvate m+3 ratio and the citrate m+4/citrate m+2 ratio were decreased in ccRCC mitochondria compared with those in kidney mitochondria (Fig. 2h), supporting the hypothesis that TCA cycle labelling reflects mitochondrial perturbations beyond simple PDH suppression. Third, we examined positional ^13^C labelling in glutamate, which exchanges with α-ketoglutarate (α-KG) and is classically used as a reporter of TCA cycle metabolism^16^ (Fig. 2a). Total glutamate labelling was lower in tumours than in adjacent kidney (Extended Data Fig. 4a), but glutamate m+2 was similar (Extended Data Fig. 4b), mirroring the pattern in TCA cycle intermediates. Despite the similar m+2 fractional enrichment, the labelled ^13^C carbons had differential positioning in glutamate extracted from ccRCC tumours compared with the adjacent kidney. Using a mass spectrometry method that reports isotopic position with high sensitivity^17^, we determined that [4,5-^13^C]glutamate, which appears in the first turn of the cycle (Fig. 2a), accounts for a much higher fraction of glutamate labelling in tumours than in adjacent kidney (Fig. 2i). Therefore, most glutamate m+2 comes from the first turn of the TCA cycle, whereas labelling patterns requiring multiple turns are suppressed in ccRCC tumours.

**Fig. 3:**
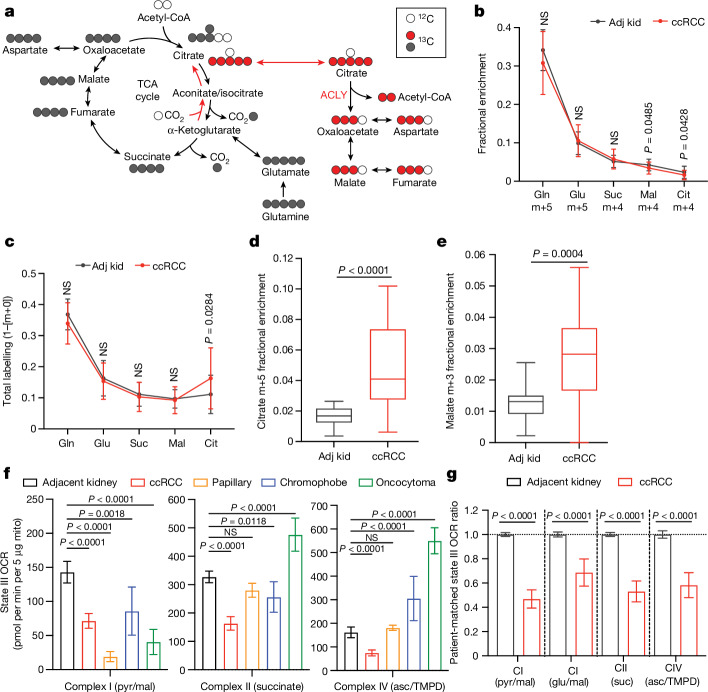
ccRCC mitochondria have low oxidative metabolism. **a**, Isotopologue labelling from [U-^13^C]glutamine. Labelling from oxidative metabolism is indicated in grey, whereas reductive metabolism is indicated in red. **b**, TCA cycle intermediate isotopologues from [U-^13^C]glutamine metabolism through the first oxidative TCA cycle turn. Gln, glutamine. **c**, Total labelling (1 − [m+0]) of TCA cycle intermediates from patients with ccRCC infused with [U-^13^C]glutamine. **d**, Fractional enrichment of m+5 citrate. **e**, Fractional enrichment of m+3 malate. *n* = 21 fragments from 7 patients for adjacent kidney and 54 fragments from 7 patients for ccRCC (**b**–**e**). **f**, State III ADP-stimulated OCRs from mitochondria isolated from primary human tissues. Substrates used to stimulate respiration are indicated in parentheses. *n* = 92 replicates from 21 patients for adjacent kidney, 74 replicates from 21 patients for ccRCC, 24 replicates from 3 patients for chromophobe RCC, 16 replicates from 3 patients for oncocytoma and 16 replicates from 3 patients for papillary RCC. Asc, ascorbate; pyr, pyruvate; TMPD, *N*,*N*,*N*,*N*-tetramethyl-*p*-phenylenediamine. **g**, OCRs from ccRCC mitochondria normalized to the patient-matched adjacent kidney. *n* = 54 replicates from 12 patients for adjacent kidney and 54 replicates from 12 patients for ccRCC. Statistical significance was assessed using unpaired two-sided parametric *t*-tests (**b**–**e**,**g**), and a one-way ANOVA with a multiple comparison adjustment using Tukey’s methods (**f**). A Benjamini–Hochberg’s multiple comparison adjustment was made in panels **b**,**c**,**g**. Whiskers of box plots (**d**,**e**) represent minimum and maximum values. Error bars represent s.d. (**b**,**c**) and mean ± 95% CI (**f**,**g**). Significant *P* values are indicated on the figure panels. Source Data

**Extended Data Fig. 5:**
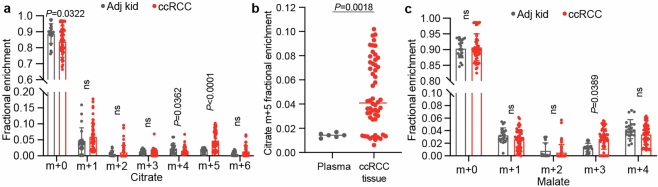
Metabolite labeling in plasma and tissues of patients infused with [U-^13^C]glutamine. **(a)** Citrate isotopologue distributions in [U-^13^C]glutamine-infused tissues (21 fragments from 7 patients for adjacent kidney, 54 fragments from 7 patients for ccRCC). **(b)** Fractional abundance of citrate m+5 in plasma at the time of resection and in ccRCC tumour tissue samples (Plasma samples from 7 patients, 54 ccRCC tissue samples from 7 patients). **(c)** Malate isotopologue distributions in [U-^13^C]glutamine-infused tissues (21 fragments from 7 patients for adjacent kidney, 54 fragments from 7 patients for ccRCC). All data reflect mean ± standard deviations. The mean is indicated in panel **b**. Statistical significance was assessed using unpaired t-tests with a Benjamini-Hochberg’s multiple comparison adjustment (**a-c**). Significant *P* values are indicated on figure panels. ns = not significant (*P* > 0.05). ccRCC = clear cell renal cell carcinoma. Source Data

To assess the TCA cycle using a third tracer, we infused seven patients with ccRCC with [U-^13^C]glutamine. Glutamine is the most abundant amino acid in the circulation, and its uptake in the tumour microenvironment is dominated by malignant cells^18^. Although glutamine can contribute to many pathways, we focus on its utilization as a TCA cycle substrate. The contribution of glutamine to the TCA cycle involves conversion to α-KG followed by either oxidation through α-KG dehydrogenase or reductive carboxylation by isocitrate dehydrogenase 1 or isocitrate dehydrogenase 2 (ref. ^19^). In cell culture, labelling through reductive metabolism is enhanced by processes that suppress pyruvate oxidation, including *VHL* loss, PDH suppression and mitochondrial defects^20,21^. Isotope labelling in citrate and other TCA cycle intermediates can discriminate pathway use (Fig. 3a). Patient infusions produced similar glutamine m+5 enrichment in tumours and adjacent kidneys (30–35%; Fig. 3b). Labelling of glutamate m+5 and TCA cycle intermediates from the first turn of the oxidative TCA cycle (m+4) were also similar between tumour and kidney (Fig. 3b), as was total labelling (1 − [m+0]) of these metabolites (Fig. 3c and see Supplementary Table 6 for all isotopologues). Citrate was an exception, with higher total labelling in tumours than in kidneys (Fig. 3c). The excess labelling in citrate involved enhanced contributions from the reductive pathway, as indicated by high citrate m+5 in most fragments (Fig. 3d and Extended Data Fig. 5a). This level of labelling exceeded labelling in plasma citrate, indicating that it resulted from metabolism localized to the tumour (Extended Data Fig. 5b). The tumours also contained relatively high levels of malate m+3, indicating further metabolism along the reductive pathway (Fig. 3e and Extended Data Fig. 5c). Therefore, glutamine is used as a nutrient in human ccRCC, and its metabolism results in oxidative and reductive labelling of TCA cycle intermediates.

## Most kidney cancers have low respiration

**Extended Data Fig. 6:**
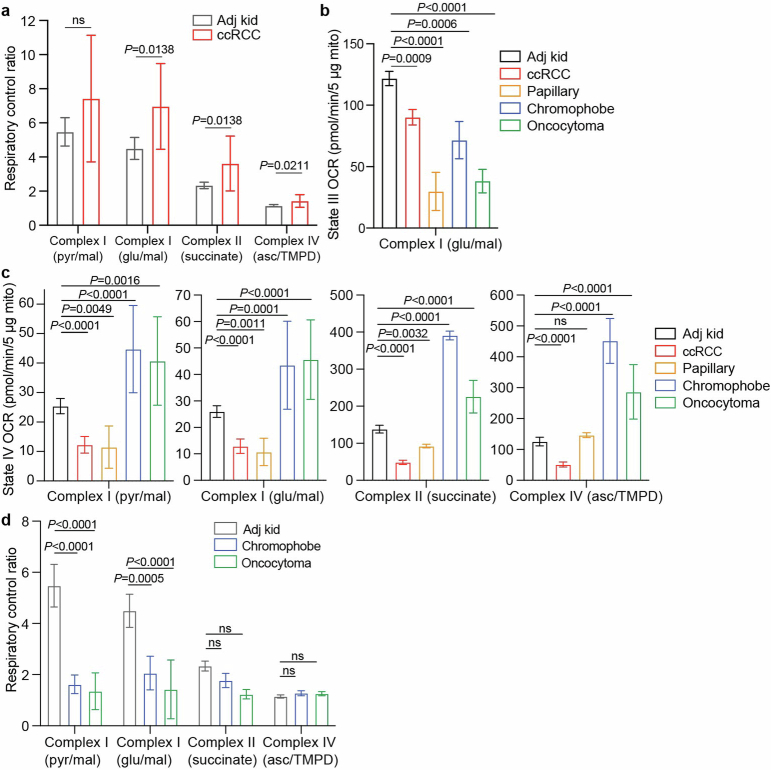
Respiration of mitochondria from primary human kidney cancers. **(a)** Respiratory control ratio (RCR) for mitochondria from the adjacent kidney and ccRCCs. RCR is the ratio of the State III ADP-stimulated oxygen consumption rate (OCR) to the State IV basal OCR. (49 adjacent kidney replicates from 12 patients, 17 ccRCC replicates from 5 patients). **(b)** State III ADP-stimulated OCR from mitochondria isolated from primary human tissues, using glutamate and malate to stimulate complex I. (91 replicates from 21 patients for adjacent kidney, 74 replicates from 21 patients for ccRCC, 21 replicates from 3 patients for chromophobe RCC, 16 replicates from 3 patients from oncocytoma, and 16 replicates from 3 patients for papillary RCC). **(c)** State IV basal OCR from mitochondria isolated from primary human tissues. Injected substrates are indicated under each complex. (92 replicates from 21 patients for adjacent kidney, 74 replicates from 21 patients for ccRCC, 21 replicates from 3 patients for chromophobe RCC, 16 replicates from 3 patients from oncocytoma, and 16 replicates from 3 patients for papillary RCC). **(d)** Respiratory control ratio (RCR) for chromophobe RCCs and oncocytomas (52 adjacent kidney samples from 12 patients, 16 oncocytoma samples from 3 patients, 16 chromophobe samples from 3 patients). Panels **a**-**c** show mean ± 95% confidence intervals, and panel **d** shows mean ± standard deviation. Statistical significance was assessed using an unpaired two-sided parametric t-test with a Benjamini-Hochberg’s multiple comparison adjustment (**a**) or one way analysis of variance (ANOVA) with a multiple comparison adjustment using Tukey’s methods (**b-d**). Significant *P* values are indicated on figure panels. ns = not significant (*P* > 0.05). pyr = pyruvate, mal = malate, glu = glutamate, asc = ascorbate, TMPD = N,N,N,N-tetramethyl-p-phenylenediamine. Source Data

We emphasize that some of the labelling features used above, including glutamate positional labelling, citrate m+2/pyruvate m+3 and citrate m+4/citrate m+2, reflect the ratio of TCA cycle turning to anaplerosis rather than the absolute TCA cycle turning rate. Reductions in these features in tumours could reflect higher anaplerosis or lower TCA cycle turning, so we sought to examine these possibilities. Suppressed labelling of TCA cycle intermediates from [U-^13^C]glucose and enhanced reductive labelling from [U-^13^C]glutamine can result from ETC dysfunction, which reduces TCA cycle turning^22^. Multiple groups have reported decreased mitochondrial DNA (mtDNA) content^23,24^ and reduced expression of ETC components in RCC^14,25^. However, these analyses did not assess coupled respiration in mitochondria from tumours and kidneys. We measured oxygen consumption rates (OCRs) of mitochondria immediately after harvest from fresh, surgically resected kidney and tumour tissues. We used a differential centrifugation protocol to isolate mitochondria and assess ADP-stimulated (state III) and ADP-unstimulated (state IV) respiration. Mitochondria from both the kidney and the ccRCC had normal respiratory control ratios (defined as state III/state IV respiration) when supplied with complex I substrates, indicating that the preparation produced mitochondria with the expected ability to stimulate respiration after the addition of ADP^26^ (Extended Data Fig. 6a). However, absolute state III and state IV OCRs were low in ccRCC compared with kidney mitochondria for complexes I, II and IV (Fig. 3f and Extended Data Fig. 6b,c). To account for day-to-day variability in measuring respiration, we also normalized OCR values from ccRCC mitochondria to patient-matched kidney mitochondria in 12 patients that provided both tissues. In these patients, the OCRs from complex I, II and IV were always lower in mitochondria isolated from tumours (Fig. 3g).

Mitochondria from other RCC subtypes displayed low state III respiration at complex I, but variable activities of other ETC components (Fig. 3f). Chromophobe tumours and oncocytomas contain mutations in genes encoding complex I subunits^2,27^, and, accordingly, both had low complex I activity relative to adjacent kidney (Fig. 3f). The respiratory control ratios of these mitochondria were also low when provided with complex I substrates (Extended Data Fig. 6d). However, absolute state III OCRs for complex II and complex IV were variable; in mitochondria from oncocytomas, they exceeded rates from kidney mitochondria. Therefore, oncocytomas and chromophobe tumours display the expected defects in complex I, with relative preservation of other ETC activity.

## Metastases increase TCA cycle labelling

**Fig. 4:**
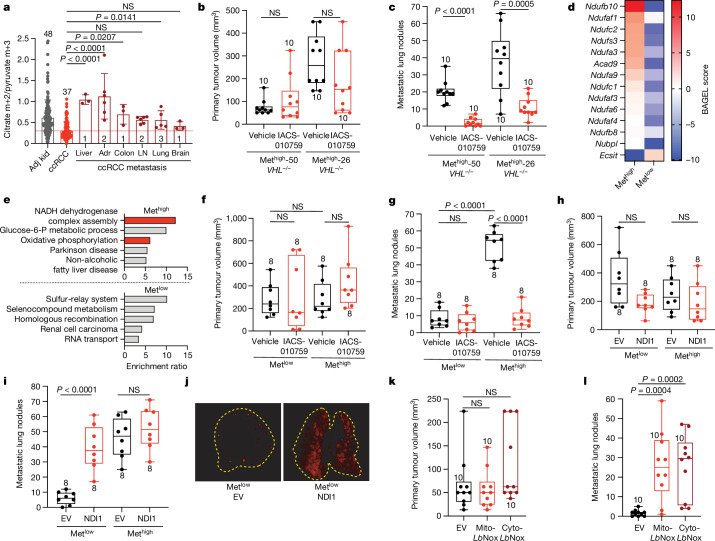
Complex I is required for efficient metastasis in mice. **a**, Citrate m+2/pyruvate m+3 ratio from patients infused with [U-^13^C]glucose. ccRCC metastases to different organs are in brown. For metastases, the number of patients is indicated on each bar and individual fragments are shown. Data for adjacent kidney and primary ccRCCs are from Fig. 1c. The red dotted line indicates the citrate m+2 to pyruvate m+3 mean for primary ccRCC. Adr, adrenal gland; LN, lymph node. **b**,**c**, Primary tumours (**b**) and metastatic lung nodules (**c**) 21 days after introduction of *VHL*^*−/−*^ cells into the renal capsule and treatment with vehicle or IACS-010759. **d**, BAGEL scores for complex I genes essential for either Met^high^ or Met^low^ proliferation. **e**, Gene ontology enrichment ratios for Met^high^ and Met^low^ cells; the red bars are related to the ETC. **f**,**g**, Primary tumours (**f**) and metastatic lung nodules (**g**) treated with vehicle or IACS-010759 for 21 days. **h**,**i**, Primary tumours (**h**) and metastatic lung nodules (**i**) from Met^high^ or Met^low^ cells expressing an empty vector (EV) or NDI1. **j**, Representative images of Met^low^ metastasis-bearing lungs expressing empty vector or NDI1. Original images are provided in Supplementary Data. **k**,**l**, Primary tumours (**k**) and metastatic lung nodules (**l**) from Met^high^ or Met^low^ cells expressing empty vector, mito-*Lb*NOX or cyto-*Lb*NOX. The number of mice per group is indicated on the figure panels (**b**,**c**,**f**–**i**,**k**–**l**). All data represent mean ± s.d. The whiskers of box plots represent minimum and maximum values. Statistical significance was assessed using a one-way ANOVA with a multiple comparison adjustment using Dunnett’s method (**a**) and unpaired two-sided parametric Student’s *t*-tests (**b**,**c**,**f**–**i**,**k**–**l**). Significant *P* values are indicated on the figure panels. Source Data

**Extended Data Fig. 7:**
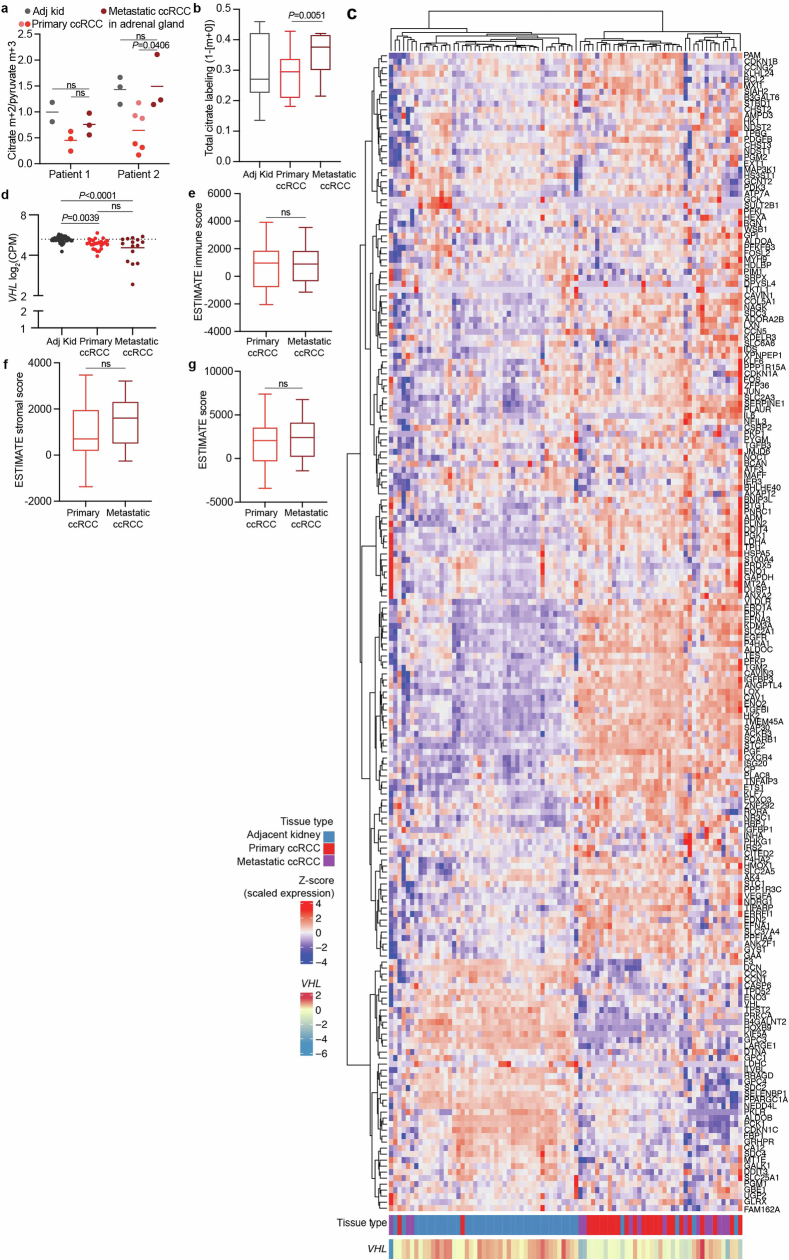
Metabolic and molecular characteristics of primary and metastatic ccRCCs. **(a)** Citrate m+2/pyruvate m+3 ratio from two patients infused with [U-^13^C]glucose who had a primary ccRCC and synchronous metastasis to the adrenal gland removed during the same infusion and surgery. **(b)** Total citrate labeling (1-[m+0]) from a patient infused with [1,2-^13^C]acetate who had ccRCC metastases at two organ sites. (15 fragments from 5 patients for adjacent kidney, 33 fragments from 12 patients for ccRCC, 6 fragments from two metastases from 1 patient). **(c)** Scaled expression of genes in the HALLMARK_HYPOXIA gene set from the adjacent kidney (44 patients), primary ccRCC (25 patients), and metastatic ccRCC tumours (15 patients). **(d)** Log_2_(CPM) expression of *VHL* in the adjacent kidney (44 patients), primary ccRCC (25 patients), and metastatic ccRCC tumours (15 patients). **(e)** Estimation of STromal and Immune cells in MAlignant Tumour tissues using Expression data (ESTIMATE) estimation of immune and **(f)** stromal infiltrate and **(g)** tumour cell purity (ESTIMATE score) in primary and metastatic ccRCC tumours from RNA sequencing data. (22 patients for primary tumours, 15 patients for metastatic tumours). Statistical significance was assessed with a one-way analysis of variance (ANOVA) with a multiple comparison adjustment using Tukey’s methods (**a, d**) or an unpaired two-sided parametric t-test (**b, e-g**). Significant *P* values are indicated on figure panels. ns = not significant (*P* > 0.05). Adj Kid = adjacent kidney, ccRCC = clear cell renal cell carcinoma, CPM = counts per million. Source Data

Patients with early-stage ccRCC have a 5-year survival rate close to 95%, whereas patients with distant metastatic ccRCC fare much worse, with 5-year survival rates under 15% (ref. ^28^). How emergent metabolic properties support metastasis is a subject of intense investigation^29–33^. Most human studies describing metabolic alterations during metastasis are based on transcriptional data rather than direct assessment of metabolism in tumours^34,35^. To directly examine metabolism in metastatic ccRCC, [U-^13^C]glucose was infused in 10 patients undergoing metastasectomy. Metastatic tumours had higher citrate m+2/pyruvate m+3 ratios than the average citrate m+2/pyruvate m+3 ratio from primary ccRCCs in the kidney (Fig. 4a). Two patients with both a primary ccRCC and an adrenal metastasis underwent concurrent nephrectomy and adrenalectomy, allowing both lesions to be sampled during the same infusion. Compared with the primary lesion, the metastatic adrenal tumours trended towards higher citrate m+2/pyruvate m+3 ratios (Extended Data Fig. 7a). In patient 2, two different regions of the primary tumour were sampled, with one region having a reduced citrate m+2/pyruvate m+3 ratio relative to the other; both of these regions had somewhat lower ratios than the metastasis (Extended Data Fig. 7a). One patient with two metastatic tumours was infused with [1,2-^13^C]acetate, and these tumours also displayed elevated citrate labelling compared with primary ccRCCs (Extended Data Fig. 7b). These data indicate that circulating fuels such as glucose and acetate generally make larger contributions to the TCA cycle in metastatic than in primary ccRCC. RNA sequencing revealed that primary and metastatic ccRCCs from our cohort expressed similar levels of *VHL* and genes related to hypoxia (Extended Data Fig. 7c,d). Gene expression also indicated that the primary and metastatic ccRCCs that we sampled contained similar abundances of stromal cells, immune cells and tumour cell purity (Extended Data Fig. 7e–g), making it unlikely that non-malignant cells in the tumour microenvironment are the predominant source of ^13^C labelling differences. The small size of these metastatic tumours precluded direct analysis of mitochondrial respiration.

## Efficient metastasis requires complex I

**Extended Data Fig. 8:**
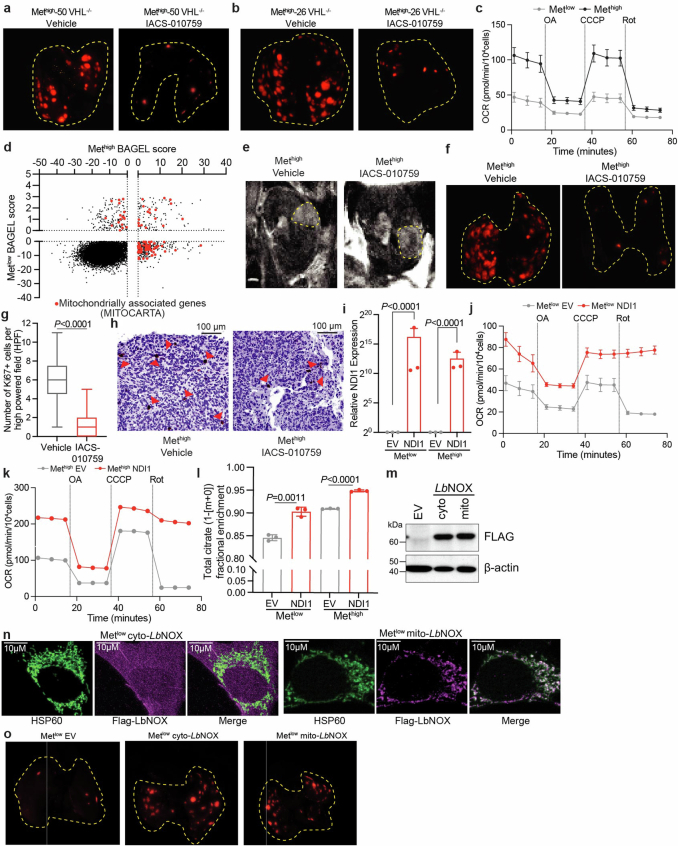
ETC complex I drives spontaneous metastasis of renal cell carcinoma in mice. **(a)** and **(b)** Representative images of metastasis-bearing lungs from vehicle and IACS-010759-treated mice. **(c)** Oxygen consumption rate (OCR) of Met^low^ and Met^high^ cells. 12 replicates per cell line. **(d)** BAGEL scores for Met^high^ and Met^low^ cell proliferation CRISPR screen. Mitochondrially associated genes are indicated in red (n = 102, 25 for Met^low^, 77 for Met^high^), and all other genes are indicated in black (n = 17,975). Genes selectively essential for Met^low^ proliferation are in the top left quadrant, and genes selectively essential for Met^high^ proliferation are in the bottom right quadrant. **(e)** Representative images of orthotopic Met^high^ tumour-bearing kidneys treated with vehicle or IACS-010759. **(f)** Representative images of Met^high^ metastasis-bearing lungs treated with vehicle or IACS-010759. **(g)** Number of cells with Ki67-positive nuclei per high powered field (HPF). Data are from 5 HPF per mouse (5 mice per group, total 25 quantified fields per group). **(h)** Representative Ki67 staining of Met^high^ lung metastases treated with vehicle or IACS-010759 from panel **g**. Red arrows indicate Ki67-positive nuclei. **(i)** RT-qPCR of *NDI1* expression in Met^low^ and Met^high^ cells. 3 replicates per group; control values were normalized to 1. **(j)** OCR of Met^low^ and **(k)** Met^high^ cells expressing EV or NDI1. 30 replicates for Met^high^ EV, 32 replicates for Met^high^ NDI1, 8 replicates for Met^low^ EV, 11 replicates for Met^low^ NDI1. **(l)** Total citrate labeling of Met^low^ and Met^high^ cells expressing EV or NDI1. 3 replicates per group. **(m)** Anti-FLAG western blot of Met^low^ cells expressing EV or FLAG-tagged cyto-*Lb*NOX or mito-*Lb*NOX. For gel source data, see Supplementary Fig. 1. Immunoblots were repeated two additional times with similar results. **(n)** Representative immunofluorescence imaging of HSP60 (for mitochondria), FLAG (for *Lb*NOX), and merged images of HSP60 and FLAG for Met^low^ cyto-*Lb*NOX and mito-*Lb*NOX cells. Representative images were taken from ten images that showed similar results. **(o)** Representative images of Met^low^ metastasis-bearing lungs expressing EV, cyto-*Lb*NOX, or mito-*Lb*NOX. Statistical significance was assessed using unpaired two-sided t-tests (**g, i, l**). Original images for are provided in Supplementary Data (**a**, **b**, **e**, **f**, **n**, **o**). Panels **c**, **j**, and **k**, show mean ± standard error of the mean (S.E.M.), and panels **h**, **i**, and **l** show mean ± standard deviations. Significant *P* values are indicated on figure panels. ns = not significant (*P* > 0.05). EV = empty vector, cyto = cytosol, mito = mitochondria, OA = oligomycin, CCCP = carbonyl cyanide 3-chlorophenylhydrazone, R = rotenone. Source Data

To examine whether oxidative mitochondrial metabolism supports metastasis, we used somatic mosaic genetically engineered mouse models of RCC capable of spontaneous metastasis from the kidney^36^. These models use kidney-specific inactivation of RCC tumour suppressor-encoding genes such as *Vhl* and *Setd2*, with biallelic deletion of a 40-kb region of mouse chromosome 4q, which is syntenic to a region of human chromosome 9p21 (*4q*^*9p21−*^) that is frequently lost in human metastatic kidney cancers. This combination of mutations results in tumours that arise in the kidney and spontaneously metastasize to the lung. Metastatic tumour burden is easily followed by TdTomato expression. Primary cell lines derived from these models can be genetically manipulated ex vivo and reimplanted into the kidney. We implanted two cell lines derived from *Vhl*^*KO*^*;Setd2*^*KO*^;*4q*^*9p21−*^ renal tumours into the kidneys of NU-*Foxn1*^*nu*^ mice, randomized the mice to receive vehicle or the ETC complex I inhibitor IACS-010759, and followed tumour burden in the kidney and lung. In the kidney, IACS-010759 had no effect on tumour growth (Fig. 4b), but metastasis was markedly suppressed in both lines (Fig. 4c and Extended Data Fig. 8a,b).

We next used a pair of lines derived from renal *Setd2*^*KO*^*;Nf2*^*KO*^*;4q*^*9p21−*^ tumours, one that metastasizes frequently from the kidney (Met^high^) and another that does not (Met^low^). Both of these mutations are observed in human ccRCC, although less frequently than *VHL* loss. The frequency of *SETD2* mutations increases in metastatic ccRCC^37,38^, and these models provided the appealing opportunity to study metabolic liabilities in the context of isogenic but metastatically divergent tumours. In culture, Met^high^ cells respired more rapidly than Met^low^ cells (Extended Data Fig. 8c). A genome-wide CRISPR screen identified disparate dependencies in Met^high^ and Met^low^ cells, with several gene sets related to mitochondria, particularly NADH dehydrogenase (ETC complex I) being among the most essential for Met^high^ cells in culture (Fig. 4d,e and Extended Data Fig. 8d). To assess the requirement for complex I in vivo, Met^high^ and Met^low^ cells were implanted into the kidney and treated with DMSO or IACS-010759 as described for the *Vhl*^*KO*^ models. Renal Met^high^ and Met^low^ tumours grew at the same rate, with or without IACS-010759 treatment (Fig. 4f and Extended Data Fig. 8e). However, IACS-010759 reduced metastasis of Met^high^ tumours to levels similar to Met^low^ tumours (Fig. 4g and Extended Data Fig. 8f). Residual metastasis of IACS-010759-treated Met^high^ cells resulted in small pulmonary tumours with low Ki67 staining (Extended Data Fig. 8g,h). These data indicate that RCC cells possessing the ability to metastasize need complex I to proliferate and form tumours in the lung, but tolerate inhibition of complex I in the kidney.

**Fig. 5:**
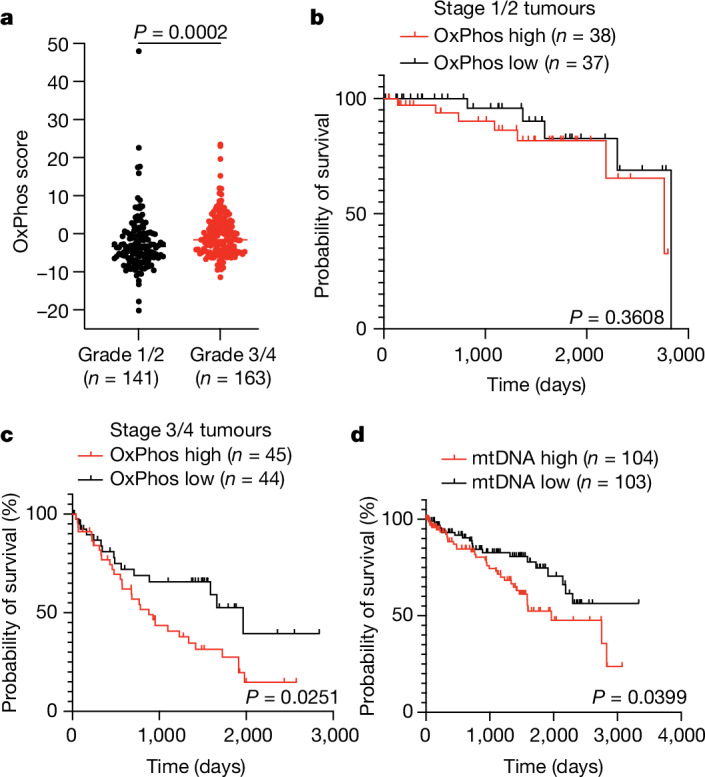
Elevated mitochondrial gene expression correlates with worse survival in human ccRCC. **a**, OxPhos scores for grade 1/2 tumours versus grade 3/4 tumours. Medians for each group are indicated by colour-coded dashed lines. OxPhos score calculations are described in the Methods. **b**,**c**, Kaplan–Meier survival curve of grade 3/4 tumours with level 1 or 2 staging (a primary tumour less than 7 cm confined to the kidney; **b**) or grade 3/4 tumours with level 3 or 4 staging (a primary tumour more than 7 cm or evidence of metastasis; **c**) classified by OxPhos score. **d**, Kaplan–Meier survival curve of patients from the TCGA KIRC dataset classified by mtDNA copy number (top 30% indicates mtDNA high; bottom 30% denotes mtDNA low). The number of patients in each group is indicated on the panels. Statistical significance was assessed using a two-sided Mann–Whitney rank test (**a**) and log-rank (Mantel–Cox) tests (**b**–**d**). *P* values are indicated on the panels. Source Data

The relatively low respiration of Met^low^ cells allowed us to ask whether activating the ETC confers the ability to metastasize. To address this question, we expressed the yeast mitochondrial NADH dehydrogenase NDI1 in both Met^high^ cells and Met^low^ cells (Extended Data Fig. 8i). NDI1 oxidizes NADH to NAD^+^ and transfers electrons to the coenzyme Q pool, essentially replacing the functions of mammalian complex I^39^. Cancer cells expressing NDI1 maintain TCA cycle labelling from [U-^13^C]glucose in vivo even when native complex I activity is impaired^22^. In cultured Met^low^ and Met^high^ cells, NDI1 enhanced respiration and increased labelling of TCA cycle intermediates from [U-^13^C]glucose (Extended Data Fig. 8j–l). In vivo, NDI1 had no effect on growth of either Met^high^ or Met^low^ tumours in the kidney (Fig. 4h) and did not increase metastatic burden of Met^high^ tumours (Fig. 4i). However, NDI1 was sufficient to drive metastasis of Met^low^ tumours to levels similar to Met^high^ tumours (Fig. 4i,j).

To delineate how complex I supports metastasis, we expressed an NADH oxidase from *Lactobacillus brevis* (*Lb*NOX) localized to either the mitochondria (mito-*Lb*NOX) or cytosol (cyto-*Lb*NOX)^40^ (Extended Data Fig. 8m,n). Like complex I, this water-forming enzyme regenerates NAD^+^ from NADH; however, unlike mammalian complex I and NDI1, the reducing equivalents are not used to reduce coenzyme Q. Therefore, *Lb*NOX separates the NAD^+^ regenerative function of complex I from its role in oxidative phosphorylation. Expression of *Lb*NOX in either compartment in Met^low^ orthotopic kidney tumours did not affect primary tumour growth (Fig. 4k), but increased metastatic lung tumour burden (Fig. 4l and Extended Data Fig. 8o) for both mito-*Lb*NOX and cyto-*Lb*NOX. These results imply that complex I supports RCC metastasis, at least in part, by providing a mechanism for NAD^+^ regeneration.

**Extended Data Fig. 9:**
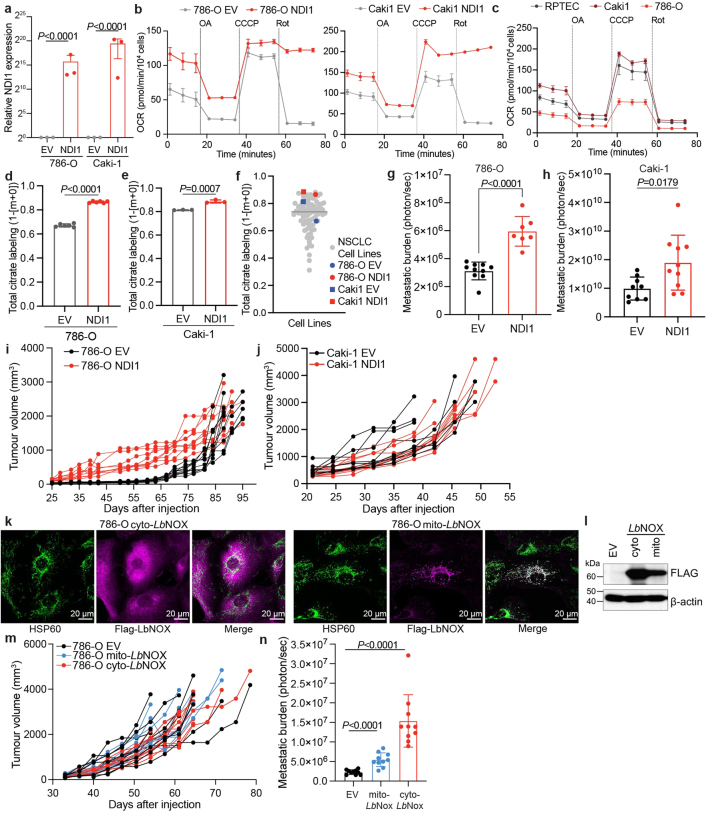
ETC complex I drives metastatic colonization of human ccRCC cell lines in mice. **(a)** RT-qPCR of *NDI1* expression in 786-O and Caki-1 cells. 3 replicates per group; control values were normalized to 1. **(b)** OCR of 786-O and Caki-1 cells expressing EV or NDI1. 6 replicates for 786-O EV and NDI1, 8 replicates for Caki-1 EV and NDI1. **(c)** OCR of renal proximal tubule epithelial cells (RPTEC) and 786-O and Caki-1 cells. 12 replicates for RPTEC, 8 replicates for 786-O, 8 replicates for Caki-1. **(d)** Total citrate labeling of 786-O or **(e)** Caki-1 cells expressing EV or NDI1. 6 replicates for 786-O cells expressing EV or NDI1, 3 replicates for Caki-1 expressing EV or NDI1. **(f)** Total citrate labeling (1-[m+0]) from cells cultured with [U-^13^C]glucose for 6 h in RPMI with 5% dialyzed FBS. Labelling from non-small cell lung cancer (NSCLC) cell lines was previously published^41^. 786-O data points reflect the average of 6 replicates and Caki-1 data points reflect the average of 3 replicates. **(g)** Metastatic burden in mice assessed with bioluminescence six weeks after tail vein injection of 786-O or **(h)** Caki-1 cells expressing EV or NDI1. 10 mice for 786-O EV, 7 mice for 786-O NDI1, 9 mice for Caki-1 EV, 10 mice for Caki-1 NDI1. Mice were excluded if signal was limited to the tail (3 mice for 786-O NDI1, 1 mouse for Caki1-EV). **(i)** Subcutaneous tumour volume of 786-O or **(j)** Caki-1 cells expressing EV or NDI1. 10 mice for 786-O EV, 9 mice for 786-O NDI1, 9 mice for Caki-1 EV, 9 mice for Caki-1 NDI1. Mice were excluded if euthanasia was required prior to endpoint or if mice died unexpectedly (1 per group for 786-O NDI1, Caki-1 EV, Caki-1 NDI1). **(k)** Representative immunofluorescence imaging of HSP60 (for mitochondria), FLAG (for *Lb*NOX), and merged images of HSP60 and FLAG for 786-O cyto-*Lb*NOX and mito-*Lb*NOX cells. Representative images were taken from ten images that showed similar results. Original images are in Supplementary Data. **(l)** Anti-FLAG western blot of 786-O cells expressing EV or FLAG-tagged cyto-*Lb*NOX or mito-*Lb*NOX. For gel source data, see Supplementary Fig. 1. Immunoblots were repeated two additional times with similar results. **(m)** Subcutaneous tumour volume of 786-O cells expressing EV, cyto-*Lb*NOX, or mito-*Lb*NOX. 10 mice per group. **(n)** Metastatic burden in mice assessed with bioluminescence after tail vein injection with 786-O cells expressing EV, cyto-*Lb*NOX, or mito-*Lb*NOX. 10 mice per group. Panels **a**, **d**, **e**, **g**, **h**, and **n** show mean ± standard deviations and panels **b** and **c** show mean ± standard error of the mean (S.E.M.). The NSCLC mean is indicated on panel **f**. Statistical significance was assessed using unpaired two-sided t-tests (**a, d, e, g, h, n**). Significant *P* values are indicated on figure panels. EV = empty vector, cyto = cytosol, mito = mitochondria, OA = oligomycin, CCCP = carbonyl cyanide 3-chlorophenylhydrazone, R = rotenone. Source Data

We performed analogous experiments in two human ccRCC cell lines (*VHL*-deficient 786-O and *VHL*-wild-type Caki1 cells) expressing dsRed-luciferase to allow imaging of metastatic colonization in the lung after injection of cells into the tail vein. NDI1 enhanced respiration and increased labelling of TCA cycle intermediates from [U-^13^C]glucose^41^ (Extended Data Fig. 9a–f). These effects were more prominent 786-O cells, which had lower respiration and TCA cycle labelling before NDI1 expression (Extended Data Fig. 9c–e). NDI1 expression did not enhance growth of subcutaneous tumours but increased lung metastases after tail-vein injection (Extended Data Fig. 9g–j). Similarly, *Lb*NOX did not increase subcutaneous growth of 786-O cells but increased metastatic burden (Extended Data Fig. 9k–n).

**Extended Data Fig. 10:**
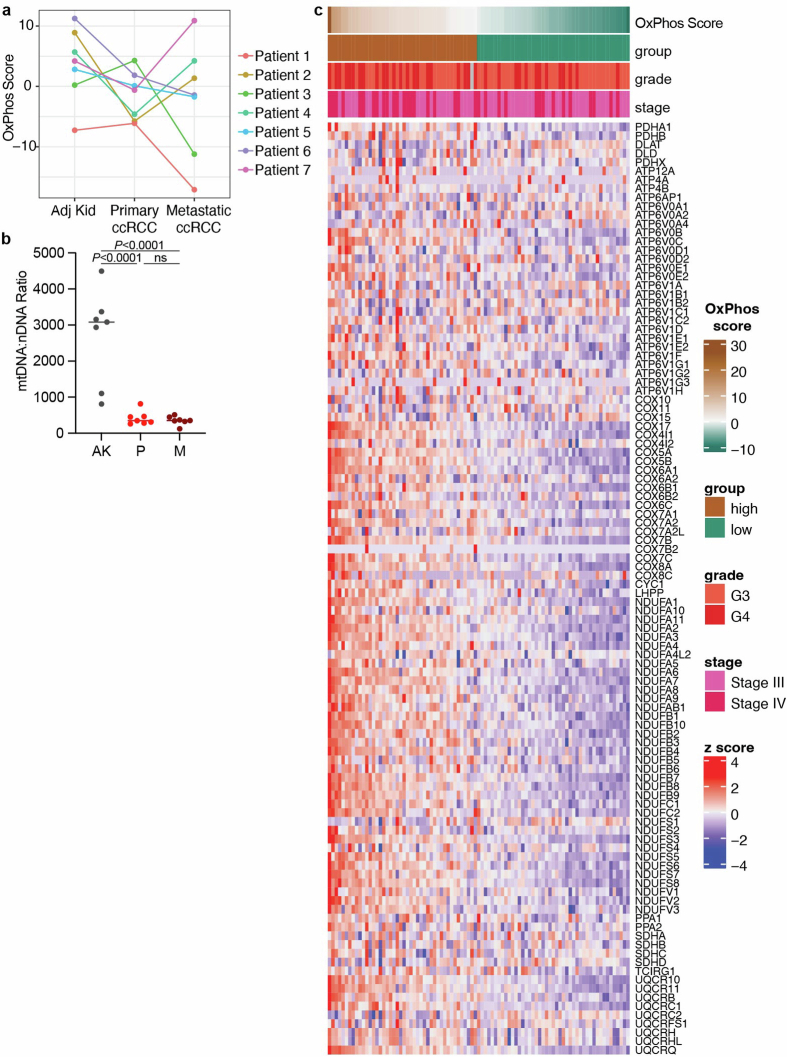
Increased mitochondrial gene signatures in primary human ccRCC are associated with worse survival. **(a)** OxPhos score from 7 matched ccRCC patients. OxPhos score calculations are described in the Methods. **(b)** mtDNA:nDNA ratio from adjacent kidney (AK), primary ccRCC (P), and metastastic ccRCC (M) from 7 patients. **(c)** Expression of genes in the OxPhos gene set used to calculate the OxPhos score. Samples are ordered according to the OxPhos score (highest to lowest). Statistical significance was assessed using one-way analysis of variance (ANOVA) with a multiple comparison adjustment using Tukey’s methods (**b**). Significant *P* values are indicated on figure panels. ns = not significant (*P* > 0.05). Adj kid = adjacent kidney, ccRCC = clear cell renal cell carcinoma. Source Data

Finally, we examined molecular features in human ccRCC that might explain the connection between mitochondrial metabolism and metastasis. Most reported ccRCC gene expression data are from primary tumours. We performed RNA sequencing from treatment-naive-matched kidney, primary ccRCC and metastatic ccRCC nodules from seven patients at UT Southwestern. Three of seven metastatic ccRCC nodules showed an increase in oxidative phosphorylation (OxPhos) gene expression relative to primary ccRCC, cumulatively shown as the OxPhos score (Extended Data Fig. 10a), but all metastases showed mtDNA depletion similar to the matched primary tumours (Extended Data Fig. 10b).

We reasoned that some molecular alterations observed in metastases may exist in high-grade primary tumours before metastasis. We analysed the TCGA ccRCC dataset (KIRC) for expression of OxPhos genes, collectively summarized as a singular OxPhos score. High grade 3 and 4 tumours had higher OxPhos scores than low grade 1 and 2 tumours (Fig. 5a). As outcomes in ccRCC are largely defined by metastasis, we reasoned that differences in OxPhos gene expression may portend shortened patient survival. We stratified high grade 3 and 4 tumours into two groups: stage 1/2 tumours (tumours less than 7 cm confined to the kidney) and stage 3/4 tumours (tumours more than 7 cm or tumours that have metastasized; Extended Data Fig. 10c). Patients with the top 50% of OxPhos scores were compared with patients with the bottom 50% in each subcohort. In stage 1/2 tumours, there was no substantial difference in survival for the OxPhos high and low groups (Fig. 5b). However, in the stage 3/4 subset, high tumour OxPhos scores portended poor survival, with the OxPhos-high group having less than half the median survival (878 days) of the OxPhos-low group (1,964 days) (Fig. 5c). Therefore, even among the highest risk patients (with grade 3/4 tumours and 3/4 staging), expression of OxPhos genes is predictive of patient mortality.

In this same TCGA cohort, it was previously reported that higher mtDNA content also correlates with poor survival^23^ (Fig. 5d), even though the vast majority of genes comprising the OxPhos gene signature are in the nucleus. Together, both mtDNA abundance and expression of nuclear-encoded OxPhos genes are elevated in aggressive primary ccRCCs with the worst outcomes. This suggests that the mitochondrial adaptation that allows ccRCCs to metastasize originates in the primary tumour.

## Discussion

We have previously reported that ccRCCs infused with [U-^13^C]glucose generally have low labelling relative to the kidney^1^. We now demonstrated that this is an intrinsic characteristic of ccRCC because low glucose contributions to the TCA cycle are preserved in cultured slices of ccRCC tissue, and not all tumours growing in the kidney display suppressed glucose contribution to the TCA cycle. We also report dysfunction of multiple ETC components manifesting as reduced mitochondrial respiration. This may be related to suppressed ccRCC mtDNA copy number^42^, which predicts that PDH activation would be insufficient to normalize oxidative metabolism in ccRCC. We report higher contributions of glucose to the TCA cycle in metastatic ccRCC than in primary ccRCC. This was observed in both synchronous metastases and asynchronous metastases and in multiple metastatic sites, implying an evolution or selection of mitochondrial function during ccRCC progression in patients.

Evidence from mice indicates that the TCA cycle and OxPhos may promote multiple aspects of cancer progression. Quantitative measurement of TCA cycle flux in orthotopic breast cancer models has reported a large increase in flux after metastasis to the lung^43^. In melanoma, brain metastatic formation and growth in mice are suppressed by ETC complex I inhibition^44^, and expression of pathogenic mtDNA variants with diminished OxPhos decrease metastasis^45^. In a pancreatic ductal adenocarcinoma model, relapse is suppressed and survival is enhanced by ETC inhibition^46^. In acute myelogenous leukaemia, human-derived mouse models with robust OxPhos display resistance to cytotoxic chemotherapy, and resistance is reversed by inhibiting mitochondrial function^47^. In patient-derived B progenitor acute lymphoblastic leukaemia models, clones that relapse have gene expression signatures of mitochondrial metabolism and higher mitochondrial mass than clones that do not relapse^48^. These findings suggest that OxPhos and other aspects of mitochondrial function underlie a program of enhanced fitness that allows some tumour cells to survive various stresses relevant to cancer progression, including stresses related to metastasis.

It is unclear whether tumour cells with variable mitochondrial function at the primary site activate mitochondrial metabolism during metastasis or whether metastasis selects for a pre-existing population of cells with high mitochondrial metabolism. Molecular analyses of human ccRCCs still in the kidney suggest that tumours with a higher capacity for mitochondrial metabolism (that is, higher mtDNA content and higher expression of OxPhos genes) are most likely to result in early mortality. Although these mitochondrial alterations may occur in the primary tumour, our experiments in mice indicate that the benefits of OxPhos are revealed only after escape from the primary tumour. Neither complex I inhibition nor expression of NDI1 or *Lb*NOX affected tumour growth in the kidney, even though all of these factors altered metastatic burden. Because these experiments used athymic mice, the data also suggest that the effect of respiration capacity on metastasis is not primarily related to immunogenicity.

Efforts to suppress cancer progression by targeting mitochondrial metabolism would benefit from understanding both the mechanisms governing variable mitochondrial function in human tumours and how mitochondria affect metastasis. Perhaps the most interesting and important challenge arising from this work is to determine which metabolic effects of mitochondrial function support metastasis. Tumours with low respiration, either from their native metabolic properties or after pharmacological complex I inhibition, still metastasize, so assessing how complex I modulation affects survival merits further examination. Our experiments with *Lb*NOX suggest that maintenance of a favourable NAD^+^/NADH redox balance is sufficient to support metastasis to the lung, but further work is necessary to understand whether additional ETC functions are also required. Potent, systemic blockade of the ETC in patients has resulted in dose-limiting toxicities^49^, but it may be possible to widen the therapeutic window by tailoring therapies to selectively target the most relevant aspects of mitochondrial function.

## Methods

### Patient infusions

The Institutional Review Board (IRB) at the University of Texas Southwestern Medical Center monitored and approved all conducted human participant research. Study protocols are subjected to annual continuing reviews by the IRB. All patients were recruited at the University of Texas Southwestern Medical Center. From December 2018 to August 2019, patients were enrolled on protocol STU062010-157. From September 2019 to present, patients are enrolled on protocol STU2019-1061. Two patients were enrolled on protocol STU052012-065. Generally, patients 18 years of age or older with radiographic evidence of known or probable kidney cancer requiring surgical biopsy or excision were recruited to an IRB-approved study and informed consent was obtained from all patients. Full eligibility and exclusion criteria, as well as recruitment procedures, are detailed in the Reporting Summary. Patients receiving [U-^13^C]glucose were enrolled on protocol STU062010-157 (NCT01668082), STU2019-1061 (NCT04623502) or STU052012-065 (NCT02095808) and infused at the following rate: 8 g bolus of [U-^13^C]glucose administered over 10 min, followed by a continuous infusion of [U-^13^C]glucose at a rate of either 4 or 8 g per hour. Patients receiving [1,2-^13^C]acetate were enrolled on protocol STU2019-1061 and were infused at the following rate: a bolus of 3 mg [1,2-^13^C]acetate per kg body weight per minute for 5 min, followed by a continuous infusion of [1,2-^13^C]acetate at 1.5 mg per kg body weight per minute. Patients receiving [U-^13^C]glutamine were enrolled on protocol STU2019-1061 and were infused at the following rate: a primer dose for 5 min at a rate of 0.6 mg per kg body weight per minute, followed by a continuous infusion of 5.0 μmol per kg body weight per minute (0.73 mg per kg body weight per minute). Uncontrolled or poorly controlled diabetes and pregnancy were exclusion criteria for the study. Demographic, clinical and pathological details are summarized in Supplementary Data Table 1. Once the specimens were removed from the body cavity, tissue samples were acquired with the attending pathologist or pathology assistant.

### Animal studies

All procedures were approved by UT Southwestern Medical Center’s Animal Care and Use Committee (protocol 2016-101360) or the University of Texas MD Anderson Cancer Center’s Animal Care and Use Committee (protocol 00001158) in accordance with the Guide for the Care and Use of Laboratory Animals. Sample sizes were not pre-determined based on statistical power calculations but were based on our experience with these assays. All mice were given a numerical identifier, and tumour measurements were recorded according to the numerical identifier and unblinded at the end of the study. Mouse experiments requiring pharmacological treatments were not blinded to monitor mice for side effects. Mice were randomly allocated for injection and/or treatment.

### Cell lines

786-O, Caki-1 and primary renal proximal tubule epithelial cells were purchased from the American Type Culture Collection (ATCC; CRL-1932, HTB-46 and PCS-400-010, respectively) and were authenticated by the ATCC. HEK293FT cells were purchased from Thermo Fisher (R70007) and were not authenticated after purchase. Met^low^, Met^high^, Met^high^-50 and Met^high^-26 were established from somatic mosaic genetically engineered mouse models (SM-GEMM) of kidney cancer^36^. Met^low^ and Met^high^ cell lines were established from *Setd2*^*KO*^*;NF2*^*KO*^*;4q*^*9p21−*^ mice, and Met^high^-50 and Met^high^-26 cell lines were established from *Setd2*^*KO*^*;Vhl*^*KO*^*;4q*^*9p21−*^ mice. The Met^low^ cell line originated from a SM-GEMM mouse with no metastases, and the Met^high^, Met^high^-50 and Met^high^-26 lines originated from SM-GEMM mice with ten or more metastases.

The primary kidney tumours were dissociated with a combination of mechanical dissociation and enzymatic dissociation using a 2 mg ml^−1^ mixture of collagenase IV/dispase (17104-019 and 17105-041, respectively, Invitrogen) resuspended in DMEM. Tissues were incubated for 1 h at 37 °C with trituration every 15 min. Cells were then plated on plates coated with 0.1% gelatin and cultured in DMEM supplemented with 20% FBS and 1% penicillin–streptomycin. To confirm metastatic potential, cells were reinjected under the renal capsule of NU-*Foxn1*^*nu*^ mice as described in the ‘Metastatic colonization experiments using mouse cell lines in mice’ section, and mice were analysed for metastatic burden after 21 days. Primary tumours with high or low metastatic burden were dissociated and reinjected again under the renal capsule to select for differential metastatic capacity. Primary tumours that gave rise to a high number of metastatic nodules were termed Met^high^, and clones that gave rise to fewer metastases were termed Met^low^. The Met^high^, Met^high^-50, Met^high^-26 and Met^low^ cell lines reflected the metastatic potential of their SM-GEMM of origin. Cells were kept in culture for five passages or less before re-implantations.

All cell lines were confirmed to be mycoplasma free using a commercial kit (2523348, Bulldog Bio). Cells were maintained in RPMI supplemented with 10% fetal bovine serum or 10% dialysed human serum and cultured at 37 °C in 5% CO_2_ and 95% air, unless otherwise noted.

### High-resolution mass spectrometry (Q-TOF)

Data acquisition from isolated mitochondria and patient tissues for metabolomics was performed by reverse-phase chromatography on a 1290 UHPLC liquid chromatography system interfaced to a high-resolution mass spectrometry (HRMS) 6550 iFunnel Q-TOF mass spectrometer (Agilent Technologies). Frozen tissue fragments weighing 10–30 mg were added to ice-cold 80:20 methanol:water and extracted for metabolomics analysis. Samples were subjected to three freeze–thaw cycles, then centrifuged at 16,000*g* for 20 min to precipitate macromolecules. The supernatant was evaporated using a vacuum concentrator. Samples were resuspended in 100 μl of 0.1% formic acid in water, vortexed for 30 s and centrifuged at 16,000*g* for 15 min. Supernatant was transferred to an autosampler vial and then run on the mass spectrometer. The mass spectrometer was operated in both positive and negative (ESI^+^ and ESI^−^) modes. Analytes were separated on an Acquity UPLC HSS T3 column (1.8 μm, 2.1 × 150 mm; Waters). The column was kept at room temperature. Mobile phase A composition was 0.1% formic acid in water, and mobile phase B composition was 0.1% formic acid in 100% acetonitrile. The liquid chromatography gradient was 1% phase B (5 min), 5% phase B (10 min), 99% phase B (8 min), 99% phase B (1 min), 1% phase B (1 min) and 1% phase B (1 min). The flow rate was 250 μl min^−1^. The sample injection volume was 5 μl.

ESI source conditions were set as follows: dry gas temperature of 225 °C and flow of 18 l min^−1^, fragmentor voltage of 175 V, sheath gas temperature of 350 °C and flow of 12 l min^−1^, nozzle voltage of 500 V and capillary voltage of +3,500 V in positive mode and −3,500 V in negative mode. The instrument was set to acquire over the full *m/z* range of 40–1,700 in both modes, with the mass spectrometer acquisition rate of 1 spectrum per second in profile format.

Raw data files (.d) were processed using Profinder B.08.00 SP3 software (Agilent Technologies) with an in-house database containing retention time and accurate mass information from 600 standards from Mass Spectrometry Metabolite Library (IROA Technologies). The database was created under the same data acquisition conditions described above. The in-house database matching parameters were: mass tolerance of 10 ppm and retention time tolerance of 0.5 min. Peak integration results were manually reviewed in Profinder and exported as a spreadsheet (.csv).

### Metabolomics analysis

The signal-to-noise ratio for each metabolite was calculated by dividing the median of the quality control samples by the median of the blank samples. Following this, a run order correlation test was performed to determine the correlation between the run order and metabolite intensity for quality control samples injected at fixed intervals, adjusting for multiple comparisons using the Benjamini–Hochberg procedure. A LOESS (locally estimated scatterplot smoothing) correction was then applied to adjust for trends in the data that correlate with run order, which involved fitting a LOESS model to the quality control data and adjusting the sample data accordingly only if the trend in quality control samples within the interquartile range was statistically significant. Normalization was then performed using total ion count, using a subset of stable metabolites with low coefficient of variation and high signal-to-noise ratio to calculate a normalization factor based on their median intensities. This was used to adjust the intensity values of all metabolites in a sample. After normalization, a log_10_ transformation was applied to obtain a normal distribution. In cases in which a metabolite was measured in both positive and negative modes, the measurement with the lower signal-to-noise ratio was removed.

We compared our metabolomics dataset to a previously reported ccRCC dataset^14^. The value of each metabolite was normalized to the minimum observed value across the dataset. A log_10_ transformation was applied to the normalized values to stabilize variance and render the data more suitable for parametric analysis. Cohen’s *d* was used to assess the differential abundance of metabolites between tumour and adjacent normal tissues. For integration and comparison with the Hakimi dataset, we standardized the metabolite nomenclature by mapping the metabolite names to their respective Human Metabolome Database (HMDB) identifiers^50^. For the 152 metabolites that were common between both studies, the Pearson correlation coefficient of the effect sizes was calculated to assess the concordance of datasets.

Raw metabolomics files are available at the US National Institutes of Health Common Fund’s National Metabolomics Data Repository website, the Metabolomics Workbench^51^ (https://www.metabolomicsworkbench.org/), where it has been assigned project ID PR001954. A direct link to the data can be found in the ‘Data availability’ section.

### HRMS (Orbitrap)

[1,2-^13^C]acetate-infused tissue samples were analysed using an Orbitrap Fusion Lumos 1M Tribrid Mass Spectrometer. HILIC chromatographic separation of metabolites was achieved using a Millipore ZIC-pHILIC column (5 μm, 2.1 × 150 mm) with a binary solvent system of 10 mM ammonium acetate in water, pH 9.8 (solvent A), and acetonitrile (solvent B) with a constant flow rate of 0.25 ml min^−1^. For gradient separation, the column was equilibrated with 90% solvent B. After injection, the gradient proceeded as follows: 0–15 min of linear ramp from 90% B to 30% B; 15–18 min of isocratic flow of 30% B; 18–19 min of linear ramp from 30% B to 90% B; 19–27 column regeneration with isocratic flow of 90% B. HRMS data were acquired with two separate acquisition methods. Individual samples were acquired with an HRMS full scan (precursor ion only) method, switching between positive and negative polarities. For data-dependent, high-resolution tandem mass spectrometry methods, precursor ion scans were acquired at a resolving power of 120,000 full width at half-maximum (FWHM) with a mass range of either 50–750 or 70–1,050 Da. The AGC target value was set to 1 × 10^6^ with a maximum injection time of 100 ms. Pooled samples were generated from an equal mixture of all individual samples and analysed using individual positive-polarity and negative-polarity spectrometry data-dependent, high-resolution tandem mass spectrometry acquisition methods for high-confidence metabolite ID. Product ion spectra were acquired at a resolving power of 15,000 FWHM without a fixed mass range. The AGC target value was set to 2 × 10^5^ with a maximum injection time of 150 ms. Data-dependent parameters were set to acquire the top ten ions with a dynamic exclusion of 30 s and a mass tolerance of 5 ppm. Isotope exclusion was turned on, and a normalized collision energy value of 30% was used or a stepped normalized collision energy applied with values of 30%, 50% and 70%. Settings remained the same in both polarities. Metabolite identities were confirmed in three ways: (1) precursor ion *m*/*z* was matched within 5 ppm of theoretical mass predicted by the chemical formula; (2) fragment ion spectra were matched within a 5 ppm tolerance to known metabolite fragments; and (3) the retention time of metabolites was within 5% of the retention time of a purified standard run with the same chromatographic method. Metabolites were relatively quantitated by integrating the chromatographic peak area of the precursor ion searched within a 5 ppm tolerance.

Acetyl-CoA fractional enrichment was determined with a selected ion monitoring (SIM) scan event on an Orbitrap Fusion Lumos 1M Tribrid Mass Spectrometer. The SIM scan event targeted the theoretical mass for the positive ion of acetyl-CoA in positive ionization mode (*m*/*z* 810.1330) with a 4.5-Da window. Data were collected with a resolving power of 60,000 FWHM with an AGC target of 4 × 10^5^ ions. To calculate fractional enrichment of m+2 acetyl-CoA, the SIM scan integrated the m+0, m+1 and m+2 peaks and the full-scan data to integrate the remaining naturally abundant isotopes. Isotope enrichment was corrected for natural abundance.

### Mass spectrometry for isotopomer analysis

Samples were analysed on an AB Sciex 6500 QTRAP liquid chromatography/mass spectrometer (Applied Biosystems SCIEX) equipped with a vacuum degasser, quaternary pump, autosampler, thermostatted column compartment and triple quadrupole/ion trap mass spectrometer with electrospray ionization interface, and controlled by AB Sciex Analyst 1.6.1 Software. SeQuant ZIC-pHILIC 5-µm polymer (150 × 2.1 mm) columns were used for separation. Solvents for the mobile phase were 10 mM ammonium acetate aqueous (pH 9.8 adjusted with NH_3_•H_2_O (A) and pure acetonitrile (B). The gradient elution was: 0–20 min, linear gradient of 90–65% B; 20–23 min, linear gradient of 65–30% B; 23–28 min, 30% B; and 28–30 min, linear gradient of 30–90% B, then reconditioning the column with 90% B for 5 min. The flow rate was 0.2 ml min^−1^ and the column was operated at 40 °C. Glutamate isotopomers were analysed using a published method^17^.

### Gas chromatography–mass spectrometry

Gas chromatography–mass spectrometry was used to analyse infused patient tissue and plasma samples, as well as tracing assays in cell lines and slice cultures. Blood was obtained before and approximately every 30 min during infusion, when congruent with surgical workflow, until tissue was removed from the patient. Whole blood was chilled on ice and centrifuged (at 1,500*g* for 15 min at 4 °C, with acceleration and deceleration rates set to 5) to separate and freeze the plasma. Aliquots (50 µl) of plasma were added to ice-cold 80:20 methanol:water for extraction. Frozen tissue fragments weighing roughly 10–30 mg were added to ice-cold 80:20 methanol:water and extracted to analyse ^13^C enrichment. Samples were subjected to three freeze–thaw cycles, then centrifuged at 16,000*g* for 20 min to precipitate macromolecules. The supernatant was evaporated using a vacuum concentrator and resuspended in 30 μl of methoxyamine (10 mg ml^−1^) in pyridine. Samples were transferred to autoinjector vials and heated at 70 °C for 15 min. A total of 70 μl of tert-butyldimethylsilyl was added, and the samples were briefly vortexed and heated for another 60 min at 70 °C. Injections of 1 μl were analysed on an Agilent 7890A gas chromatograph coupled to an Agilent 5975C mass selective detector. The observed distributions of mass isotopologues were corrected for natural abundance^52^.

### mtDNA:nuclear DNA quantitative PCR

Genomic DNA was isolated using the DNeasy Blood & Tissue Kit (69504, Qiagen). Samples were run using the Luna Universal One-Step qPCR Kit (M3003, New England Biolabs) on a CFX384 machine (Bio-Rad). The following primers were used for human *COX2* as representative of mtDNA: CCGTCTGAACTATCCTGCCC (forward), (GCCGTAGTCGGTGTACTCGT (reverse). The following primers were used for human histone 3 (*H4C3*) as representative of nuclear DNA: GGGATAACATCCAGGGCATT (forward), CCCTGACGTTTTAGGGCATA (reverse).

### RNA isolation

RNA was isolated using TRIzol (15596018, Thermo Fisher) and an RNeasy Mini Kit (74106, Qiagen). Total RNA was quantified using a Qubit fluorometer (Invitrogen) and the Invitrogen Qubit RNA High Sensitivity kit (Q32852, Invitrogen). Samples were diluted in ultrapure water before sequencing.

### *NDI1* RT–quantitative PCR

RNA was isolated as described above and samples were run using the Luna Universal One-Step RT–qPCR Kit (E3005, New England Biolabs) on a CFX384 machine (Bio-Rad). The following primers were used for *NDI1*: GCCGAAGAAGTCCAAATTCAC (forward), CGACAGCCGTTCTCAGAT (reverse). The following primers were used for *ACTB* (encoding β-actin): CTAAGGCCAACCGTGAAAAG (forward), ACCAGAGGCATACAGGGACA (reverse).

### RNA sequencing

RNA sequencing libraries were prepared using the NEBNext Ultra II directional RNA library prep kit with the NEBNext poly(A) mRNA magnetic isolation module (E7490L and E7760L, New England Biolabs) according to manufacturer’s instructions. Libraries were stranded using standard NEB indices according to the manufacturer’s instructions (E7730L, E7335L and E7500L, New England Biolabs). Sequencing reads were aligned to the human reference genome (*hg19*) by STAR 2.7.3.a with default parameters in the two-pass mode. Counts for each gene were generated using htseq-count v0.6.1. Differentially expressed genes were identified by DESeq2 v1.14.1. The ends of sequences were trimmed with remaining adapter or quality scores of less than 25. Sequences less than 35 bp after trimming were removed. The trimmed Fastq files were aligned to GRCh38 using HiSAT2 (ref. ^53^) and duplicates were marked with SAMBAMBA. Features (genes, transcripts and exons) were counted using featureCounts^54^. Differential expression analysis was performed using EdgeR^55^ and DESeq^56^. Raw sequencing files are deposited on the Gene Expression Omnibus (GSE251905). Extended Data Fig. 1a compares RNA sequencing data from the TCGA cohort and this study, emphasizing genes related to the ETC and glycolysis. The Cohen’s effect size (*d*) between tumour and adjacent kidney for each of 15,642 genes was correlated between TCGA data and data from the current cohort. ETC genes were selected from the gene ontology cellular component library, including genes related to complexes I–IV of the ETC. The glycolysis genes include the following four gene sets: KEGG_GLYCOLYSIS_GLUCONEOGENESIS, REACTOME_GLYCOLYSIS, HALLMARK_GLYCOLYSIS and WP_GLYCOLYSIS_AND_GLUCONEOGENESIS.

### ESTIMATE analysis

The ESTIMATE R package^57^ was used to derive stromal and immune scores and a tumour purity ESTIMATE score from RNA sequencing data.

### OxPhos score calculation

A list of OxPhos genes from the MSigDB database^58^ was created by combining the genes in the KEGG_OXIDATIVE_PHOSPHORYLATION gene set with the genes encoding subunits of the PDH complex (*PDHA1*, *PDHB*, *DLAT*, *DLD* and *PDHX*). Principal component analysis was performed on log_2_-transformed, mean-centred and *z*-transformed data, and the first principal component (PC1) was extracted and used as the OxPhos score.

### Organotypic slice cultures

After surgery, kidney cortex and tumour fragments were embedded in 0.1% agarose (BP1356, Thermo Fisher) and sliced into approximately 300-μM thick sections using a microtome (Compresstome, VF-300, Precisionary Instruments). These tissues were then transferred and maintained on hydrophilic polytetrafluoroethylene cell culture inserts (PICM0RG50, Millipore) in human plasma-like medium supplemented with 10% dialysed human serum. Before tracing assays, tissues were washed twice with 0.9% saline, and medium was replaced with human plasma-like medium without unlabelled glucose and replaced with an equivalent concentration of [U-^13^C]glucose for 3 h. Slices were maintained in an incubator with 5% CO_2_, 5% O_2_ and 90% N_2_ at 37 °C.

### Human serum dialysis

Human serum was purchased from Sigma-Aldrich (H3667) and dialysed using SnakeSkin dialysis tubing, 3.5 K MWCO, 35 mm (PI88244, Thermo Fisher). Serum was dialysed against a 20X volume of PBS. Dialysis was performed for 48 h at 4 °C with a complete PBS exchange every 9–12 h. Dialysed serum was then sterile filtered using bottle-top vacuum filters with a pore size of 0.22 μm (431097, Corning).

### Mitochondrial isolation and respiration measurements

OCRs were measured using a Seahorse Xfe96 Analyzer (Agilent Technologies) as previously described^59,60^. Fresh kidney and tumour samples were homogenized with 40 strokes of a Dounce homogenizer in mitochondrial isolation buffer consisting of 5 mM HEPES, 70 mM sucrose, 220 mM mannitol, 5 mM MgCl_2_, 10 mM KH_2_PO_4_ and 1 mM ethylene glycol-bis(β-aminoethyl ether)-*N*,*N*,*N*′,*N*′-tetraacetic acid (EGTA), pH 7.2; H4034, S0389, M4125, 208337, P9541 and E3889, respectively, Sigma-Aldrich) and isolated via differential centrifugation at 4 °C. Nuclei and cell debris were removed by centrifuging five times at 600*g*. Mitochondria were pelleted with a 10,000*g* spin and washed twice. Mitochondria were quantified using a detergent compatible assay (5000112, Bio-Rad).

Outer mitochondrial membrane fidelity was tested using a proteinase K assay as previously described^61^. In brief, 50 μg mitochondria were incubated on ice for 10 min with proteinase K (AM2542, Thermo Fisher) at a final concentration of 10 μg ml^−1^. The reaction was stopped by pelleting mitochondria and resuspending in lysis buffer (2% SDS and 25 mM Tris. pH 6.8) with 1 mM phenylmethylsulfonyl fluoride (PMSF; 36978, Thermo Fisher) and immediately snap frozen in liquid nitrogen. Immunoblots were then run on the proteinase K-treated mitochondria as described in the ‘Immunoblotting’ section below for both an outer mitochondrial membrane protein (anti-Tom20, 1:2,000 dilution; 11802, Proteintech) and a mitochondrial matrix protein (anti-HSP60, 1:2,000 dilution; 12165S, Cell Signaling Technology). For experiments reported in this paper, we only used respiration and tracing data from mitochondria with an intact outer mitochondrial membrane (as evidenced by an intact, non-smeared HSP60 band after proteinase K treatment).

Of mitochondria, 5 µg was plated in an Xfe96 plate on ice and centrifuged at 2,700*g* for 2 min at 4 °C. Isolation buffer containing ETC complex substrates was added to cells and measurements were started immediately. The following ETC complex substrates were used: complex I–pyr/mal (10 mM pyruvate and 1 mM malate), complex I—glu/mal (10 mM glutamate and 1 mM malate), complex II (5 mM succinate and 4 μM rotenone) and complex IV (10 mM ascorbate, 100 μM TMPD and antimycin A 2 μM). At indicated times, 4 mM ADP, 2 μM oligomycin A, 2 μM carbonyl cyanide 3-chlorophenylhydrazone (CCCP) and either 4 μM antimycin A or 40 μM sodium azide were injected. All chemicals were purchased from Sigma-Aldrich (P2256 (pyruvate), 240176 (malate), 49621 (glutamate), S3674 (succinate), R8875 (rotenone), A4403 (ascorbate), T7394 (TMPD), A8674 (antimycin A), A5285 (adenosine diphosphate), 75351 (oligomycin A), C2759 (CCCP) and S2002 (sodium azide)). All indicated concentrations are final concentrations. Respiratory control ratios were calculated by state III/state IV respiration.

For cells expressing the pMXs-EV or pMXs-NDI1 retroviral vector, cells were plated in a 96-well plate at a concentration of 2 × 10^4^ cells per well in 80 μl RPMI-1640 media with 4 mM glutamine and 10% FBS. Cells were incubated in a CO_2_-free incubator at 37 °C for 1 h before Xfe96 measurements to allow temperature and pH equilibration. Seahorse assays consisted of three mix (3 min) and measurement (3 min) cycles, allowing determination of OCR and the extracellular acidification rate every 6 min.

### NDI1, *Lb*NOX and dsRed luciferase expression

pMXs-EV, pMXs-mito-*Lb*NOX and pMXs-cyto-*Lb*Nox were a gift from J. Garcia-Bermudez^62^. pMXs-NDI1 was a gift from D. Sabatini (plasmid 72876, AddGene)^63^. pMXs-EV, pMXs-NDI1, pMXs-mito-*Lb*NOX or pMXs-cyto-*Lb*NOX was transfected into 293FT cells with gag-pol and VSVG using Lipofectamine 3000 (L3000015, Thermo Fisher) or PolyJet (SL100688, SignaGen) according to the manufacturer’s instructions. Viral supernatants were collected 48 h after transfection and filtered through a 0.45-μm filter. Cells were cultured with virus containing media and 4 µg ml^−1^ polybrene (TR-1003-G, Sigma-Aldrich) for 24 h, after which fresh medium was added. Cells were then exposed to 10 µg ml^−1^ blasticidin selection until uninfected cells died.

A bi-cistronic lentiviral construct carrying dsRed2 and luciferase (dsRed2-P2A-Luc) was a gift from S.J. Morrison. dsRed2-P2A-Luc with pMD2G and psPAX2 were transfected into 293FT cells using Polyjet (SL100688, SignaGen) according to the manufacturer’s instructions. Viral supernatants were collected 48 h after transfection and filtered through a 0.45-μm filter. Cells with either pMXs-EV, pMXs-NDI1, pMXs-cyto-*Lb*NOX or pMXs-mito-*Lb*NOX were cultured with virus containing media and 4 µg ml^−1^ polybrene (TR1003, Sigma-Aldrich) for 8 h, after which the medium was changed to fresh medium. The top 10% of live cells expressing dsRed were sorted using fluorescence-activated cell sorting (FACS). 786-O and Caki-1 cells expressing dsRed were trypsinized and filtered through a 40-μm cell strainer to obtain a single-cell suspension and stained with 4′,6-diamidino-2-phenylindole (DAPI; 62248, Thermo Fisher). Cells were gated to exclude dead cells, cell debris and doublets based on FSC/SSC, then gated on live cells (DAPI-negative cells). The top 10% of dsRed-positive cells were sorted, collected and used for experiments. Example FACS plots exemplifying the gating strategy are provided in Supplementary Fig. 2. Data were analysed using BD FACSDiva 8.0 and FlowJo V10.

### Immunofluorescence and confocal microscopy

Coverslips were coated with 10 µg ml^−1^ fibronectin (F1141, Sigma-Aldrich) for 1 h at 37 °C and rinsed once with PBS. Cells were immediately seeded on the coverslips and fixed the next day with fresh warm 4% paraformaldehyde solution in PBS for 15 min followed by permeabilization using 0.1% (v/v) Triton X-100 in PBS at room temperature for 10 min. Cells were blocked in filtered PBS containing 1% BSA for at least 30 min at room temperature before incubation with primary antibodies to FLAG (1:200 dilution; F1804, Sigma-Aldrich) and HSP60 (1:500 dilution; 12165S, Cell Signaling Technology) for 1 h at room temperature. Cells were washed three times for 5 min with PBS and incubated with fluorophore conjugated secondary antibodies (1:500 dilutions; 111-545-144, Jackson ImmunoResearch Laboratories and A31570, Thermo Fisher) for 1 h at room temperature in the dark. Coverslips were washed with PBS three times for 5 min and Mili-Q water once before being mounted on Profade-Antifade (P36935, Invitrogen) slides overnight in the dark. Cells were imagined using a Zeiss LSM 880 confocal laser scanning microscope with Z-stacks acquired. All representative images were processed using ImageJ.

### Ki67 staining

After removal of tumour-bearing organs, tissues were fixed in 10% formalin, sectioned and stained for Ki67-positive nuclei (1:500 dilution; MA5-14520, Thermo Fisher). Slides were imaged using an inverted Zeiss LSM780 confocal microscope. Ki67-positive nuclei were quantified using ImageJ.

### Immunoblotting

Cells were lysed in RIPA buffer (BP-115, Boston BioProducts) containing protease and phosphatase inhibitors (78444, Thermo Fisher), then centrifuged at 4 °C for 10 min at approximately 20,160*g*. Supernatants were transferred to new pre-chilled 1.5-ml tubes, and protein concentrations were quantified using a BCA Assay Kit (23225, Thermo Fisher). Protein lysates were resolved via SDS–PAGE and transferred to PVDF membranes. Membranes were blocked in 5% BSA in Tris buffered saline with Tween-20 (TBST; 20 mM Tris (pH 7.5), 150 mM NaCl and 0.1% Tween-20) and then incubated with primary antibody (anti-FLAG M2, 1:2,000 dilution; F1804, Sigma-Aldrich) in TBST supplemented with 5% BSA at 4 °C overnight. The primary antibody was detected with a horseradish peroxidase-conjugated secondary antibody (1:2,000 dilution; 7076S, Cell Signaling Technology) for 1 h followed by exposure to ECL reagents (PI32106, Fisher Scientific). After imaging, membranes were stripped with Restore Striping Buffer (21059, Thermo Fisher), washed with TBST three times and blocked with 5% BSA in TBST for 1 h. Membranes were then incubated with primary antibody (β-actin, 1:2,000 dilution; 8457S, Cell Signaling Technology) in TBST supplemented with 5% BSA at 4 °C overnight. The primary antibody was detected with a horseradish peroxidase-conjugated secondary antibody (1:2,000 dilution; 7074S, Cell Signaling Technology) for 1 h followed by exposure to ECL reagents (PI32106, Thermo Fisher).

### [U-^13^C]glucose tracing in cell lines

[U-^13^C]glucose tracing data from non-small-cell lung cancer cell lines were previously reported^41^. Similar assay conditions described below were used for tracing experiments in this study. Before tracing experiments, cells expressing either empty vector or NDI1 were washed twice with 0.9% saline, and medium was replaced with RPMI-1640 containing [U-^13^C]glucose supplemented with 5% dialysed FBS for 6 h. Cells were rinsed in ice-cold 0.9% saline and lysed with three freeze–thaw cycles in ice-cold 80% methanol. Samples were then prepared for gas chromatography–mass spectrometry analysis.

### Subcutaneous implantation of human cell lines in mice

Cell suspensions were prepared for injection in a 1:1 mixture of Matrigel (354234, Corning) to staining medium (Leibovitz’s L15 medium (21083027, Thermo Fisher), 1 mg ml^−1^ BSA (A2153, Sigma-Aldrich), 1% penicillin–streptomycin (P0781, Sigma-Aldrich) and 10 mM HEPES (pH 7.4)). Subcutaneous injections were performed in NOD.CB17-*Prkdc*^*scid*^ *Il2rg*^*tm1Wjl*^/SzJ (NSG) mice in a final volume of 100 μl. Four-to-eight-week-old male and female NSG mice were transplanted with 1,000,000 cells subcutaneously in the right flank. Both male and female mice were used. For all subcutaneous experiments, the maximum permitted tumour diameter was 2.0 cm, which was not exceeded in any experiment.

### Metastatic colonization experiments using human cell lines in mice

Cell suspensions were prepared for injection in staining medium described above. Tail-vein injections were performed in NOD.CB17-*Prkdc*^*scid*^*Il2rg*^*tm1Wjl*^/SzJ (NSG) mice in a final volume of 50 μl. Four-to-eight-week-old male and female NSG mice were transplanted with 250,000 cells. Both male and female mice were used. Metastatic burden was assessed weekly by bioluminescence. Five minutes before performing luminescence imaging, mice were injected intraperitoneally with 100 μl of PBS containing 40 mg ml^−1^ d-luciferin monopotassium salt (L8220, Biosynth) and mice were anaesthetized with isoflurane 2 min before imaging. The mice were imaged using an IVIS Imaging System 200 Series (Caliper Life Sciences) or an Ami HTX (Spectral Instruments Imaging). The exposure time ranged from 10 to 60 s, depending on the maximum signal intensity, with the 60-s timepoint used for greater range only if the 30-s timepoint was not saturated. The bioluminescence signal (total photon flux) was quantified with ‘region of interest’ measurement tools in Living Image software (Perkin Elmer) or Aura Imaging software (Spectral Instruments Imaging). The maximal tumour burden was defined as a bioluminescent signal saturated after 30 s, which was not exceeded during any experiment.

### Metastatic colonization experiments using mouse cell lines in mice

Maximal tumour burden was not exceeded according to the IRB guidelines. For orthotopic tumours, mice were euthanized 21 days after implantation or after they developed symptoms of distress. Both male and female mice were used. Mice were kept in a 12-h light–12-h dark cycle as commonly used, and housed at 18–23 °C with humidity of 50–60%.

To establish orthotopic tumours, 10,000 cells were resuspended in a 2:1 solution of OPTI-MEM (31985062, Gibco) and Matrigel (354234, Corning). Six-to-nine-week-old NU-*Foxn1*^*nu*^ (Jackson Laboratories) mice were anaesthetized using isoflurane. Buprenorphine slow release (0.1 mg kg^−1^ two times daily) was subcutaneously injected, and shaved skin was disinfected with 70% ethanol and betadine (1425, Dynarex). A 1-cm incision was performed on the left flank through the skin/subcutaneous and muscular/peritoneal layers. The left kidney was exposed and 20 μl of the cell suspension was injected under the kidney capsule. The kidney was repositioned into the abdominal cavity, and muscular/peritoneal planes were closed individually by absorbable sutures. The skin/subcutaneous planes were closed using metal clips. Mice were monitored daily for the entire duration of the experiment. Twenty-one days after orthotopic kidney implantation, mice were imaged for primary tumour burden in the kidney and metastatic tumour burden in the lungs. A 7T Bruker Biospec (BrukerBioSpin), equipped with a 35-mm inner-diameter volume coil and 12-cm inner-diameter gradients, was used for MRI. A fast acquisition with relaxation enhancement sequence with 2,000/39-ms TR/TE (repetition time/echo time), 256 × 192 matrix size, r156-µM resolution, 0.75-mm slice thickness, 0.25-mm slice gap, 40 × 30-cm^2^ field of view, 101-kHz bandwidth and 4 number of excitation was used to acquire multislice T2-weighted images in coronal and axial planes. At end point, mice were euthanized by exposure to carbon dioxide followed by cervical dislocation. A necropsy form was filled in with mouse information, tumour size, and metastasis location and number. Fluorescent and brightfield images were acquired through a Leica MZ12s stereo microscope. TdTomato-positive lesions were quantified using LAS v4.13 software (Leica Microsystems).

### IACS-010759 treatment

Mice were allowed to recover from implantation surgery. The following day, mice were treated with either 100 μl of vehicle or IACS-010759 (S8731, Selleckchem) dissolved in vehicle via oral gavage on a 5-day on, 2-day off schedule. The vehicle was composed of 5% DMSO, 40% PEG300, 5% Tween-80, and 50% ddH2O. IACS-010759 was dosed at 5 mg kg^−1^. Mice were euthanized after 21 days in accordance with IRB guidelines.

### CRISPR screening in Met^high^ and Met^low^ cells

Lentiviral particles of the mouse genome-wide CRISPR library (mTKOv3) were generated by the University of Michigan Biomedical Research Lentiviral Core and concentrated 100×. Cells were transduced with the mouse genome-wide CRISPR library in 500-cm^2^ square dishes with 8 μg ml^−1^ polybrene at a multiplicity of infection of 0.3 and an estimated 400× coverage. The medium was replaced 24 h after infection, and puromycin selection started at 48 h. At 72 h, cells were trypsinized, pooled and counted. As a reference, 30 × 10^6^ cells were immediately collected. Every passage of 15 × 10^6^ cells (approximately 200× coverage) was maintained in culture until the end point (20 doublings) when 30 × 10^6^ cells (approximately 400× coverage) were collected.

Cell pellets were suspended in 2 ml of buffer P1/RNAse A and lysed by adding 1/20 volume of 10% SDS. Genomic DNA was sheared by passing the lysate 10–15 times through a 22-gauge syringe needle after 10 min. One volume of phenol:chloroform:isoamyl alcohol (25:24:1) was added to the lysate, and the samples were centrifugated at 17,000*g* for 10 min, and the upper phase was transferred to a new tube. The second extraction step was done with chloroform:isoamyl alcohol (24:1), and the upper phase was transferred to a new tube and mixed with 0.1 volumes of 3 M NaCl and 0.8 volumes of 2-propanol to precipitate genomic DNA. Samples were centrifuged at 17,000*g* for 20 min at 4 °C, and the DNA pellet was washed in 70% ethanol and centrifuged for 5 min at 17,000*g* at 4 °C. The DNA pellet was then dried and resuspended overnight in UltraPure distilled water. The genomic DNAs were quantified using a NanoDrop 2000 (Thermo Fisher). For the generation of next-generation sequencing libraries, barcodes were amplified in two rounds of PCR using the Titanium Taq DNA polymerase (639208, Clontech-Takara). The first PCRs contained 10 µg of genomic DNA per PCR, and the total reactions resulted in targeted amplification from one-third of the total genomic DNA. The first 16 cycles targeted PCR amplification and utilized the following primer set: mTKOv3-PCR1-F: ATTAGTACAAAATACGTGACGTAGAA (forward) and mTKOv3-PCR1-R: ACCTTCTCTAGGCACCGGATCA (reverse). The second PCRs were performed for 14 cycles using the following primers with adapters optimized to introduce the specific adapters for Illumina next-generation sequencing technology specific for the Hiseq4000: mTKO-P2: AATGATACGGCGACCACCGAGATCTACACGAGATCGGACTATCATATGCTTACCGTAACTTGAA (forward) and mTKO-P7##-IND: CAAGCAGAAGACGGCATACGAGATGCACGACGAGACGCAGACGAAnnnnnAGAGCAACTTCTCGGGGACTGTGGGCGA. Amplified PCR products from two replicates of the second PCR were pooled together and extracted from the agarose gel with the QIAquick gel extraction kit (28704, Qiagen). Samples were quantified using Qubit 2.0 DNA HS Assay (Q32851, Thermo Fisher), QuantStudio 5 System (Applied Biosystems) and Tapestation High Sensitivity D1000 Assay (5067-5582, Agilent Technologies). Six samples were pooled equilmolar to be run on a Nextseq 500 high-output 75-bp SR with 10% PhiX. Custom primers were required for read 1 (20 nt): mTKO-Seq-26bp TCTTGGCTTTATATATCTTGTGGAAAGGACGAAACACCG; and to obtain the sample index, read 2 (6 nt): mTKO-Seq-Index-7 AGATGCACGACGAGACGCAGACGAA.

### CRISPR screen analysis

Bowtie63 was used to obtain raw read-counts with 1 mismatch allowance, thus taking the best-matching single guide RNA per read. Bayesian Analysis of Gene EssentiaLity 2 (BAGEL2) software^64^ was then used to calculate normalized read counts, and log_2_ fold change was obtained by comparing the reference timepoint of the corresponding cell line. Next, genes were determined as vulnerabilities by applying the standard BAGEL2 pipeline and excluding core-essential genes. The top 500 gene vulnerabilities ranked by BAGEL score were used as an input for enrichment pathway analysis using the WEB-based GEne SeT AnaLysis Toolkit (WebGestalt)^65,66^. The following parameters were used for WebGestalt: minimum number of genes per category = 5; maximum number of genes per category = 2,000; and test performed = Benjamini–Hochberg.

### Statistical analysis and reproducibility

Samples were analysed as described in the figure legends. Data were considered significant if *P* < 0.05. No statistical methods were used to predetermine sample size. For randomization and blinding details, please see the Reporting Summary. Most experiments were not randomized because they did not have an intervention that required randomization. Statistics were calculated using Graphpad Prism v9.0.1 software or RStudio 4.0.2 unless described otherwise in the Methods; statistical details can be found in the legends for each figure. The box for all box and whisker plots represent the 25th to 75th percentiles for minima and maxima, the line in the middle of the box represents the median, and the whiskers represent minimum and maximum values. Adobe Illustrator v26.3.1 was used to create the schematics and figures.

### Reporting summary

Further information on research design is available in the Nature Portfolio Reporting Summary linked to this article.

## Online content

Any methods, additional references, Nature Portfolio reporting summaries, source data, extended data, supplementary information, acknowledgements, peer review information; details of author contributions and competing interests; and statements of data and code availability are available at 10.1038/s41586-024-07812-3.

## Supplementary information

Supplementary Figures Supplementary Fig. 1 contains the uncropped Western blots from Extended Data Figs 8m and 9l and Supplementary Fig. 2 shows the FACS plots exemplifying the gating strategy used to sort dsRed positive cells.

Reporting Summary

Supplementary Table 1 Clinical information of ^13^C-nutrient-infused patients.

Supplementary Table 2 RNA sequencing for indicated patient tissues. Gene expression is shown as log_2_(counts per million (CPM)). Genes with 0 values for more than 75% of samples were removed.

Supplementary Table 3 Metabolomics of patient tissues

Supplementary Table 4 Isotopologue data from [U-^13^C]glucose-infused patients: 4.1 contains isotopologue data from [U-^13^C]glucose-infused patient tissues; 4.2 contains isotopologue data from [U-^13^C]glucose-infused patient plasma and 4.3 contains plasma glucose m+6 at time of tissue resection.

Supplementary Table 5 Isotopologue data from [1,2-^13^C]acetate-infused patient tissues.

Supplementary Table 6 Isotopologue data from [U-^13^C]glutamine-infused patients. Table 6.1 contains isotopologue data from [U-^13^C]glutamine-infused patient tissues. Table 6.2 contains isotopologue data from [U-^13^C]glutamine-infused patient plasma.

Supplementary Data Source data for all images: a zipped file divided into three folders for original source data images for Ki67 images, mouse images, and immunofluorescence images. Subfolders are labeled with the corresponding Figure number in which the image appears.

## Source data

Source Data Fig. 1

Source Data Fig. 2

Source Data Fig. 3

Source Data Fig. 4

Source Data Fig. 5

Source Data Extended Data Fig. 1

Source Data Extended Data Fig. 2

Source Data Extended Data Fig. 3

Source Data Extended Data Fig. 4

Source Data Extended Data Fig. 5

Source Data Extended Data Fig. 6

Source Data Extended Data Fig. 7

Source Data Extended Data Fig. 8

Source Data Extended Data Fig. 9

Source Data Extended Data Fig. 10

## Data Availability

The Human Metabolome Database can be accessed at https://hmdb.ca/. The mSigDB database can be accessed at https://www.gsea-msigdb.org/gsea/msigdb/. Supplementary table 3 from Hakimi et al.^14^ has the metabolomics data associated with their publication and can be downloaded from: https://www.ncbi.nlm.nih.gov/pmc/articles/PMC4809063/#SD2. Supplementary table 1 from Chen et al.^41^ was used for NSCLC cell line labelling data and can be found at: https://www.ncbi.nlm.nih.gov/pmc/articles/PMC6898782/. TCGA KIRC data can be accessed at https://portal.gdc.cancer.gov/projects/TCGA-KIRC. No original code was reported in this paper. Clinical information, analysed RNA sequencing data and isotopologue data are included as supplementary tables. Raw sequencing files are deposited on the Gene Expression Omnibus (GSE251905). Raw metabolomics files are available at the NIH Common Fund’s National Metabolomics Data Repository website, the Metabolomics Workbench (https://www.metabolomicsworkbench.org), where it has been assigned project ID PR001954. The data can be accessed directly via its project (10.21228/M84X6Q)^67^. Restrictions are in place in accordance with the IRB for providing patient samples. All other requests should be directed to the corresponding author (R.J.D.). Source data are provided with this paper.
