# Supplementary material for: Mitochondrial complex I promotes kidney cancer metastasis
Source: Nature. 2024 Aug 14;633(8031):923–31. doi: 10.1038/s41586-024-07812-3 (PMC11424252; doi:10.1038/s41586-024-07812-3)

nature portfolio

https://doi.org/10.1038/s41586-024-07812-3

Supplementary information

Mitochondrial complex I promotes kidney cancer metastasis

In the format provided by the
authors and unedited

Supplementary Figure 1: Uncropped Western blots from Extended Data Fig. 8m and Extended Data Fig. 9l. FLAG and β-actin were probed on the same membrane.

FLAG:

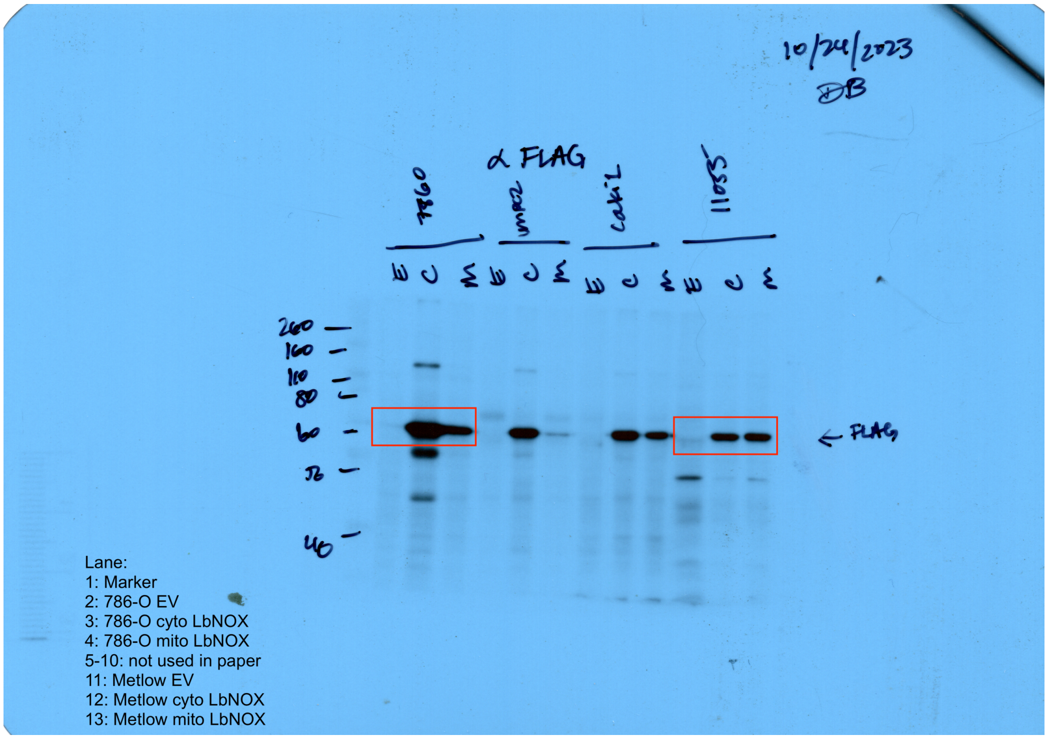

β-actin:

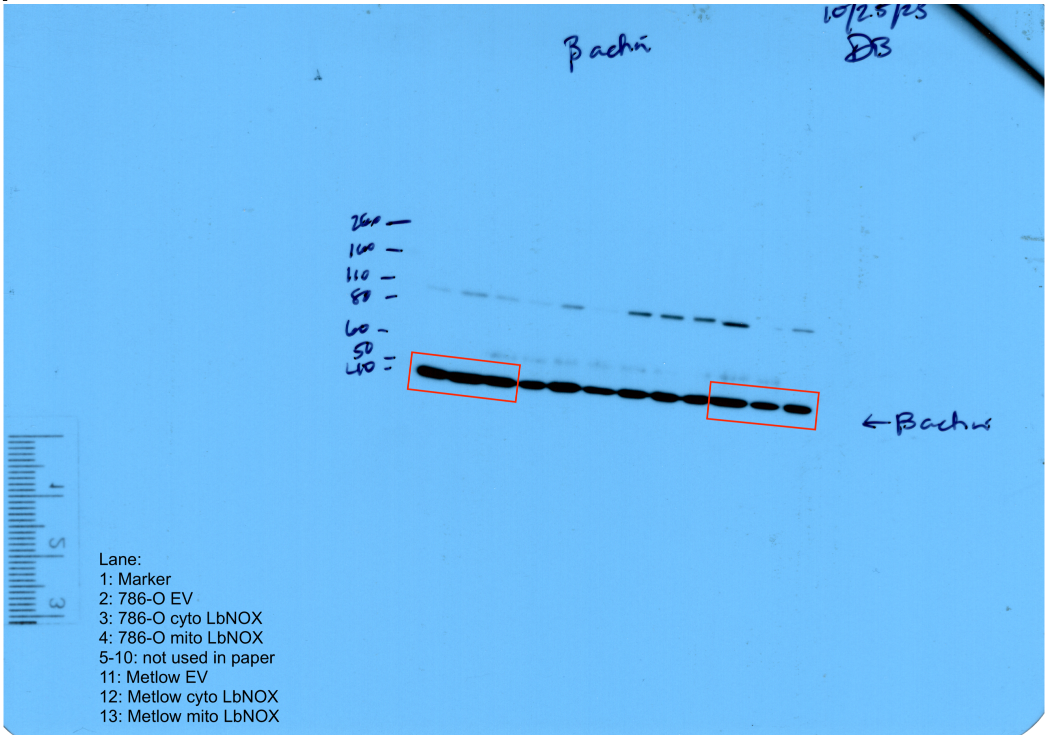

Supplementary Figure 2: FACS plots exemplifying the gating strategy used to sort dsRed positive cells.

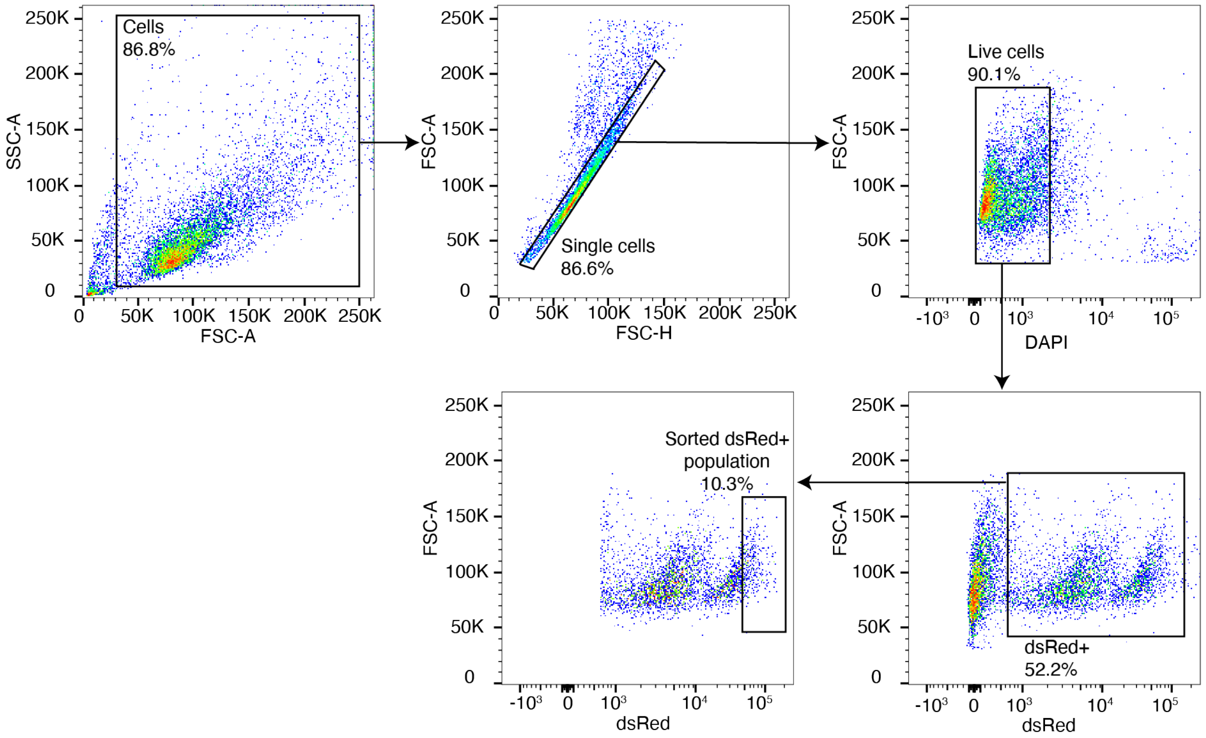

Supplement: Supplementary file 1 — Supplementary Fig. 1 contains the uncropped Western blots from Extended Data Figs 8m and 9l and Supplementary Fig. 2 shows the FACS plots exemplifying the gating strategy used to sort dsRed positive cells. [file 41586_2024_7812_MOESM1_ESM.pdf]
