# Supplementary material for: Mitochondrial complex I promotes kidney cancer metastasis
Source: Nature. 2024 Aug 14;633(8031):923–31. doi: 10.1038/s41586-024-07812-3 (PMC11424252; doi:10.1038/s41586-024-07812-3)
Supplement: Supplementary file 2 — Reporting Summary [file 41586_2024_7812_MOESM2_ESM.pdf]

Corresponding author(s): Ralph J. DeBerardinis, M.D., Ph.D.

Last updated by author(s): 06/18/2024

# Reporting Summary

Nature Portfolio wishes to improve the reproducibility of the work that we publish. This form provides structure for consistency and transparency in reporting. For further information on Nature Portfolio policies, see our Editorial Policies and the Editorial Policy Checklist.

Please do not complete any field with "not applicable" or n/a. Refer to the help text for what text to use if an item is not relevant to your study.
For final submission: please carefully check your responses for accuracy; you will not be able to make changes later.

## Statistics

For all statistical analyses, confirm that the following items are present in the figure legend, table legend, main text, or Methods section.

| n/a | Confirmed | |
|---|---|---|
| ☐ | ☒ | The exact sample size (*n*) for each experimental group/condition, given as a discrete number and unit of measurement |
| ☐ | ☒ | A statement on whether measurements were taken from distinct samples or whether the same sample was measured repeatedly |
| ☐ | ☒ | The statistical test(s) used AND whether they are one- or two-sided<br>*Only common tests should be described solely by name; describe more complex techniques in the Methods section.* |
| ☐ | ☒ | A description of all covariates tested |
| ☐ | ☒ | A description of any assumptions or corrections, such as tests of normality and adjustment for multiple comparisons |
| ☐ | ☒ | A full description of the statistical parameters including central tendency (e.g. means) or other basic estimates (e.g. regression coefficient) AND variation (e.g. standard deviation) or associated estimates of uncertainty (e.g. confidence intervals) |
| ☐ | ☒ | For null hypothesis testing, the test statistic (e.g. $F$, $t$, $r$) with confidence intervals, effect sizes, degrees of freedom and $P$ value noted<br>*Give P values as exact values whenever suitable.* |
| ☒ | ☐ | For Bayesian analysis, information on the choice of priors and Markov chain Monte Carlo settings |
| ☒ | ☐ | For hierarchical and complex designs, identification of the appropriate level for tests and full reporting of outcomes |
| ☐ | ☒ | Estimates of effect sizes (e.g. Cohen's *d*, Pearson's *r*), indicating how they were calculated |

*Our web collection on statistics for biologists contains articles on many of the points above.*

## Software and code

Policy information about availability of computer code

| | |
|---|---|
| Data collection | GC/MS data was collected using an Agilent 7890A gas chromatograph coupled to an Agilent 5975C. LC/MS/MS data were collected using an Agilent Q-TOF, Thermo Fusion Lumos, or AB Sciex 6500 QTRAP. Respiration measurements were made using a Seahorse XFe96 Analyzer. qPCR data was collected using a Bio-Rad CFX384 Touch Real-Time PCR Detection machine. Flow cytometry data was collected using BD FACS Aria of FACSymphony 6 (BD Biosciences). RNA sequencing data was collected using an Illumina NextSeq500. Bioluminescence data was collected using an IVIS Imaging System 200 Series or a Spectral Imaging AMI-HTX. Magnetic resonance images were taken using a BrukerBioSpin 7T Bruker Biospec. Immunofluorescence was visualized using a Zeiss LSM 880 Confocal Laser Scanning Microscope. Ki67 staining was visualized using a Zeiss LSM780 confocal microscope. tdTomato positive metastatic lesions and and brightfield images were acquired using a Leica MZ12s stereo microscop |
| Data analysis | GraphPad Prism V9.0.1 or R 4.0.2 was used for data analysis. Flow cytometry data was analyzed using BD FACSDiva 8.0 (BD Biosciences) and FlowJo V10 (Treestar). GC/MS data was analyzed using Agilent ChemStation E02.0.2.1431 or el Maven (https://www.elucidata.io/el-maven). LC/MS/MS data were analyzed using Sciex MultiQuant V2.1.1, Thermo Fisher Trace Finder 4.1, or Agilent Masshunter Profinder 8.0. Bioluminescence data were analyzed using Living Image software V4.3.1 or Aura Imaging software V4.0. Immunofluorescence, brightfield, and Ki67 images were quantified with ImageJ or LAS v4.13 software (Leica Microsystems). Adobe Illustrator V26.3.1 was used to create paper schematics and figures.<br><br>For RNA sequencing: Sequencing reads were aligned to the human reference genome (hg19) by STAR 2.7.3.a with default parameters in the 2-pass mod. Counts for each gene were generated using htseq-count v0.6.1. DEGs were identified by DESeq2 v1.14.1. Ends of sequences were trimmed with remaining adapter or quality scores <25. Sequences less than 35bp after trimming were removed. The trimmed Fastq files were aligned to the GRCh38 using HiSAT2 and duplicates were marked with SAMBAMBA. Features (genes, transcripts, and exons) were counted using featureCounts. Differential expression analysis was performed using EdgeR and DESeq. All code has been previously published and is |

referenced appropriately in the Methods section of the paper.

The ESTIMATE R package was used to derive stromal and immune scores and a tumour purity ESTIMATE score from RNA sequencing data. The ESTIMATE R package is previously published and accessible from Reference 57. The MSigDB database was used to define OxPhos genes for the OxPhos score. The HMDB database was used to map metabolite names to their identifiers.

The CRISPR screen was analyzed as follows: Bowtie63 was used to obtain raw read-counts with 1 mismatch allowance, thus taking the best-matching sgRNA per read. Bayesian Analysis of Gene EssentiaLity 2 (BAGEL2) software was then used to calculate normalized read-counts, and log2 fold change was obtained by comparing the reference timepoint of the corresponding cell line. Next, genes were determined as vulnerabilities by applying the standard BAGEL2 pipeline and excluding core-essential genes. The top 500 gene vulnerabilities ranked by BAGEL score were used as an input for Enrichment Pathway Analysis using the WEB-based GEne SeT AnaLysis Toolkit (WebGestalt).

For manuscripts utilizing custom algorithms or software that are central to the research but not yet described in published literature, software must be made available to editors and reviewers. We strongly encourage code deposition in a community repository (e.g. GitHub). See the Nature Portfolio guidelines for submitting code & software for further information.

# Data

Policy information about availability of data

All manuscripts must include a data availability statement. This statement should provide the following information, where applicable:

- Accession codes, unique identifiers, or web links for publicly available datasets
- A description of any restrictions on data availability
- For clinical datasets or third party data, please ensure that the statement adheres to our policy

The Human Metabolome Database (HMDB) can be accessed at https://hmdb.ca/. The mSigDB database can be accessed at https://www.gsea-msigdb.org/gsea/msigdb/. Table S3 from Hakimi et al14 has the metabolomics data associated with their publication, and can be downloaded here: https://www.ncbi.nlm.nih.gov/pmc/articles/PMC4809063/#SD2. Table S1 from Chen et al41 was used for NSCLC cell line labeling data and can be found here: https://www.ncbi.nlm.nih.gov/pmc/articles/PMC6898782/. TCGA KIRC data can be accessed at https://portal.gdc.cancer.gov/projects/TCGA-KIRC.

No original code was reported in this paper. Source data are provided with this paper. Clinical information, analyzed RNA-sequencing data, and isotopologue data are included as Supplementary Data Tables. Raw sequencing files are deposited on GEO (GSE251905, https://www.ncbi.nlm.nih.gov/geo/query/acc.cgi?acc=GSE251905). Raw metabolomics files are available at the NIH Common Fund's National Metabolomics Data Repository (NMDR) website, the Metabolomics Workbench, https://www.metabolomicsworkbench.org where it has been assigned Project ID PR001954. The data can be accessed directly via its Project DOI: http://dx.doi.org/10.21228/M84X6Q67. Restrictions are in place in accordance with the Institutional Review Board (IRB) for providing patient samples. All other requests should be directed to the corresponding author, Ralph DeBerardinis, M.D., Ph.D.

# Research involving human participants, their data, or biological material

Policy information about studies with human participants or human data. See also policy information about sex, gender (identity/presentation), and sexual orientation and race, ethnicity and racism.

| | |
|---|---|
| Reporting on sex and gender | No exclusion criteria related to sex and gender were present for the study. |
| Reporting on race, ethnicity, or other socially relevant groupings | Not applicable. |
| Population characteristics | Population characteristics are included in Extended Data Table 1. |
| Recruitment | For STU2019-1061 that recruited most patients reported in this paper, patients were deemed eligible if they met the following criteria: (1) Patients must have radiographic evidence of known or probable kidney or urothelial cancer requiring surgical biopsy or excision. (2) Age ≥ 18 years (3) Subjects of all races and ethnic origins (4) The willingness to sign and ability to understand a written informed consent. (5) Patients participating in other clinical trials are eligible, and were evaluated on a case by case basis by Dr. Vitaly Margulis, MD. Exclusion criteria were the following: (1) Uncontrolled or poorly controlled diabetes for patients receiving a 13C glucose infusion (2) Pregnant or breastfeeding (3) Not a surgical candidate. For STU062010-157, patients were deemed eligible if they met the following criteria: (1) Male or female, any age and any racial or ethnic group (2) Spanish-speaking patients will be eligible (3) Pre-operative brain MR imaging suggestive of a brain tumor or pre-operative imaging showing a tumor or biopsy of a tumor mass prior to planned resection of the mass (4) Patient able and willing to provide informed consent (5) Karnofsky Performance status > 70% (6) Negative serum pregnancy test or child bearing potential terminated by surgery, radiation, menopause or current use of two approved methods of birth control. Exclusion criteria were the following: (1) Karnofsky Performance status < 70% (2) NYHA class III and IV congestive heart failure (3) Psychiatric or addictive disorders that preclude obtaining informed consent (4) Unstable angina (5) Pregnant or lactating women. For STU052012-065, patients were deemed eligible if they met the following criteria: (1) Patients must have known or probable malignant lesions requiring surgical biopsy or excision. (2) Subjects of all races and ethnic origins over 18 years of age. Exclusion criteria were the following: (1) Not a surgical candidate (2) Poorly controlled diabetes. Almost all patients undergoing nephrectomy were evaluated by the first author for initial eligibility and subsequently evaluated for inclusion criteria by the physicians noted below. As such, self-selection bias was not introduced during patient selection. Patients who elected to participate in the study were given specific tracers based on Investigator experience, clinical safety, and tracer cost and availability. We do not believe that tracer allocation or patient selection introduced undue bias or impacted results.<br><br>All patients that met eligibility criteria were contacted by a member of the patient's care team who mentioned the study to |

the patient either in person or over the phone. If the patient was interested in participating in the study, the patient's chart was reviewed for eligibility by an attending physician and the first author. If eligible, the consent forms and the study were reviewed in detail with the patient, forms were given to the patient for review, and a signature was obtained for consent. It was emphasized to all patients that they received no benefit to participating in the study and their care would not be affected in any way by declining participation. A copy of the consent form was given to the patient for their records. The written informed consent form is signed and personally dated by the subject and by the person who conducted the informed consent discussion. If the patient is Spanish speaking, then a copy of the consent form was given to the patient in Spanish, and the consent form and study was reviewed in detail with a Spanish speaking translator. Each consent form includes all the relevant elements currently required by FDA Regulations and local or state regulations. All subjects on STU2019-1061 were evaluated by Vitaly Margulis, M.D. Participating patients with kidney cancer on STU062010-157 were evaluated by Kevin Courtney, M.D., Ph.D. Two patients with lung metastases were recruited on study STU052012-065 and were evaluated by Kemp Kernstine, M.D., Ph.D. If the patient was a patient of Dr. Margulis, Dr. Courtney, or Dr. Kernstine, then the patient was informed that their physician was an investigator of the study, and reviewed conflict of interest with the patients that choose to participate in the study. Drs. Margulis, Courtney, and Kernstine were careful to describe all available trials to potential patients, to answer questions, and to help the patients make participation decisions in a non-directive manner. The patient was given a copy of the informed consent document to take home and was encouraged to discuss participation in the study with family members and friends before deciding on participation.

Ethics oversight

The Institutional Review Board (IRB) at the University of Texas Southwestern Medical Center monitored and approved all conducted human subjects research. Study protocols are subjected to annual continuing reviews by the IRB.

Note that full information on the approval of the study protocol must also be provided in the manuscript.

# Field-specific reporting

Please select the one below that is the best fit for your research. If you are not sure, read the appropriate sections before making your selection.

☒ Life sciences ☐ Behavioural & social sciences ☐ Ecological, evolutionary & environmental sciences

For a reference copy of the document with all sections, see nature.com/documents/nr-reporting-summary-flat.pdf

# Life sciences study design

All studies must disclose on these points even when the disclosure is negative.

Sample size

Sample size calculations were performed prior to clinical trials, but the individual trials have not finished accruing patients and completed study. As such, the clinical trial sample size calculations are not applicable for this paper. Accrual of patients per subtype and/or metastastic status were based on surgical patient recruitment and volume in clinic, and reflect incidence of cancer subtypes. Inclusion and exclusion criteria are outlined in the 'Human research participants' section. We aimed for a minimum of 3 patients per kidney cancer subtype. For all other experiments, no pre-determined sample size calculations were performed. Sample sizes were based on authors' experiences with similar experiments in the past.

Data exclusions

For metastasis experiments, mice were excluded if the BLI signal was only in the mouse tail, indicating an unsuccessful intravenous injection. For patient tissue isotopologue analysis by GC/MS, peak areas that could not be confidently assessed as the identified metabolite were not included. This applies to Extended Data Tables 4, 5, and 6. For mitochondrial respiration assays, patient mitochondrial samples were excluded if they did not demonstrate an intact outer mitochondrial membrane during Proteinase K assays (described in Methods).

Replication

For all mouse experiments, two independent cohorts were injected and monitored in independent experiments. For all in vitro assays, at least three biological replicates were performed to allow for adequate statistical analyses. Each biological replicate was defined as an independent culture of cells. All replication efforts for mouse and in vitro experiments were successful. Patient samples was collected from different patients on their day of surgery over the course of four years. Studies on human samples were not replicated from individual patients, because each tumour could only be sampled at a single point in time.

Randomization

Most experiments were not randomized because they did not have an intervention that required randomization. Patients were recruited to a non-therapeutic trial that did not require randomization, and could not be blinded for safety. Investigators needed to know what tracer was being given to monitor for adverse effects. Mice were randomly allocated for injection and/or treatment.

Blinding

During sample processing and analysis for mass spectrometry analysis, qPCR, and RNA sequencing, samples were given a de-identified alpha numeric code. After analysis was completed, samples were unblinded and graphed. All mice were given a numerical identifier, and tumour measurements were recorded according to the numerical identifier and unblinded at the end of the study. Mouse experiments requiring pharmacological treatments were not blinded so that mice could be monitored for toxicities. All other experiments were not blinded because there was no observation or subject interaction that was susceptible to observer bias.

# Reporting for specific materials, systems and methods

We require information from authors about some types of materials, experimental systems and methods used in many studies. Here, indicate whether each material, system or method listed is relevant to your study. If you are not sure if a list item applies to your research, read the appropriate section before selecting a response.

## Materials & experimental systems

| n/a | Involved in the study |
|---|---|
| | ☒ Antibodies |
| | ☒ Eukaryotic cell lines |
| ☒ | Palaeontology and archaeology |
| | ☒ Animals and other organisms |
| | ☒ Clinical data |
| ☒ | Dual use research of concern |
| ☒ | Plants |

## Methods

| n/a | Involved in the study |
|---|---|
| ☒ | ChIP-seq |
| | ☒ Flow cytometry |
| ☒ | MRI-based neuroimaging |

# Antibodies

| | |
|---|---|
| Antibodies used | anti-FLAG M2, Sigma Aldrich, Cat. No. F1804, 1:2000 dilution (WB) and 1:200 dilution (IF)<br>anti-β-actin, Cell Signaling Technology, Cat. No. 8457S, 1:2000 dilution<br>anti-HSP60, Cell Signaling Technology, Cat. No. 12165S, 1:2000 dilution (WB) and 1:500 dilution (IF)<br>anti-Tom20, Proteintech, Cat. No. 11802, 1:2000 dilution<br>anti-Mouse IgG, HRP linked, Cell Signaling Technology, Cat. No. 7076S, 1:2000 dilution<br>anti-Rabbit IgG, HRP linked, Cell Signaling Technology, Cat. No. 7074S, 1:2000 dilution<br>Donkey anti-Mouse IgG (H+L) Highly Cross-Adsorbed Secondary Antibody, Alexa Fluor™ 555, Thermo Fisher, Cat. No. A31570, 1:500 dilution<br>Alexa Fluor® 488 AffiniPure™ Goat Anti-Rabbit IgG (H+L), Jackson ImmunoResearch Laboratories, Cat. No. 111-545-144, 1:500 dilution |
| Validation | All antibodies are commercially available and were not independently validated by the authors of the study. |

# Eukaryotic cell lines

Policy information about cell lines and Sex and Gender in Research

| | |
|---|---|
| Cell line source(s) | 786-O, Caki-1, and primary renal proximal tubule epithelial cells (RPTEC) were purchased from the American Type Culture Collection (ATCC, Cat No. CRL-1932, HTB-46, PCS-400-010, respectively). HEK293FT cells were purchased from Thermo Fisher (Cat No. 70007). Metlow, Methigh, Methigh-50, and Methigh-26 were established from somatic mosaic genetically engineered mouse models (SM-GEMM) of kidney cancer reported by Perelli et al, 2023, and are reported in this paper. |
| Authentication | 786-O, Caki-1, and primary renal proximal tubule epithelial cells (RPTEC) cells were authenticated by the American Type Culture Collection (ATCC) using short tandem repeat (STR) profiling. HEK293FT cells were not authenticated after purchase from Thermo Fisher. |
| Mycoplasma contamination | All cell lines were confirmed to be mycoplasma free using a commercial kit (Bulldog Bio, Cat. No. 2523348) at least once a month. |
| Commonly misidentified lines<br>(See ICLAC register) | No commonly misidentified lines were used in this paper. |

# Animals and other research organisms

Policy information about studies involving animals; ARRIVE guidelines recommended for reporting animal research, and Sex and Gender in Research

| | |
|---|---|
| Laboratory animals | Male and female NOD.CB17-Prkdc(scid) Il2rg(tm1Wjl)/SzJ (NSG) mice aged four to eight weeks old and six to nine week old NU-Foxn1nu (nude) mice were used in this study. Mice were purchased from Jackson Laboratories. NSG mice were housed in a pathogen free environment at a temperature range of 68-79F and a humidity range of 30-70%. Mice were kept on a 12 hour dark, 12 hour light cycle and fed a standard chow diet ad libitium. Male and female Nude mice were housed in a pathogen free environment at a temperature range of 65-74F with a humidity range of 50-60% Mice were kept on a 12 hour dark, 12 hour light cycle and fed a standard chow diet ad libitium. |
| Wild animals | No wild animals were used in this study. |
| Reporting on sex | Both male and female mice were used for this study. Data was not analyzed for sex specific differences as the goal was to assay metastasis. |
| Field-collected samples | No filed collected samples were used in this study. |
| Ethics oversight | The University of Texas Southwestern Medical Center Institutional Animal Use and Care Committee (IACUC) and the University of Texas MD Anderson Cancer Center IACUC approved and provided guidance and oversight on animal study protocols. |

Note that full information on the approval of the study protocol must also be provided in the manuscript.

## Clinical data

Policy information about clinical studies

All manuscripts should comply with the ICMJE guidelines for publication of clinical research and a completed CONSORT checklist must be included with all submissions.

| | |
|---|---|
| Clinical trial registration | NCT04623502 and NCT01668082 and NCT02095808 |
| Study protocol | Trial information is provided on clinicaltrials.gov |
| Data collection | All patients were recruited at the University of Texas Southwestern Medical Center. From December 2018-August 2019, patients were enrolled on protocol STU062010-157. From September 2019-present, patients are enrolled on protocol STU2019-1061. Two patients were enrolled on protocol STU052012-065. |
| Outcomes | This paper does not report final results of therapeutic interventional trials or phase II and phase III randomized controlled trials. Outcome measurements are listed in the clinicaltrials.gov record, but are not applicable to this study because they have not finished accrual and all trials are non-therapeutic trials |

## Plants

| | |
|---|---|
| Seed stocks | N/A |
| Novel plant genotypes | N/A |
| Authentication | N/A |

## Flow Cytometry

### Plots

Confirm that:

☒ The axis labels state the marker and fluorochrome used (e.g. CD4-FITC).

☒ The axis scales are clearly visible. Include numbers along axes only for bottom left plot of group (a 'group' is an analysis of identical markers).

☒ All plots are contour plots with outliers or pseudocolor plots.

☒ A numerical value for number of cells or percentage (with statistics) is provided.

### Methodology

| | |
|---|---|
| Sample preparation | 786-O and Caki-1 cells expressing dsRED were trypsinized and filtered through a 40 µm cell strainer to obtain a single cell suspension. |
| Instrument | BD FACS Aria, BD FACSymphony 6 |
| Software | BD FACSDiva 8.0, FlowJo V10 |
| Cell population abundance | To eliminate dead cells from sorts and analyses, cells were stained with 4’,6-diamidino-2-phenylindole (DAPI). dsRED positive cells were then sorted and collected. |
| Gating strategy | Cells were gated to exclude dead cells, cell debris, and doublets based on FSC/SSC, then gated on live cells (DAPI negative cells). The top 10% of dsRED positive cells were sorted, collected, and used for experiments. Example FACS plots exemplifying the gating strategy are provided in Supplementary Figure 2. Data was analyzed using BD FACSDiva 8.0 and FlowJo V10. |

☒ Tick this box to confirm that a figure exemplifying the gating strategy is provided in the Supplementary Information.

This checklist template is licensed under a Creative Commons Attribution 4.0 International License, which permits use, sharing, adaptation, distribution and reproduction in any medium or format, as long as you give appropriate credit to the original author(s) and the source, provide a link to the Creative Commons license, and indicate if changes were made. The images or other third party material in this article are included in the article's Creative Commons license, unless indicated otherwise in a credit line to the material. If material is not included in the article's Creative Commons license and your intended use is not permitted by statutory regulation or exceeds the permitted use, you will need to obtain permission directly from the copyright holder. To view a copy of this license, visit http://creativecommons.org/licenses/by/4.0/
