# Supplementary figures and images for: Mitochondrial complex I promotes kidney cancer metastasis
Source: Nature. 2024 Aug 14;633(8031):923–31. doi: 10.1038/s41586-024-07812-3 (PMC11424252; doi:10.1038/s41586-024-07812-3)

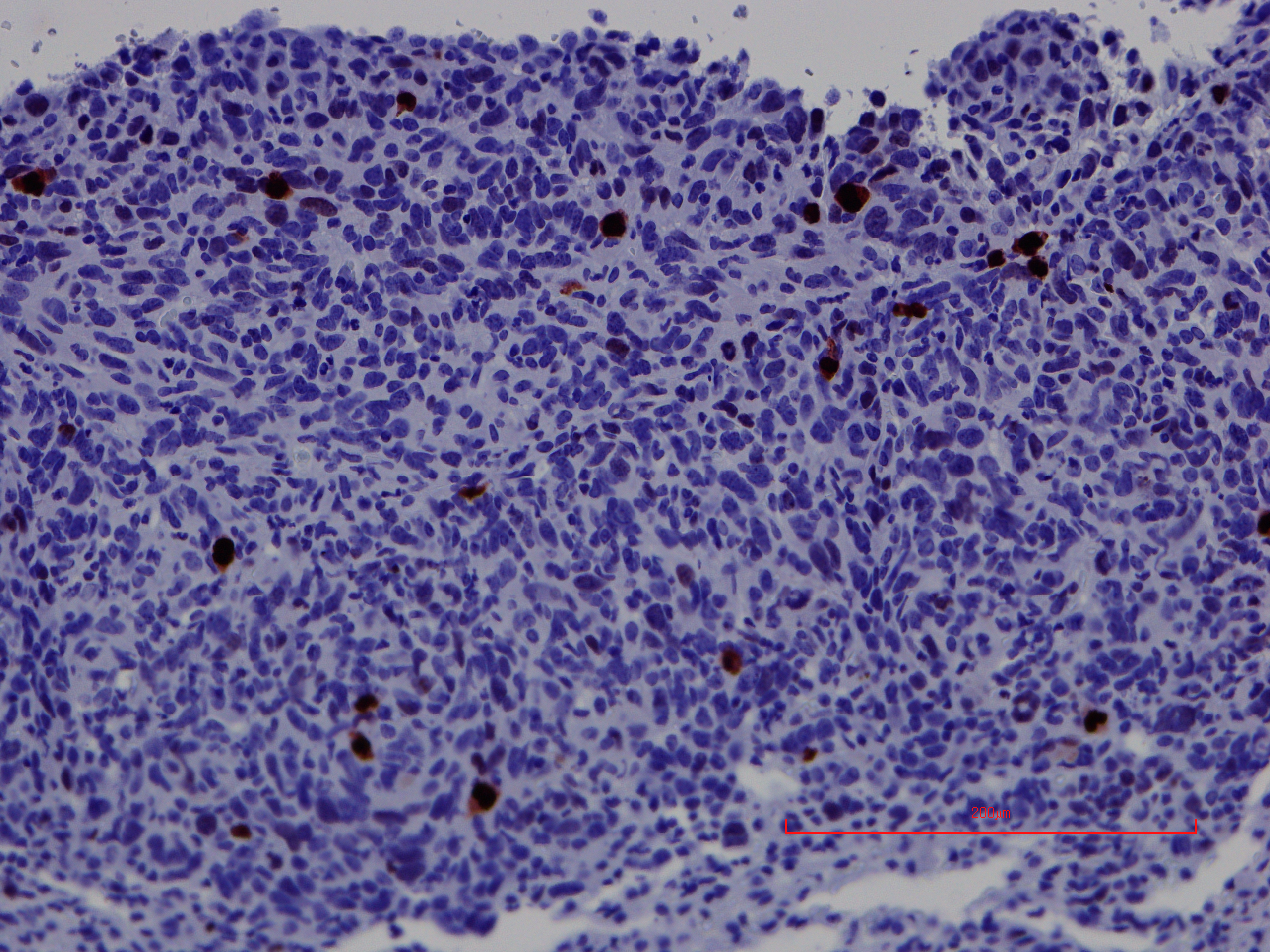

Supplement: Supplementary file 9 — Source data for all images: a zipped file divided into three folders for original source data images for Ki67 images, mouse images, and immunofluorescence images. Subfolders are labeled with the corresponding Figure number in which the image appears. [file 41586_2024_7812_MOESM9_ESM.zip › Images/Extended_Data_Fig_8g_Ki67/Methigh_Vehicle.TIF]

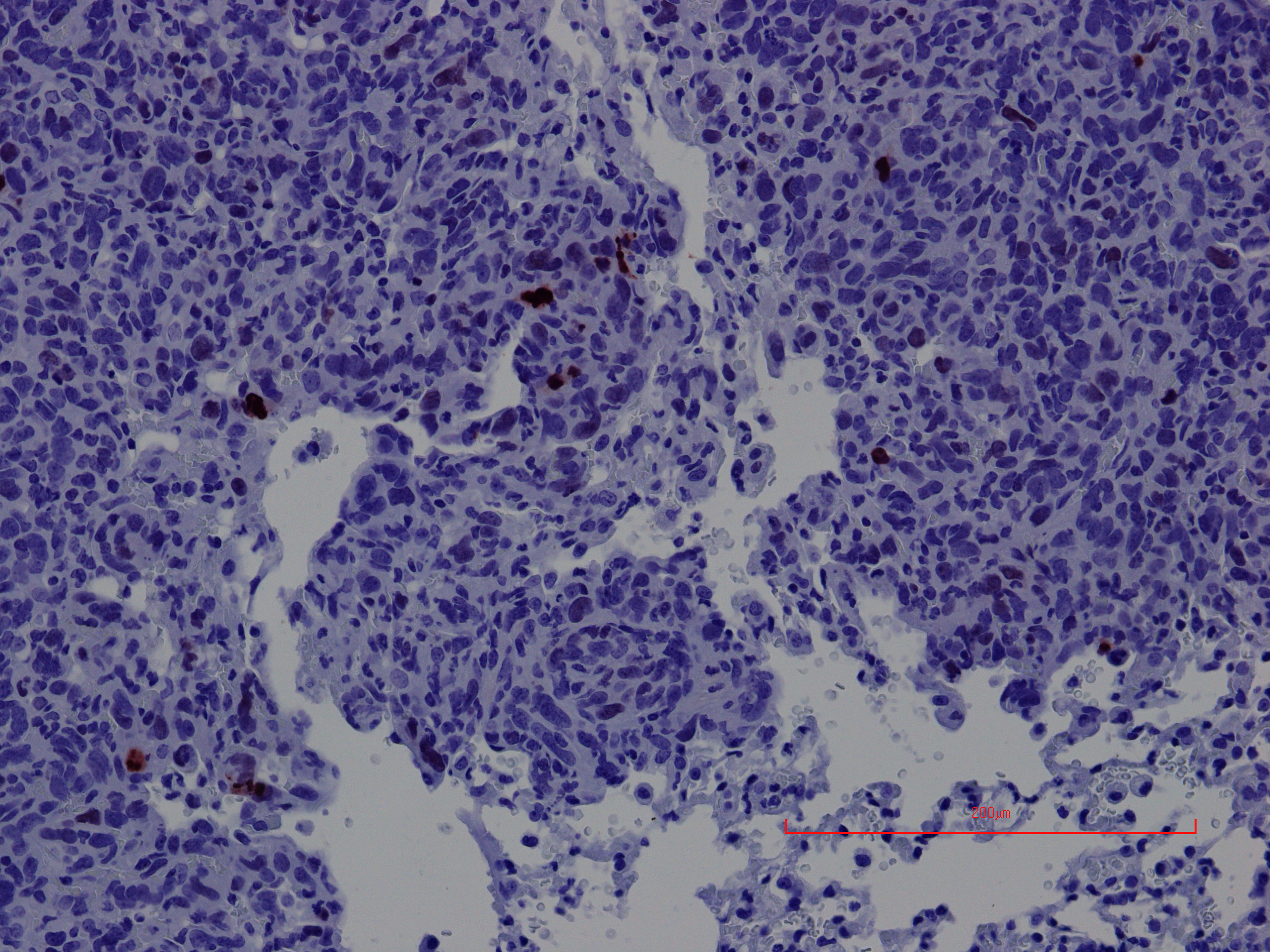

Supplement: Supplementary file 9 — Source data for all images: a zipped file divided into three folders for original source data images for Ki67 images, mouse images, and immunofluorescence images. Subfolders are labeled with the corresponding Figure number in which the image appears. [file 41586_2024_7812_MOESM9_ESM.zip › Images/Extended_Data_Fig_8g_Ki67/Methigh_IACS.TIF]

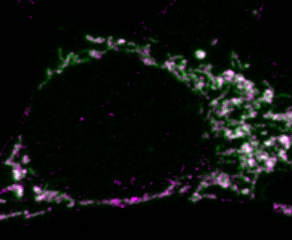

Supplement: Supplementary file 9 — Source data for all images: a zipped file divided into three folders for original source data images for Ki67 images, mouse images, and immunofluorescence images. Subfolders are labeled with the corresponding Figure number in which the image appears. [file 41586_2024_7812_MOESM9_ESM.zip › Images/Immunofluorescence/Extended_Data_Fig8n_Metlow_IF/Composite-1 (RGB).tif]

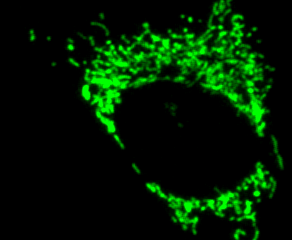

Supplement: Supplementary file 9 — Source data for all images: a zipped file divided into three folders for original source data images for Ki67 images, mouse images, and immunofluorescence images. Subfolders are labeled with the corresponding Figure number in which the image appears. [file 41586_2024_7812_MOESM9_ESM.zip › Images/Immunofluorescence/Extended_Data_Fig8n_Metlow_IF/MAX_Image 8.czi - C=1-1.tif]

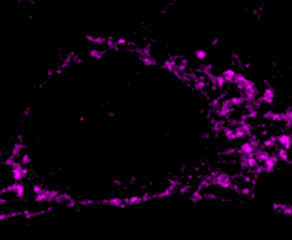

Supplement: Supplementary file 9 — Source data for all images: a zipped file divided into three folders for original source data images for Ki67 images, mouse images, and immunofluorescence images. Subfolders are labeled with the corresponding Figure number in which the image appears. [file 41586_2024_7812_MOESM9_ESM.zip › Images/Immunofluorescence/Extended_Data_Fig8n_Metlow_IF/MAX_Image 6.czi - C=0-1.tif]

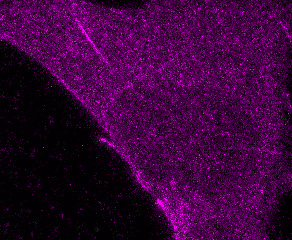

Supplement: Supplementary file 9 — Source data for all images: a zipped file divided into three folders for original source data images for Ki67 images, mouse images, and immunofluorescence images. Subfolders are labeled with the corresponding Figure number in which the image appears. [file 41586_2024_7812_MOESM9_ESM.zip › Images/Immunofluorescence/Extended_Data_Fig8n_Metlow_IF/MAX_Image 8.czi - C=0-1.tif]

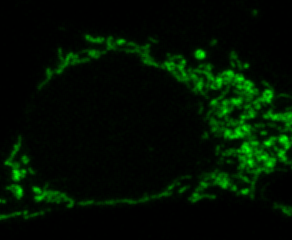

Supplement: Supplementary file 9 — Source data for all images: a zipped file divided into three folders for original source data images for Ki67 images, mouse images, and immunofluorescence images. Subfolders are labeled with the corresponding Figure number in which the image appears. [file 41586_2024_7812_MOESM9_ESM.zip › Images/Immunofluorescence/Extended_Data_Fig8n_Metlow_IF/MAX_Image 6.czi - C=1-1.tif]

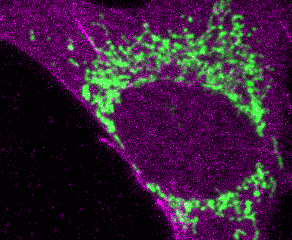

Supplement: Supplementary file 9 — Source data for all images: a zipped file divided into three folders for original source data images for Ki67 images, mouse images, and immunofluorescence images. Subfolders are labeled with the corresponding Figure number in which the image appears. [file 41586_2024_7812_MOESM9_ESM.zip › Images/Immunofluorescence/Extended_Data_Fig8n_Metlow_IF/Composite-3 (RGB).tif]

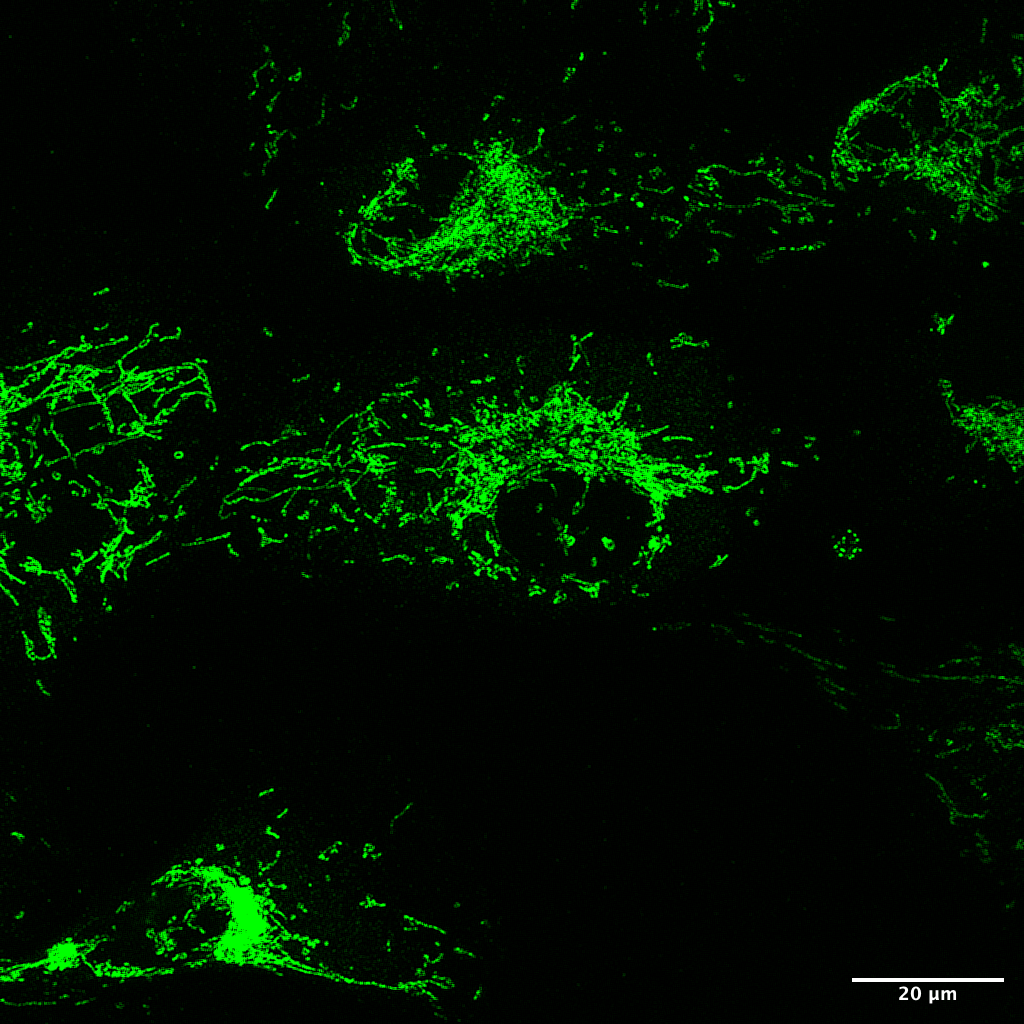

Supplement: Supplementary file 9 — Source data for all images: a zipped file divided into three folders for original source data images for Ki67 images, mouse images, and immunofluorescence images. Subfolders are labeled with the corresponding Figure number in which the image appears. [file 41586_2024_7812_MOESM9_ESM.zip › Images/Immunofluorescence/Extended_Data_Fig_9k_786-O_IF/Scale barMAX_786-O MitoLbnox2.czi - C=1-1.tif]

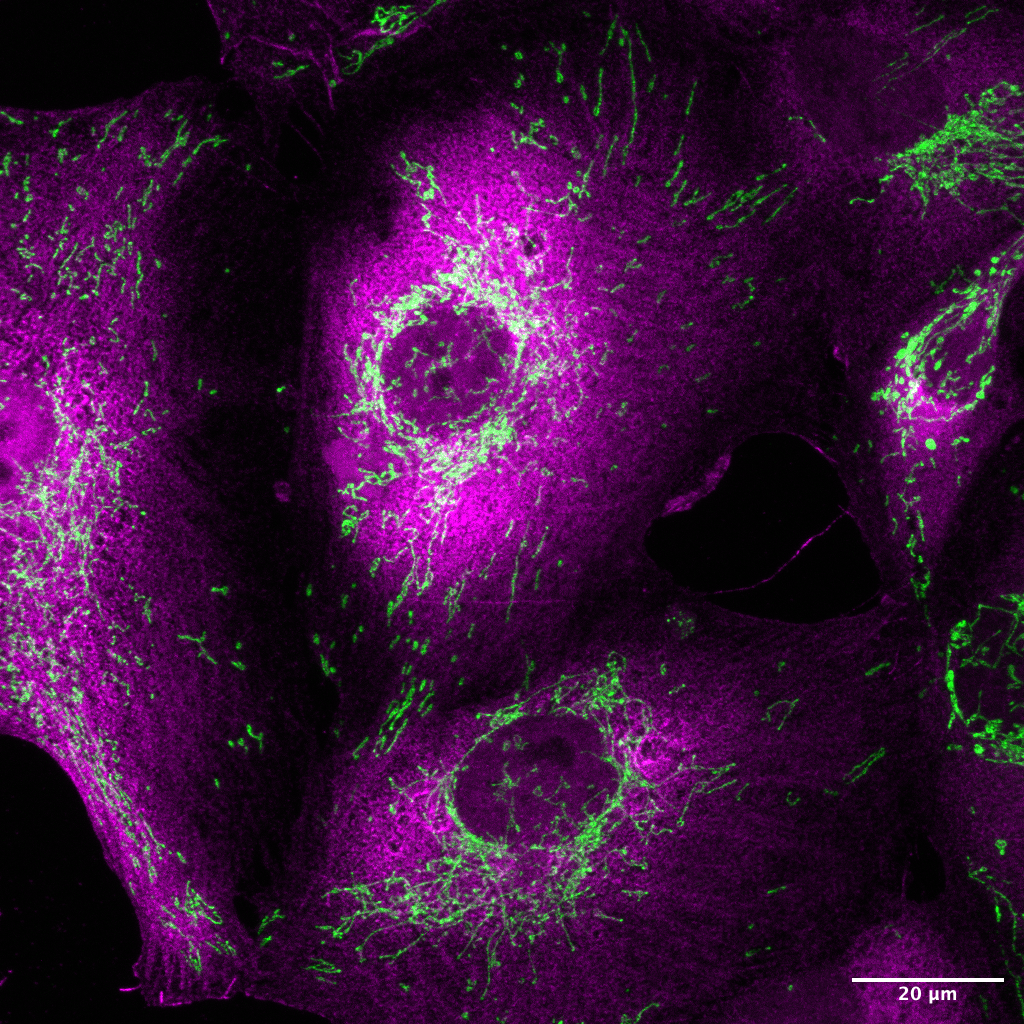

Supplement: Supplementary file 9 — Source data for all images: a zipped file divided into three folders for original source data images for Ki67 images, mouse images, and immunofluorescence images. Subfolders are labeled with the corresponding Figure number in which the image appears. [file 41586_2024_7812_MOESM9_ESM.zip › Images/Immunofluorescence/Extended_Data_Fig_9k_786-O_IF/Scale bar Composite-1.tif]

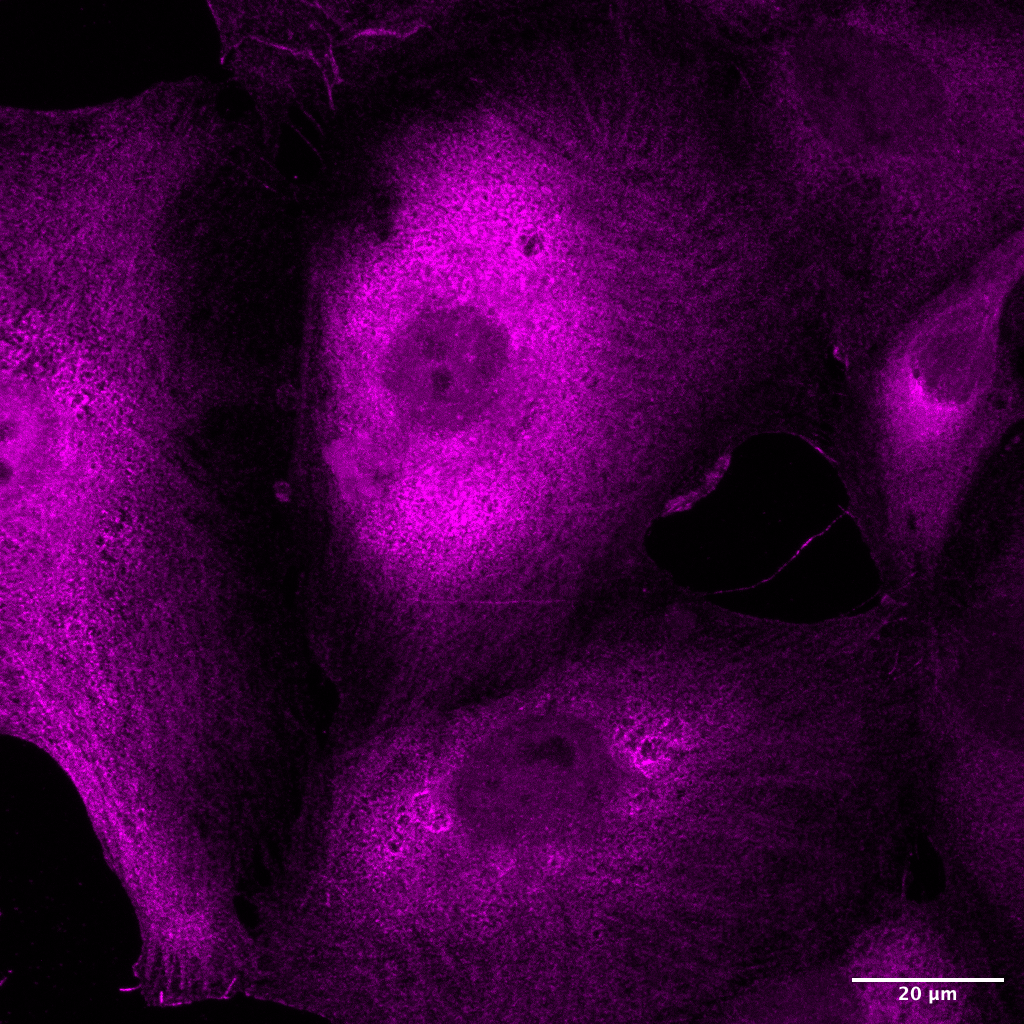

Supplement: Supplementary file 9 — Source data for all images: a zipped file divided into three folders for original source data images for Ki67 images, mouse images, and immunofluorescence images. Subfolders are labeled with the corresponding Figure number in which the image appears. [file 41586_2024_7812_MOESM9_ESM.zip › Images/Immunofluorescence/Extended_Data_Fig_9k_786-O_IF/Scale bar MAX_786-O CytoLbnox3.czi - C=0-1.tif]

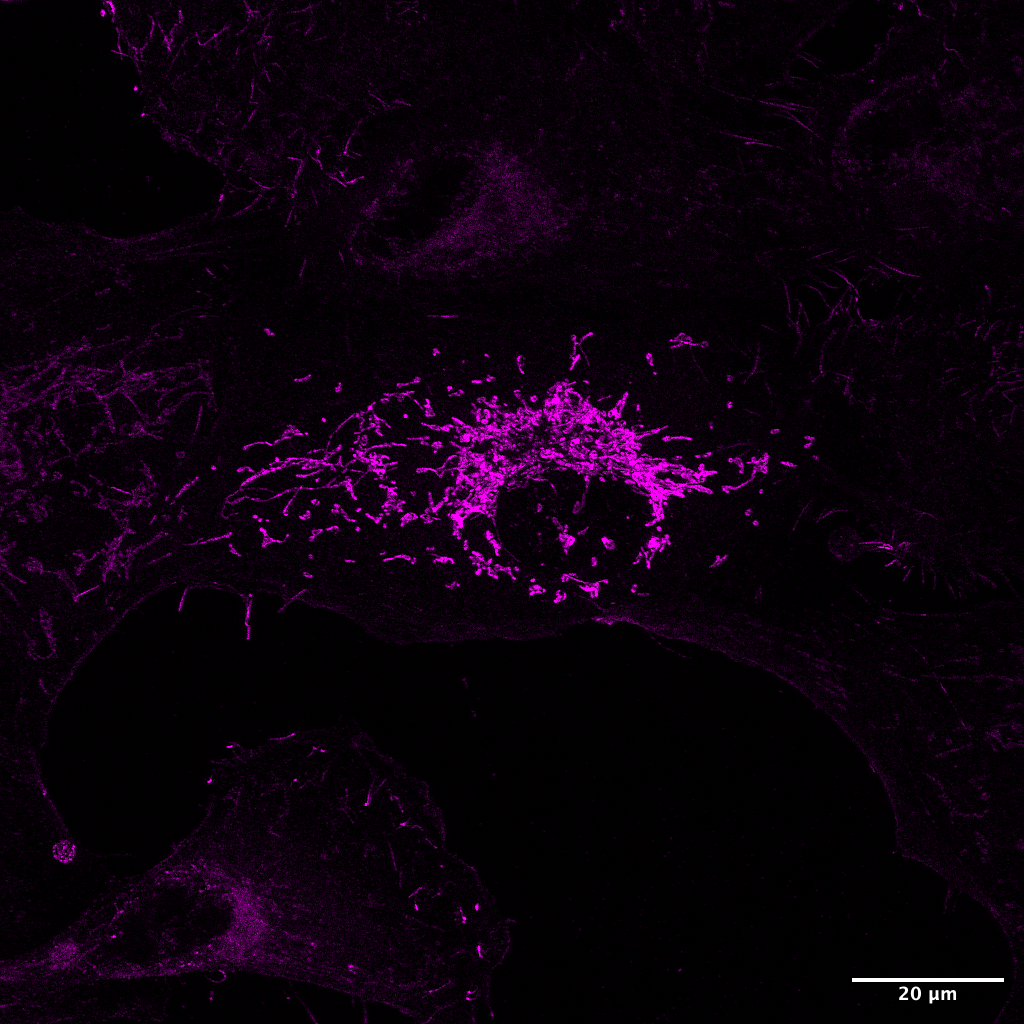

Supplement: Supplementary file 9 — Source data for all images: a zipped file divided into three folders for original source data images for Ki67 images, mouse images, and immunofluorescence images. Subfolders are labeled with the corresponding Figure number in which the image appears. [file 41586_2024_7812_MOESM9_ESM.zip › Images/Immunofluorescence/Extended_Data_Fig_9k_786-O_IF/Scale bar MAX_786-O MitoLbnox2.czi - C=0-1.tif]

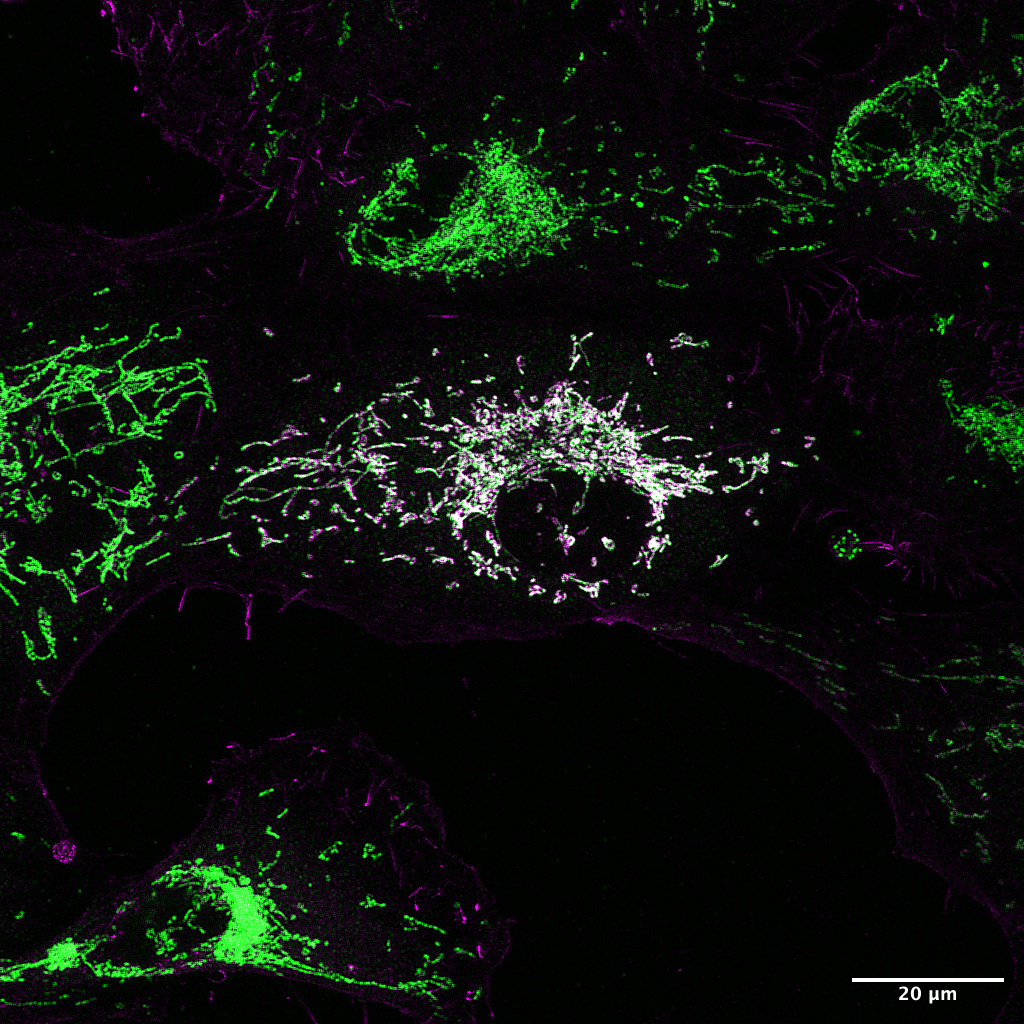

Supplement: Supplementary file 9 — Source data for all images: a zipped file divided into three folders for original source data images for Ki67 images, mouse images, and immunofluorescence images. Subfolders are labeled with the corresponding Figure number in which the image appears. [file 41586_2024_7812_MOESM9_ESM.zip › Images/Immunofluorescence/Extended_Data_Fig_9k_786-O_IF/Scale bar Composite-1[1].tif]

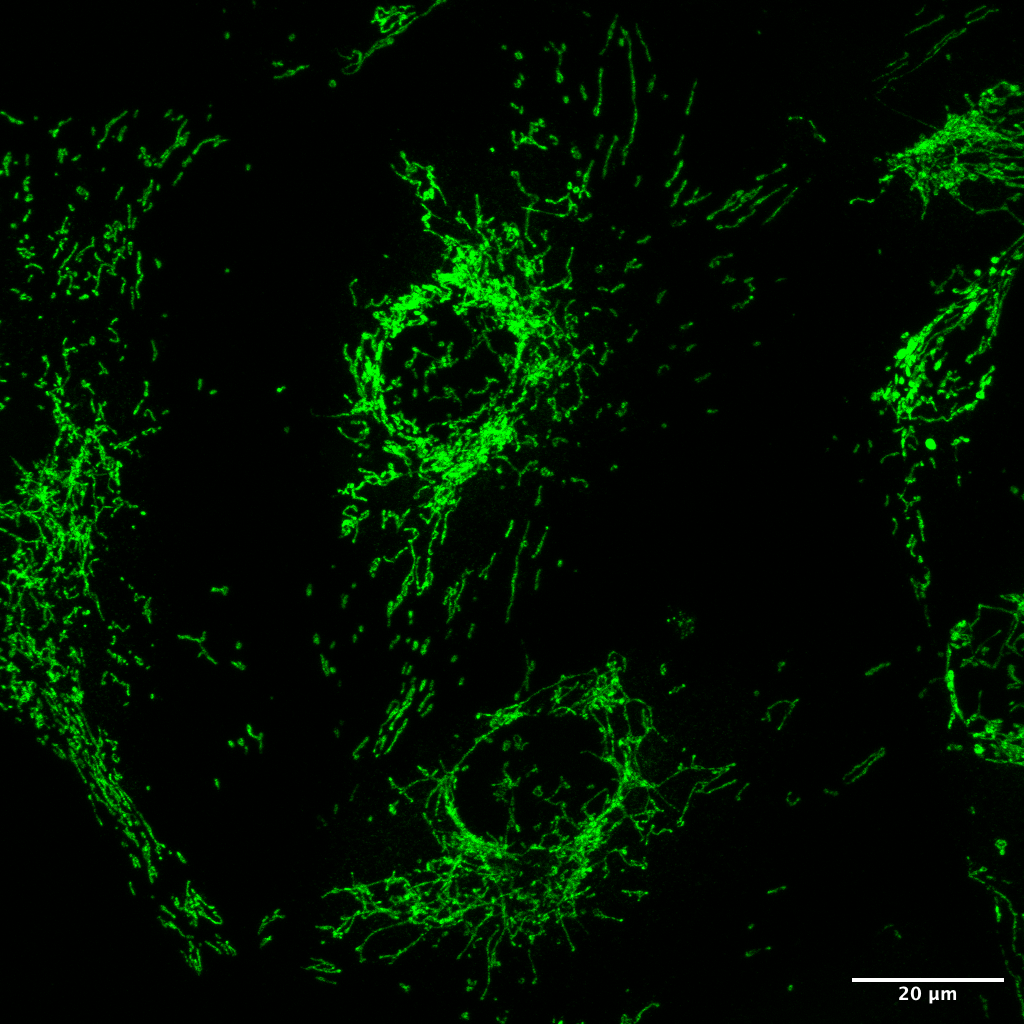

Supplement: Supplementary file 9 — Source data for all images: a zipped file divided into three folders for original source data images for Ki67 images, mouse images, and immunofluorescence images. Subfolders are labeled with the corresponding Figure number in which the image appears. [file 41586_2024_7812_MOESM9_ESM.zip › Images/Immunofluorescence/Extended_Data_Fig_9k_786-O_IF/Scale bar MAX_786-O CytoLbnox3.czi - C=1-1.tif]

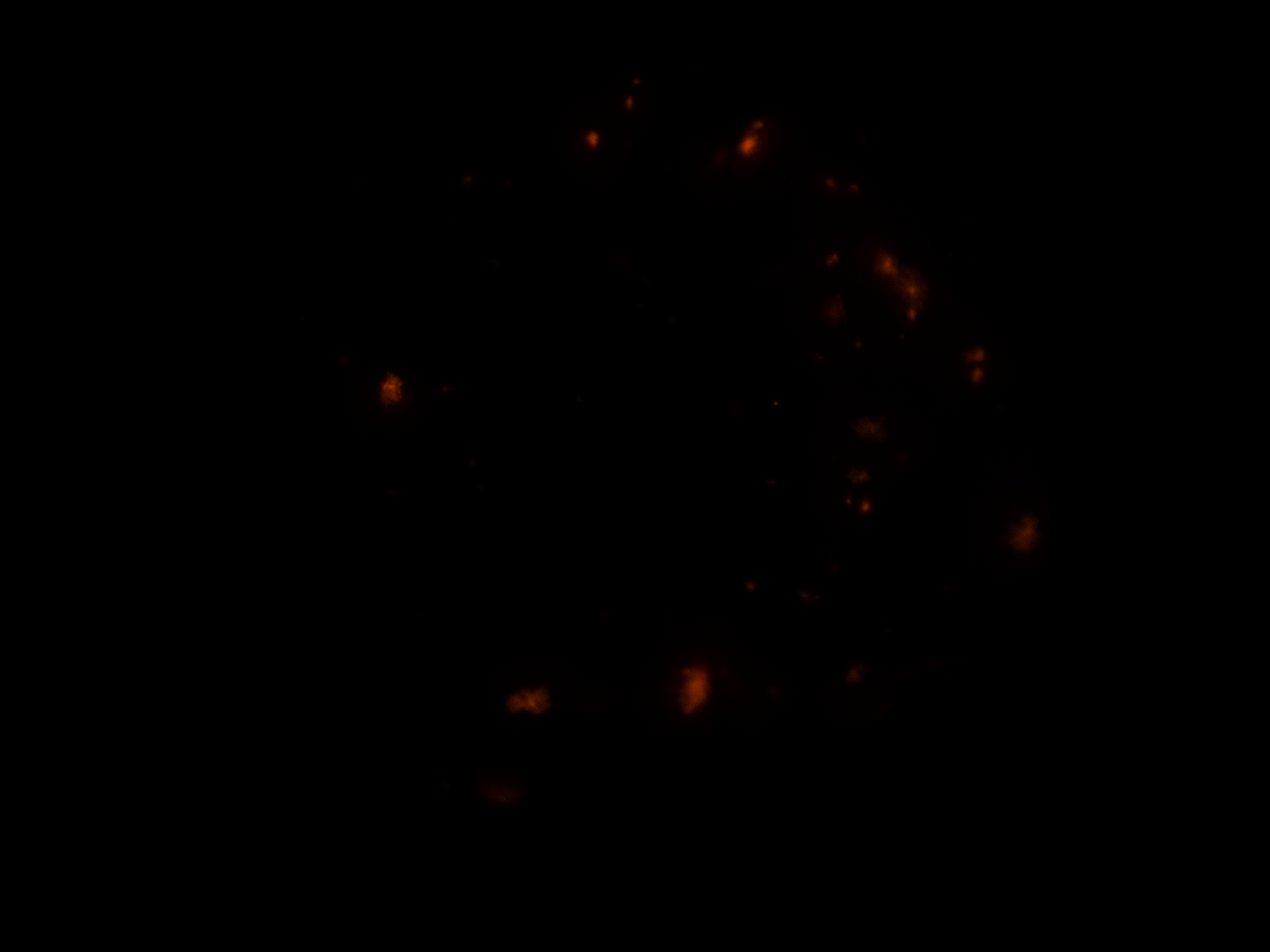

Supplement: Supplementary file 9 — Source data for all images: a zipped file divided into three folders for original source data images for Ki67 images, mouse images, and immunofluorescence images. Subfolders are labeled with the corresponding Figure number in which the image appears. [file 41586_2024_7812_MOESM9_ESM.zip › Images/Mouse Images/Fig_4j_mouse_images/Methigh_EV_2.tif]

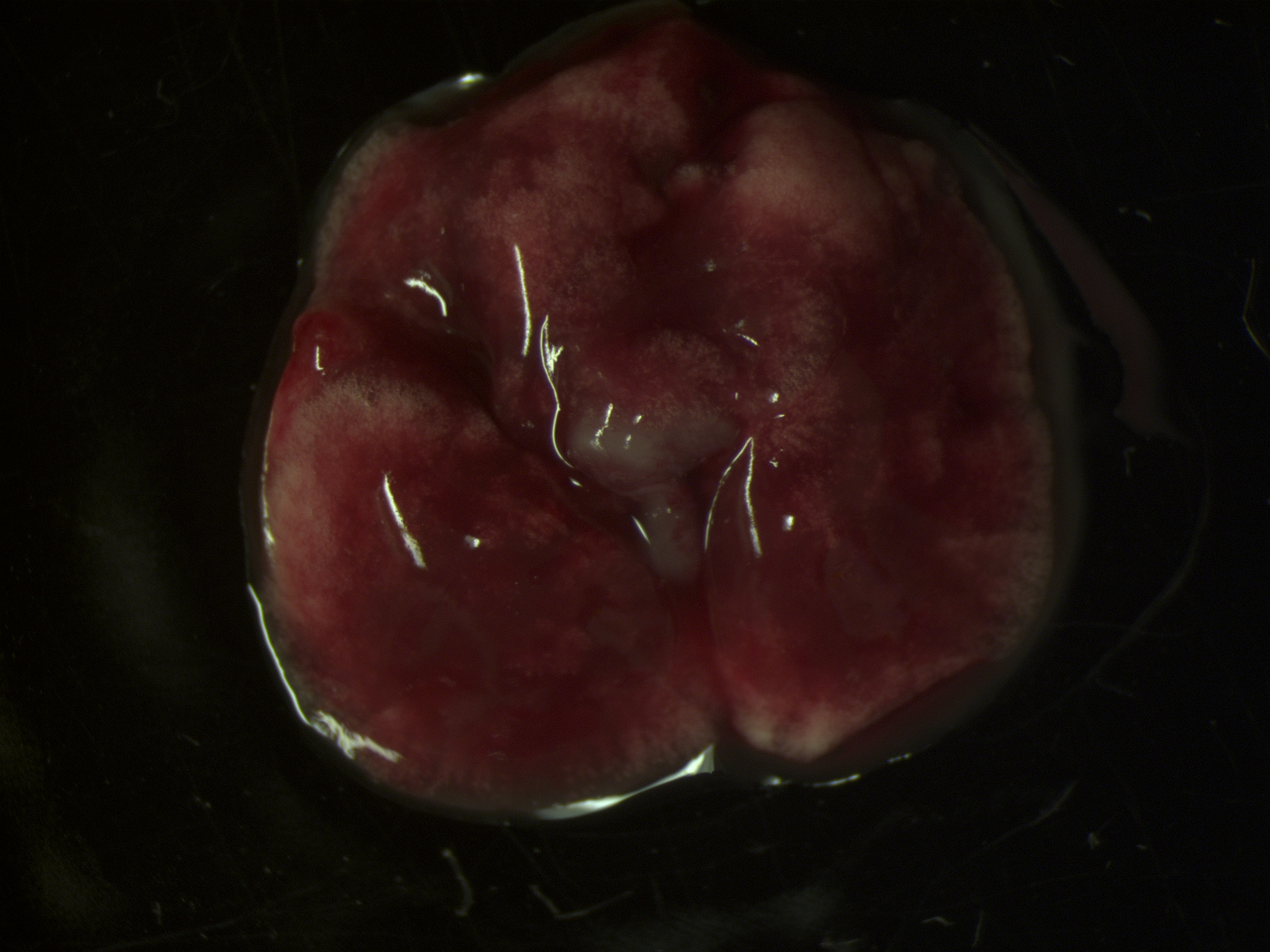

Supplement: Supplementary file 9 — Source data for all images: a zipped file divided into three folders for original source data images for Ki67 images, mouse images, and immunofluorescence images. Subfolders are labeled with the corresponding Figure number in which the image appears. [file 41586_2024_7812_MOESM9_ESM.zip › Images/Mouse Images/Fig_4j_mouse_images/Methigh_EV_1.tif]

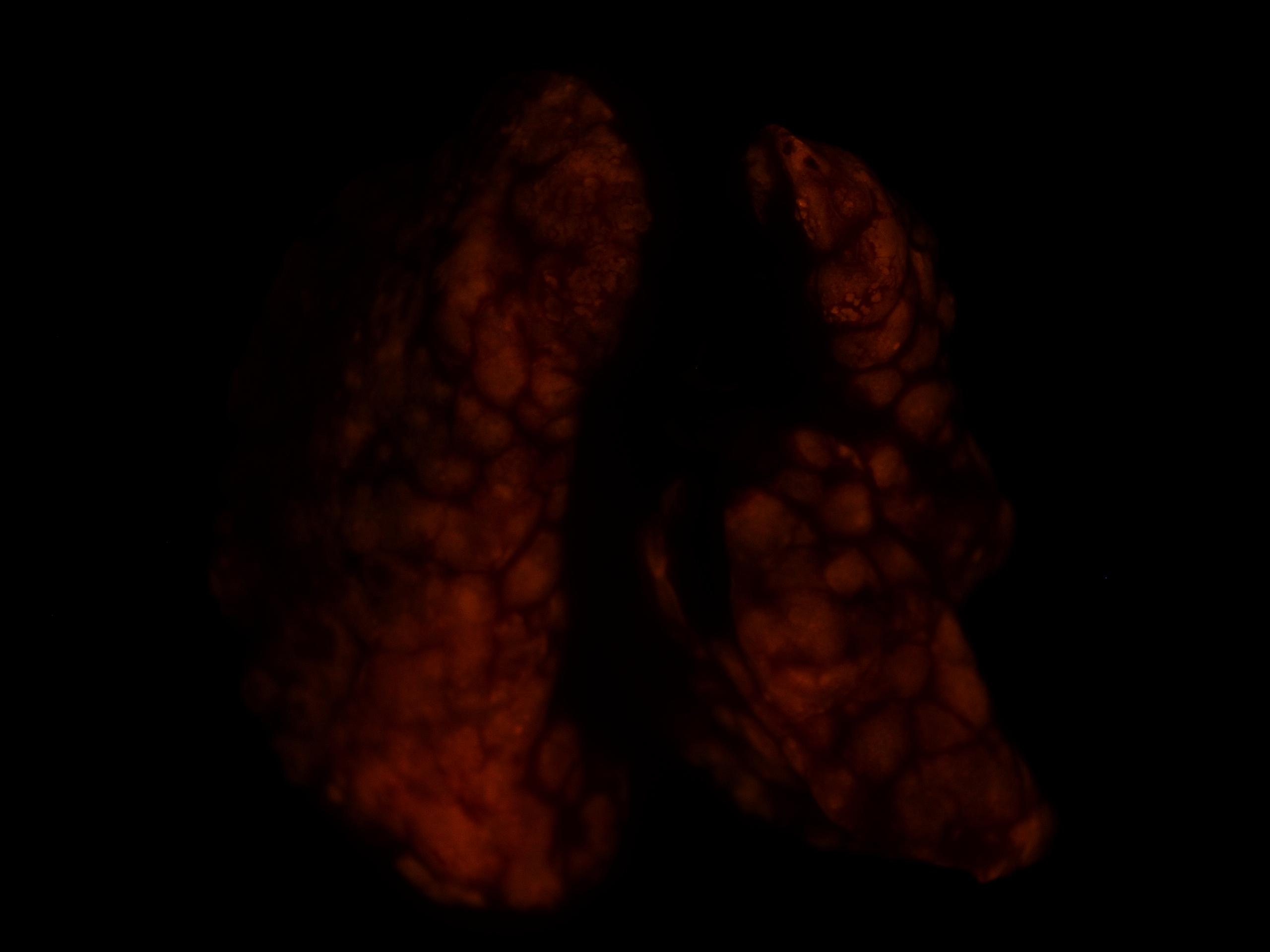

Supplement: Supplementary file 9 — Source data for all images: a zipped file divided into three folders for original source data images for Ki67 images, mouse images, and immunofluorescence images. Subfolders are labeled with the corresponding Figure number in which the image appears. [file 41586_2024_7812_MOESM9_ESM.zip › Images/Mouse Images/Fig_4j_mouse_images/Methigh_NDI1_2.tif]

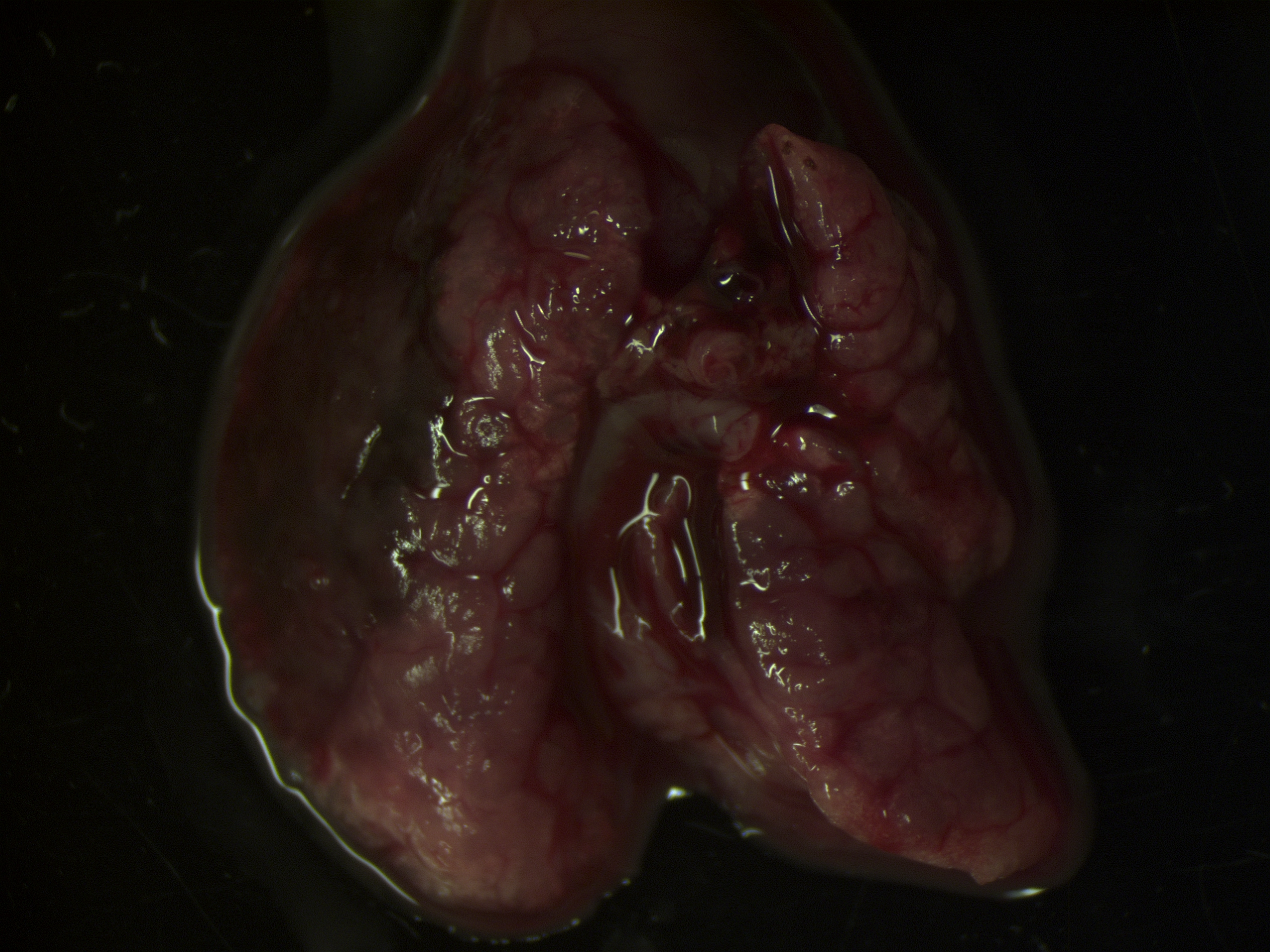

Supplement: Supplementary file 9 — Source data for all images: a zipped file divided into three folders for original source data images for Ki67 images, mouse images, and immunofluorescence images. Subfolders are labeled with the corresponding Figure number in which the image appears. [file 41586_2024_7812_MOESM9_ESM.zip › Images/Mouse Images/Fig_4j_mouse_images/Methigh_NDI1_1.tif]

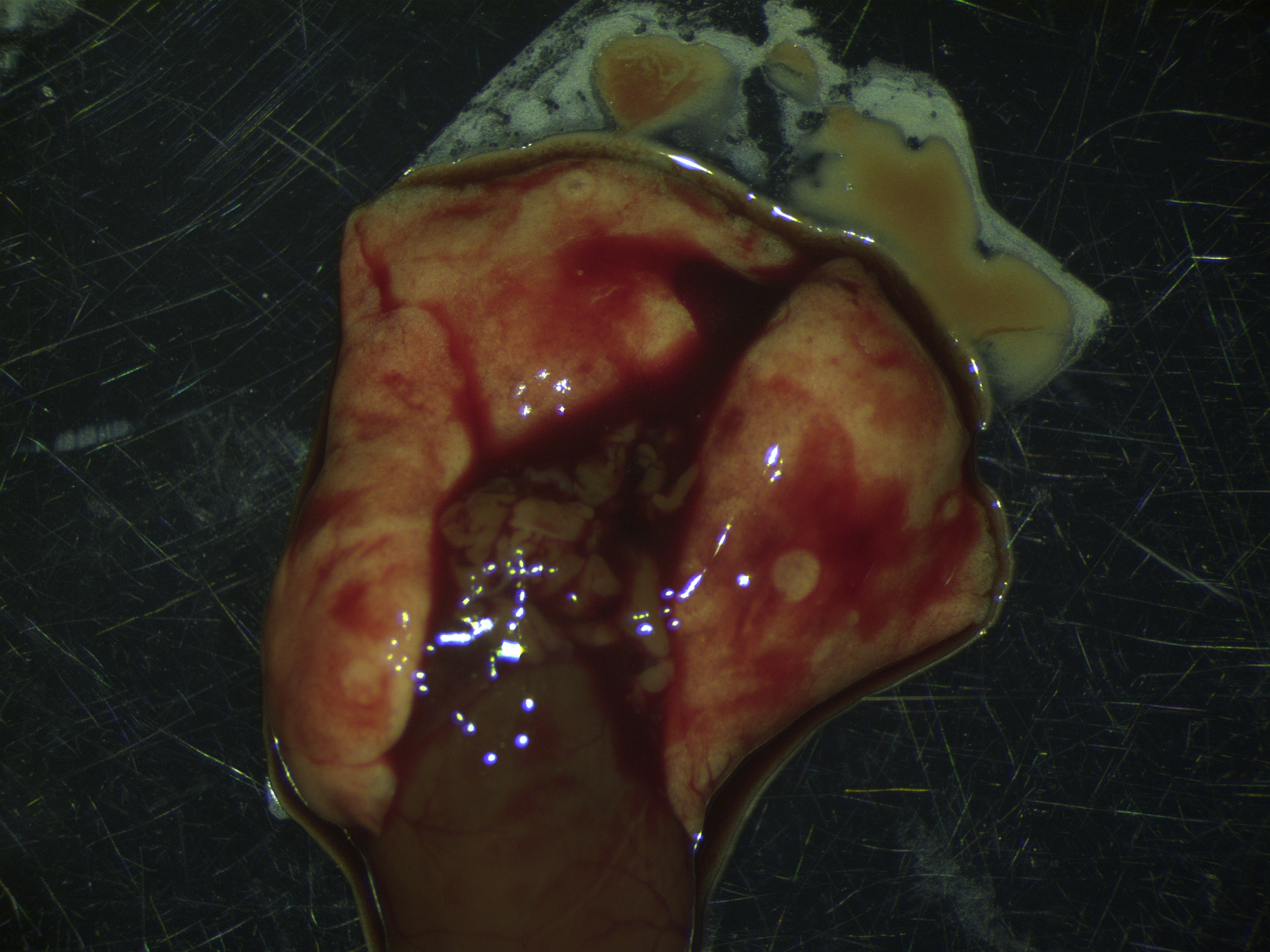

Supplement: Supplementary file 9 — Source data for all images: a zipped file divided into three folders for original source data images for Ki67 images, mouse images, and immunofluorescence images. Subfolders are labeled with the corresponding Figure number in which the image appears. [file 41586_2024_7812_MOESM9_ESM.zip › Images/Mouse Images/Extended_Data_FIg_8_mouse_images/a/Methig50_IACS_1.tif]

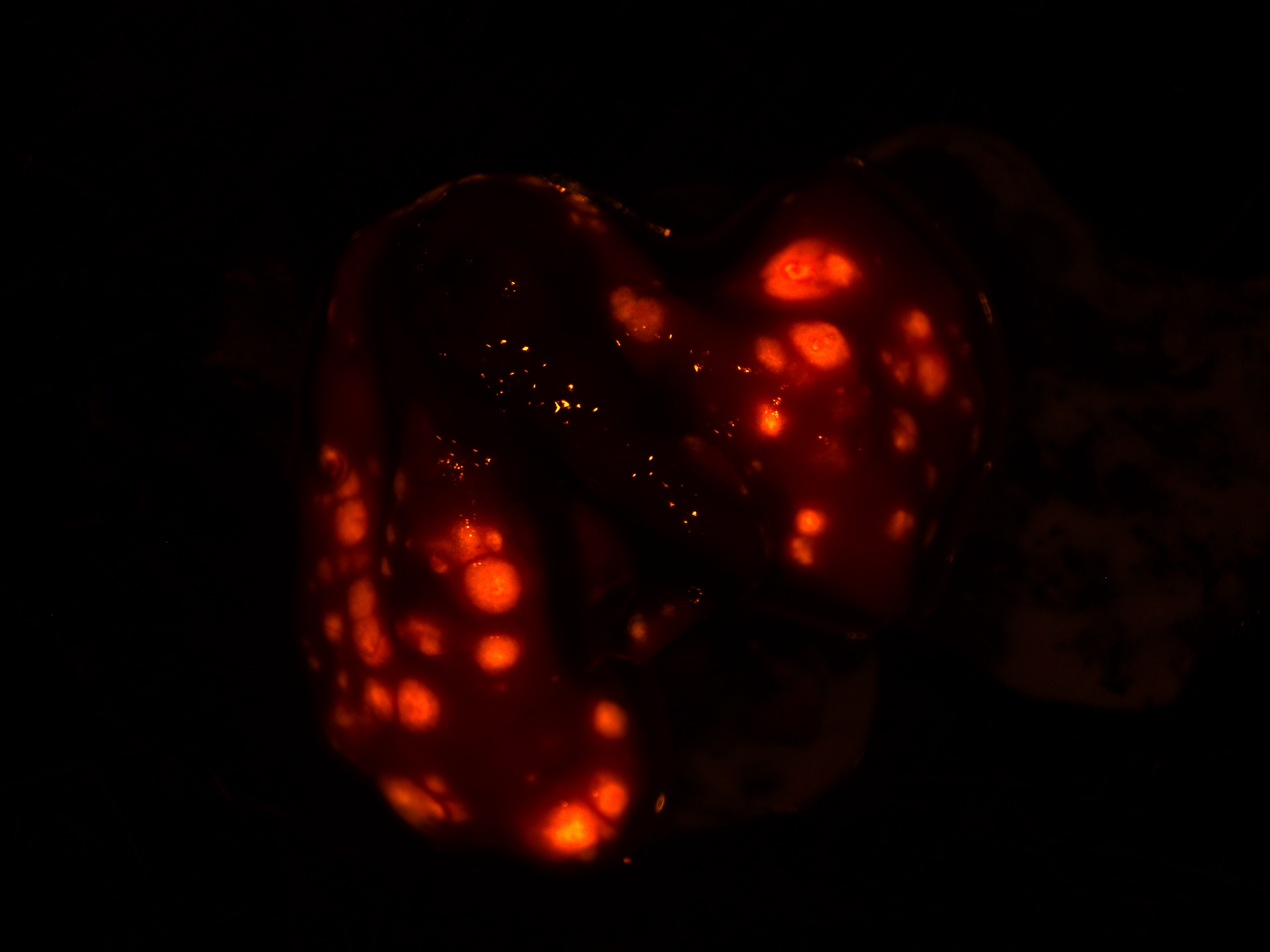

Supplement: Supplementary file 9 — Source data for all images: a zipped file divided into three folders for original source data images for Ki67 images, mouse images, and immunofluorescence images. Subfolders are labeled with the corresponding Figure number in which the image appears. [file 41586_2024_7812_MOESM9_ESM.zip › Images/Mouse Images/Extended_Data_FIg_8_mouse_images/a/Methigh50_Vehicle_2.tif]

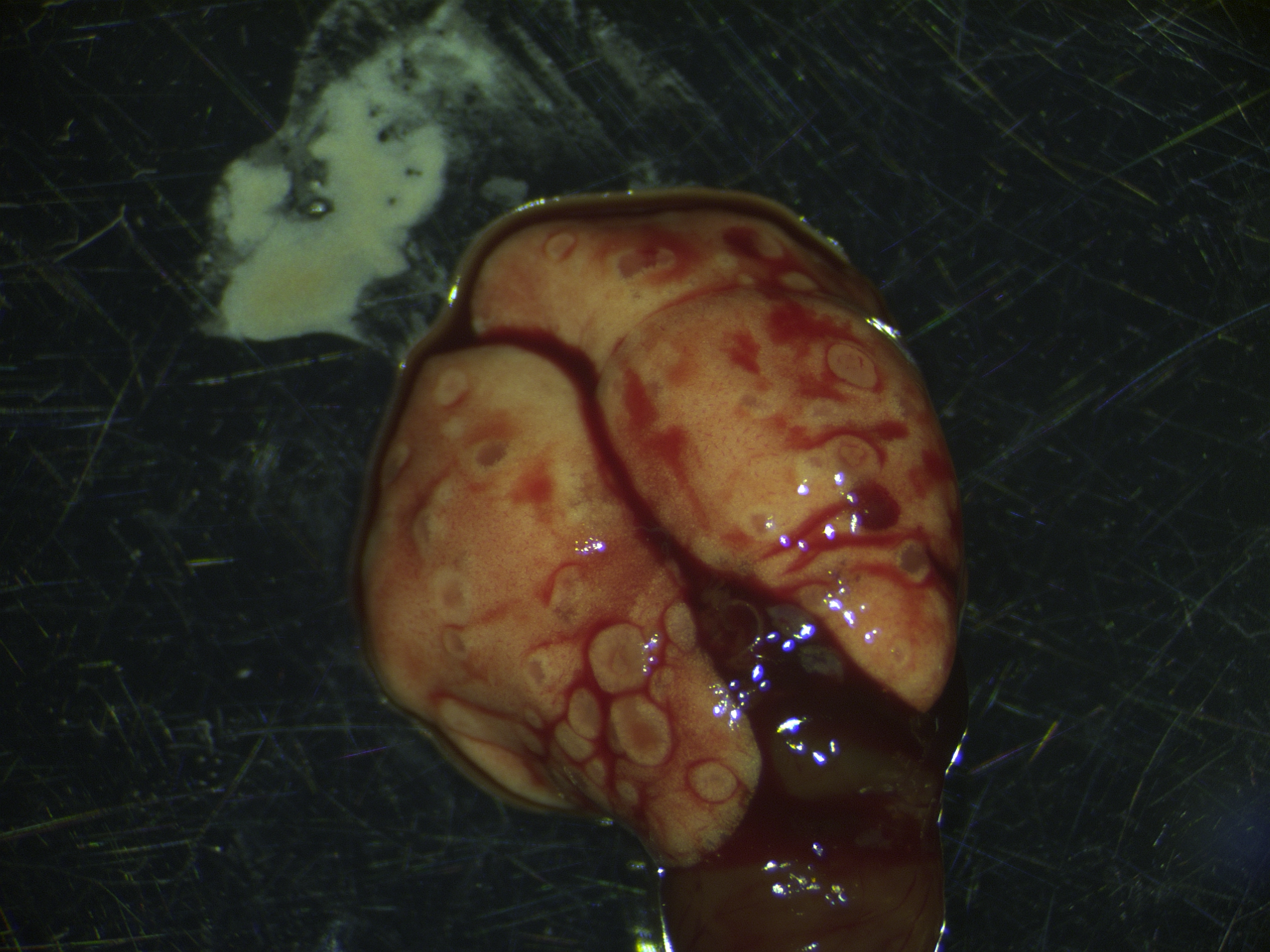

Supplement: Supplementary file 9 — Source data for all images: a zipped file divided into three folders for original source data images for Ki67 images, mouse images, and immunofluorescence images. Subfolders are labeled with the corresponding Figure number in which the image appears. [file 41586_2024_7812_MOESM9_ESM.zip › Images/Mouse Images/Extended_Data_FIg_8_mouse_images/a/Methigh_Vehicle_1.tif]

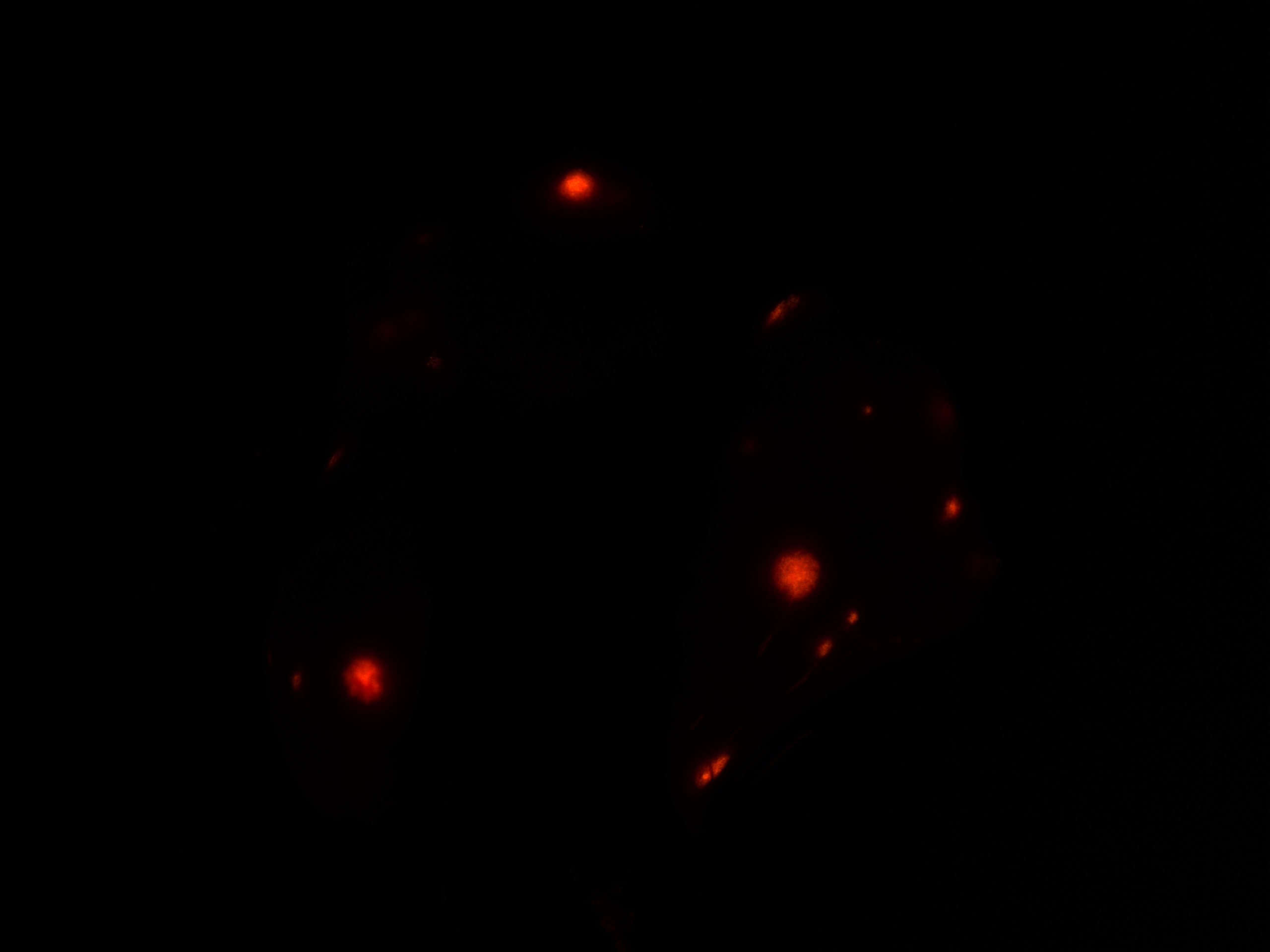

Supplement: Supplementary file 9 — Source data for all images: a zipped file divided into three folders for original source data images for Ki67 images, mouse images, and immunofluorescence images. Subfolders are labeled with the corresponding Figure number in which the image appears. [file 41586_2024_7812_MOESM9_ESM.zip › Images/Mouse Images/Extended_Data_FIg_8_mouse_images/a/Methigh50_IACS_2.tif]

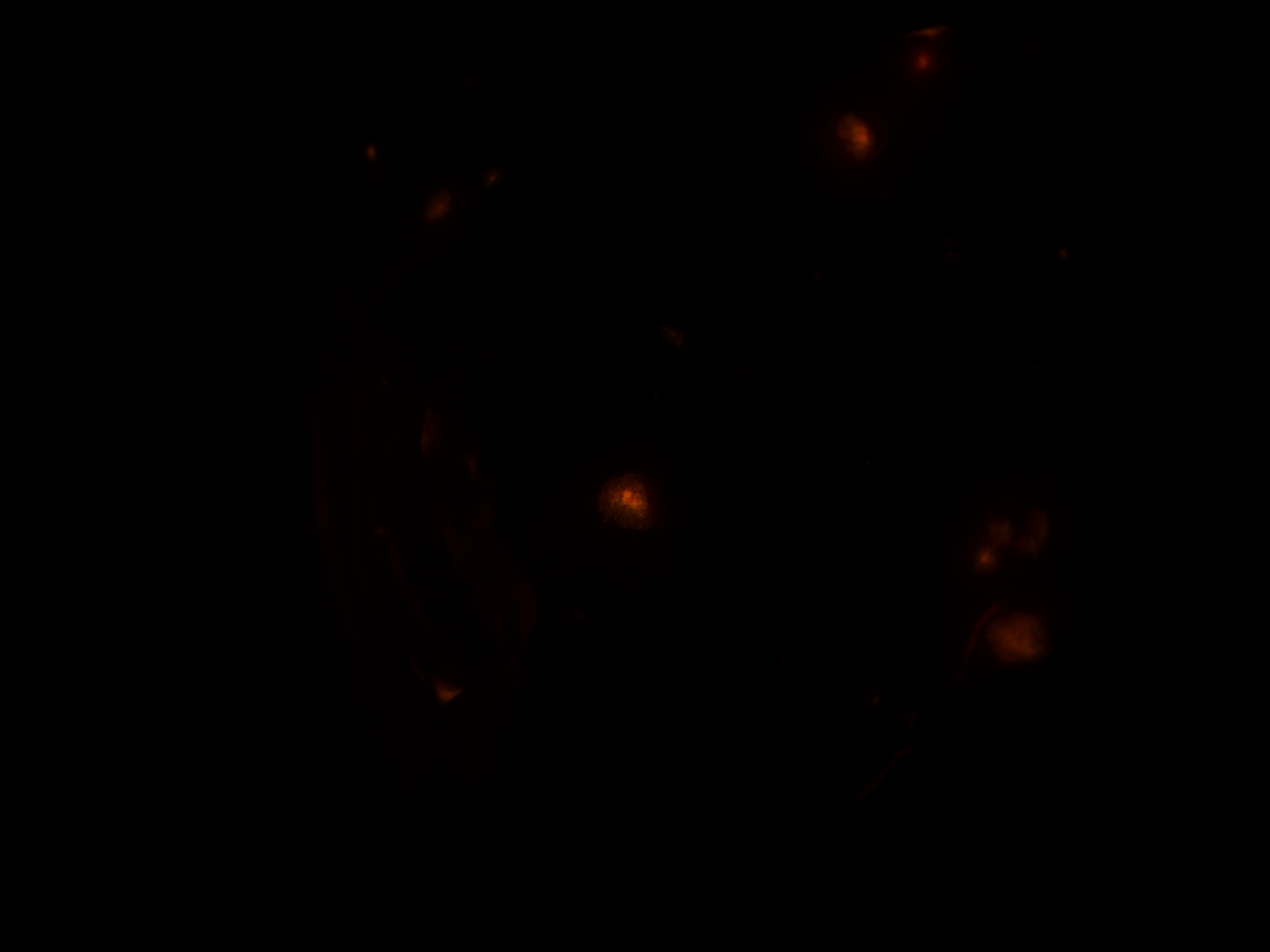

Supplement: Supplementary file 9 — Source data for all images: a zipped file divided into three folders for original source data images for Ki67 images, mouse images, and immunofluorescence images. Subfolders are labeled with the corresponding Figure number in which the image appears. [file 41586_2024_7812_MOESM9_ESM.zip › Images/Mouse Images/Extended_Data_FIg_8_mouse_images/f/Methigh_IACS_2.tif]

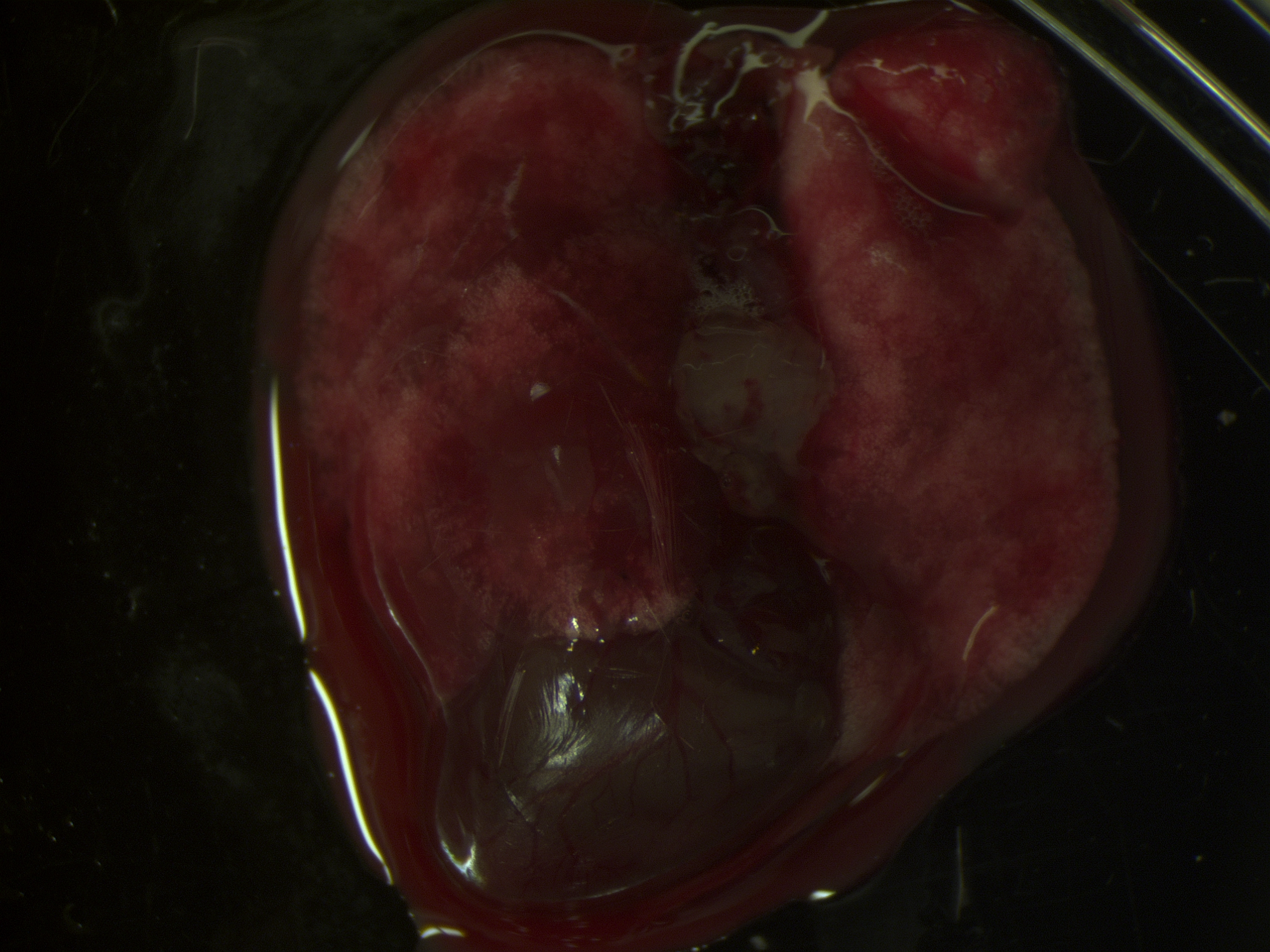

Supplement: Supplementary file 9 — Source data for all images: a zipped file divided into three folders for original source data images for Ki67 images, mouse images, and immunofluorescence images. Subfolders are labeled with the corresponding Figure number in which the image appears. [file 41586_2024_7812_MOESM9_ESM.zip › Images/Mouse Images/Extended_Data_FIg_8_mouse_images/f/Methigh_IACS_1.tif]

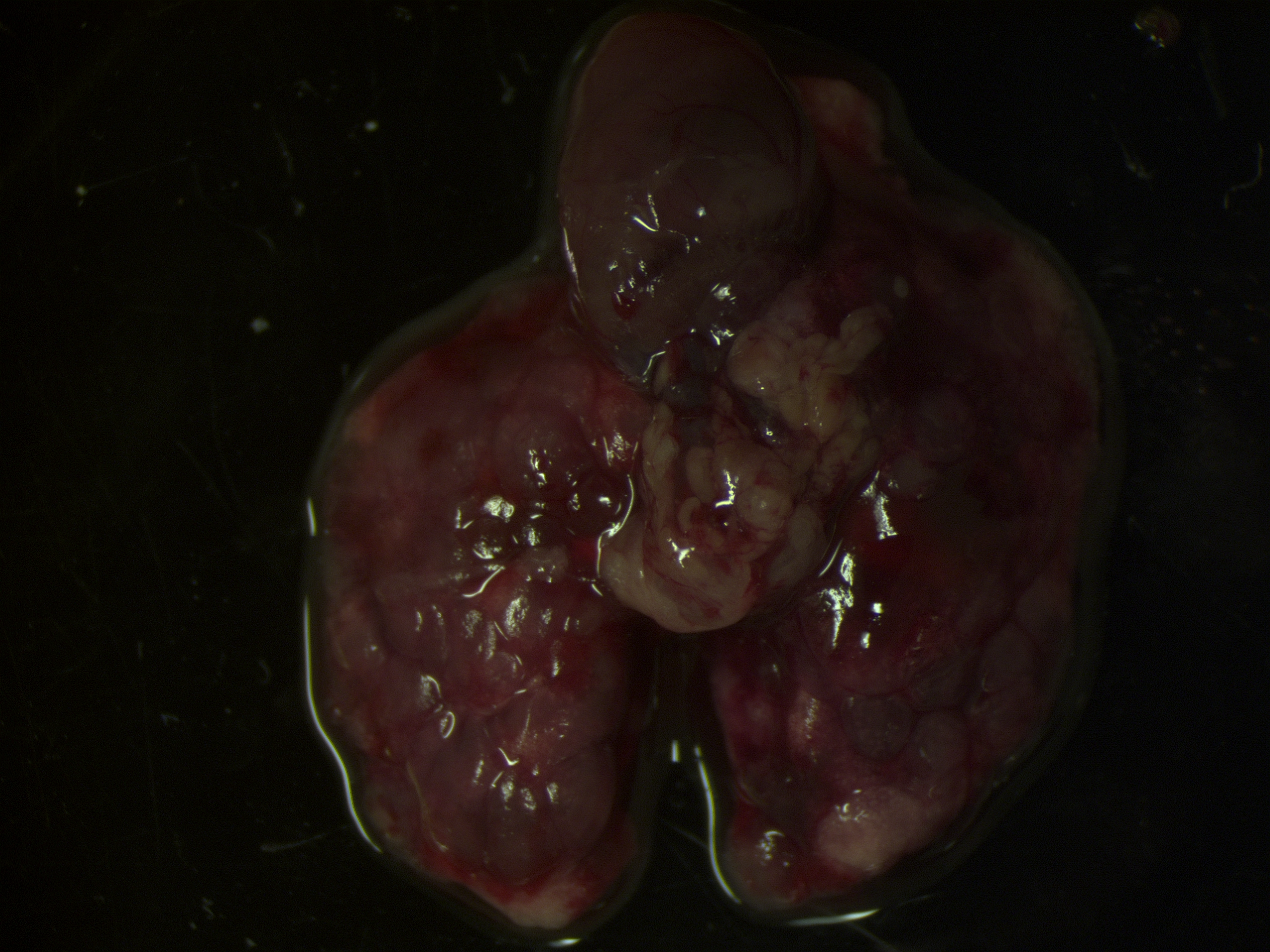

Supplement: Supplementary file 9 — Source data for all images: a zipped file divided into three folders for original source data images for Ki67 images, mouse images, and immunofluorescence images. Subfolders are labeled with the corresponding Figure number in which the image appears. [file 41586_2024_7812_MOESM9_ESM.zip › Images/Mouse Images/Extended_Data_FIg_8_mouse_images/f/Methigh_Vehicle_1.tif]

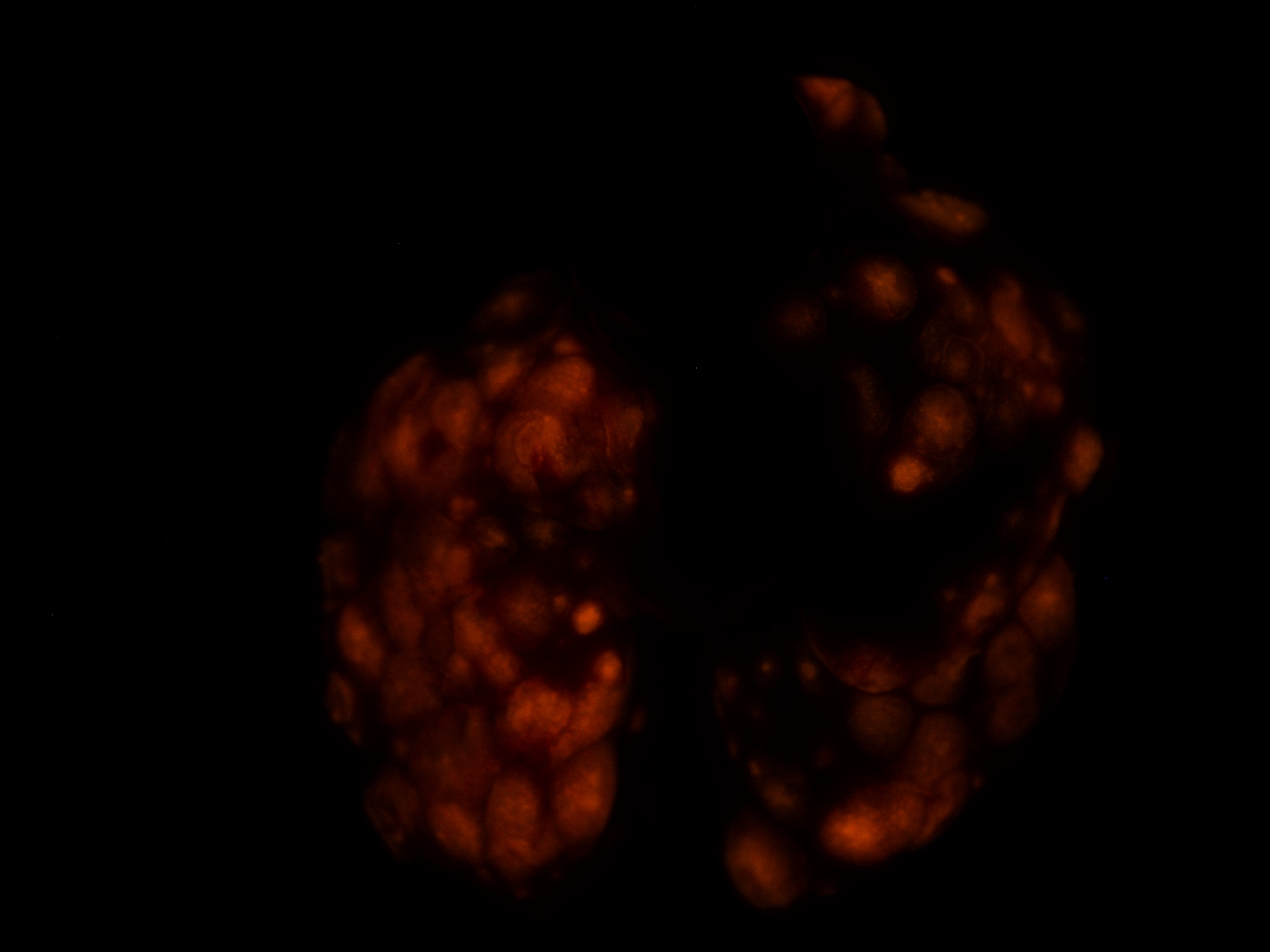

Supplement: Supplementary file 9 — Source data for all images: a zipped file divided into three folders for original source data images for Ki67 images, mouse images, and immunofluorescence images. Subfolders are labeled with the corresponding Figure number in which the image appears. [file 41586_2024_7812_MOESM9_ESM.zip › Images/Mouse Images/Extended_Data_FIg_8_mouse_images/f/Methigh_Vehicle_2.tif]

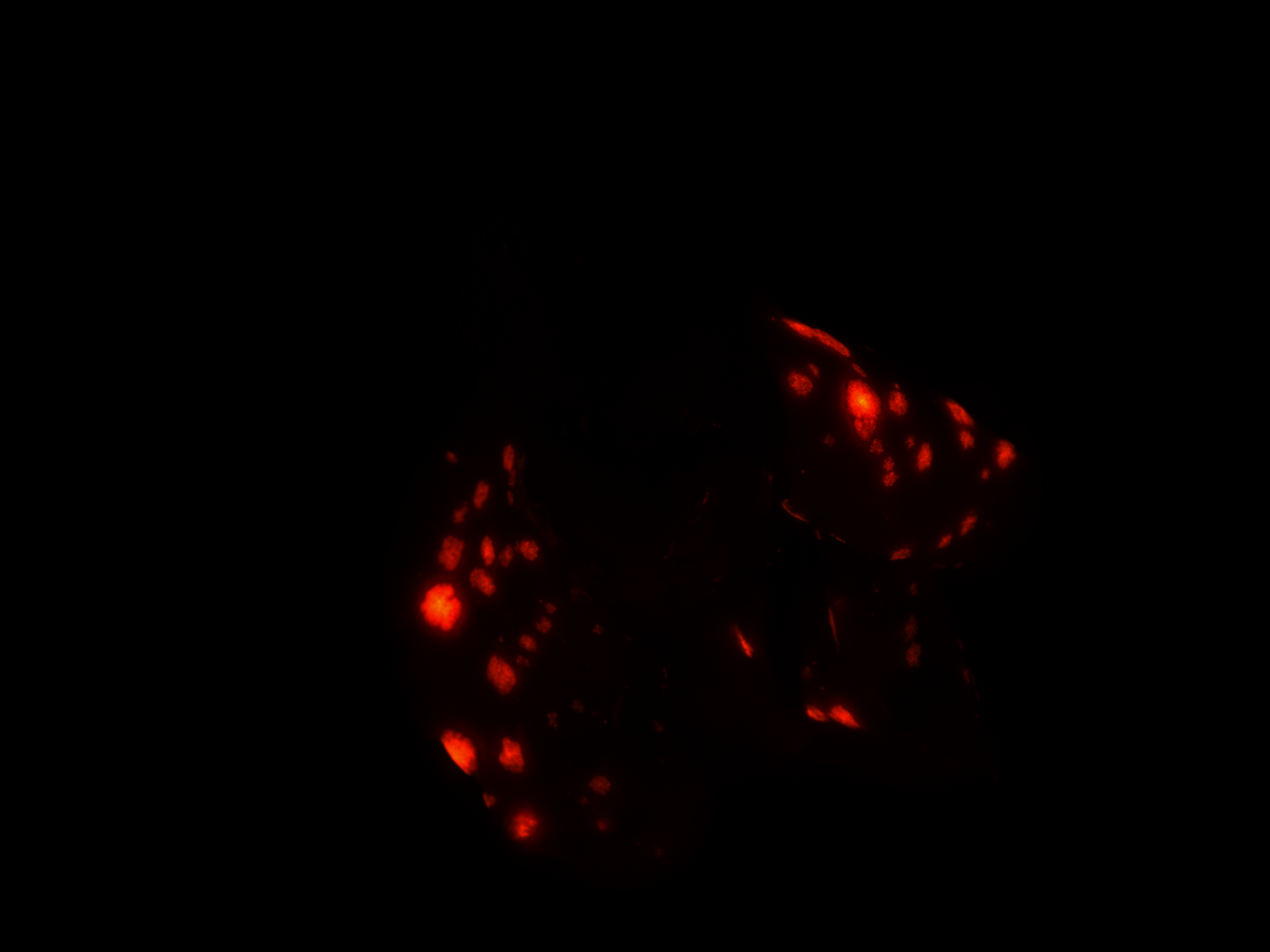

Supplement: Supplementary file 9 — Source data for all images: a zipped file divided into three folders for original source data images for Ki67 images, mouse images, and immunofluorescence images. Subfolders are labeled with the corresponding Figure number in which the image appears. [file 41586_2024_7812_MOESM9_ESM.zip › Images/Mouse Images/Extended_Data_FIg_8_mouse_images/o/metlow_mito_lbnox_2.tif]

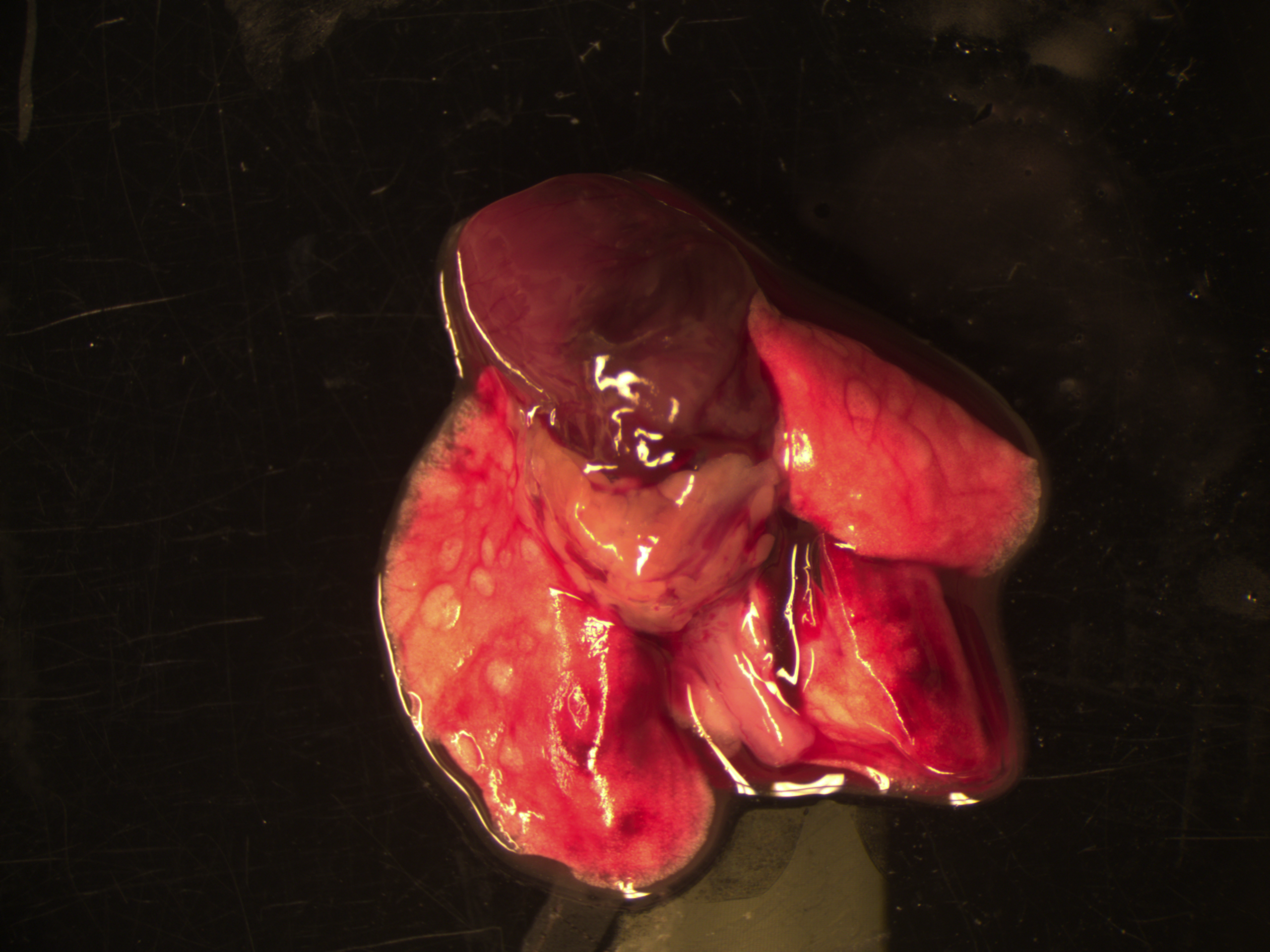

Supplement: Supplementary file 9 — Source data for all images: a zipped file divided into three folders for original source data images for Ki67 images, mouse images, and immunofluorescence images. Subfolders are labeled with the corresponding Figure number in which the image appears. [file 41586_2024_7812_MOESM9_ESM.zip › Images/Mouse Images/Extended_Data_FIg_8_mouse_images/o/metlow_mito_lbnox_1.tif]

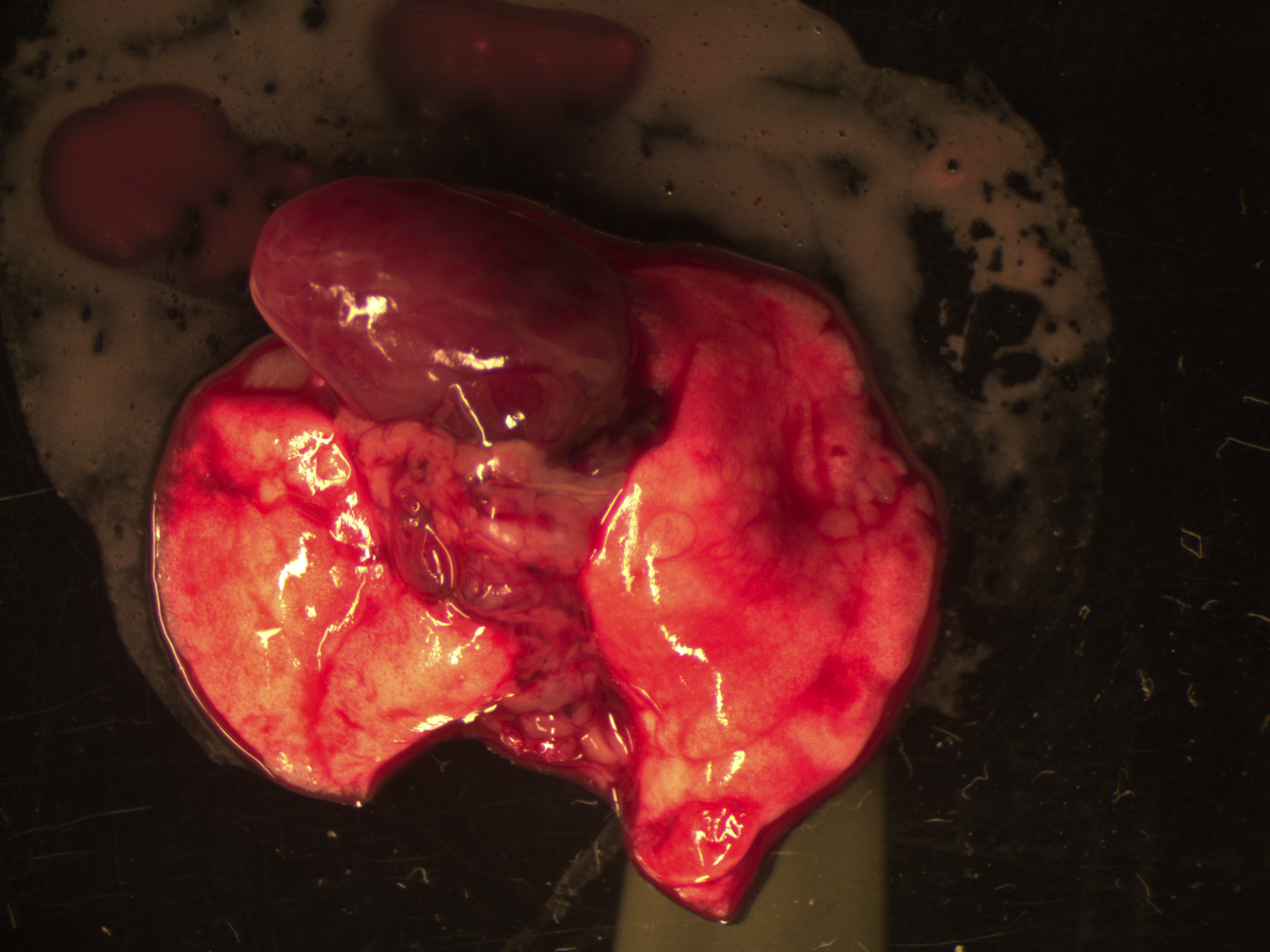

Supplement: Supplementary file 9 — Source data for all images: a zipped file divided into three folders for original source data images for Ki67 images, mouse images, and immunofluorescence images. Subfolders are labeled with the corresponding Figure number in which the image appears. [file 41586_2024_7812_MOESM9_ESM.zip › Images/Mouse Images/Extended_Data_FIg_8_mouse_images/o/Metlow_cyto_lbnox_1.tif]

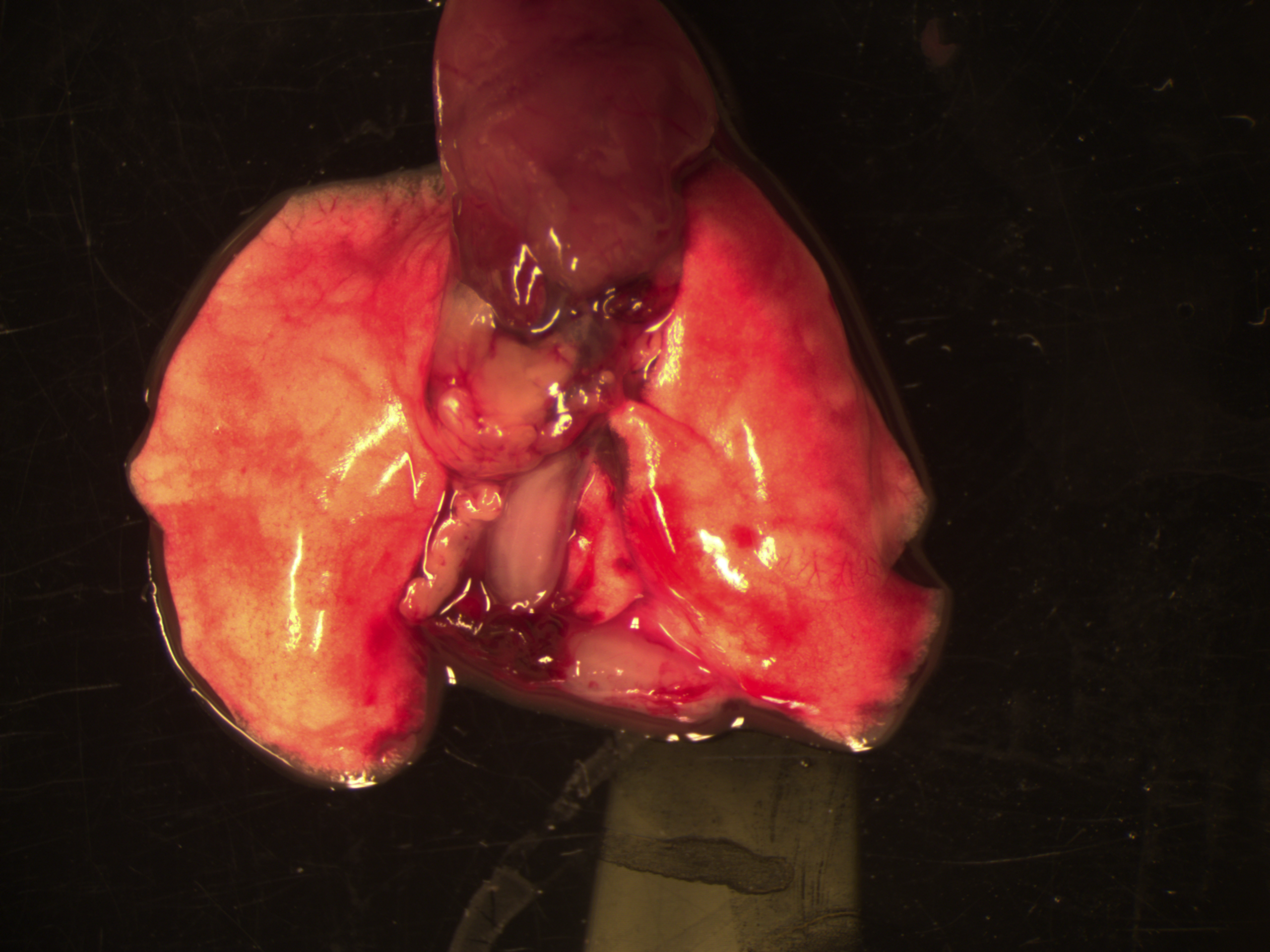

Supplement: Supplementary file 9 — Source data for all images: a zipped file divided into three folders for original source data images for Ki67 images, mouse images, and immunofluorescence images. Subfolders are labeled with the corresponding Figure number in which the image appears. [file 41586_2024_7812_MOESM9_ESM.zip › Images/Mouse Images/Extended_Data_FIg_8_mouse_images/o/Metlow_EV_1.tif]

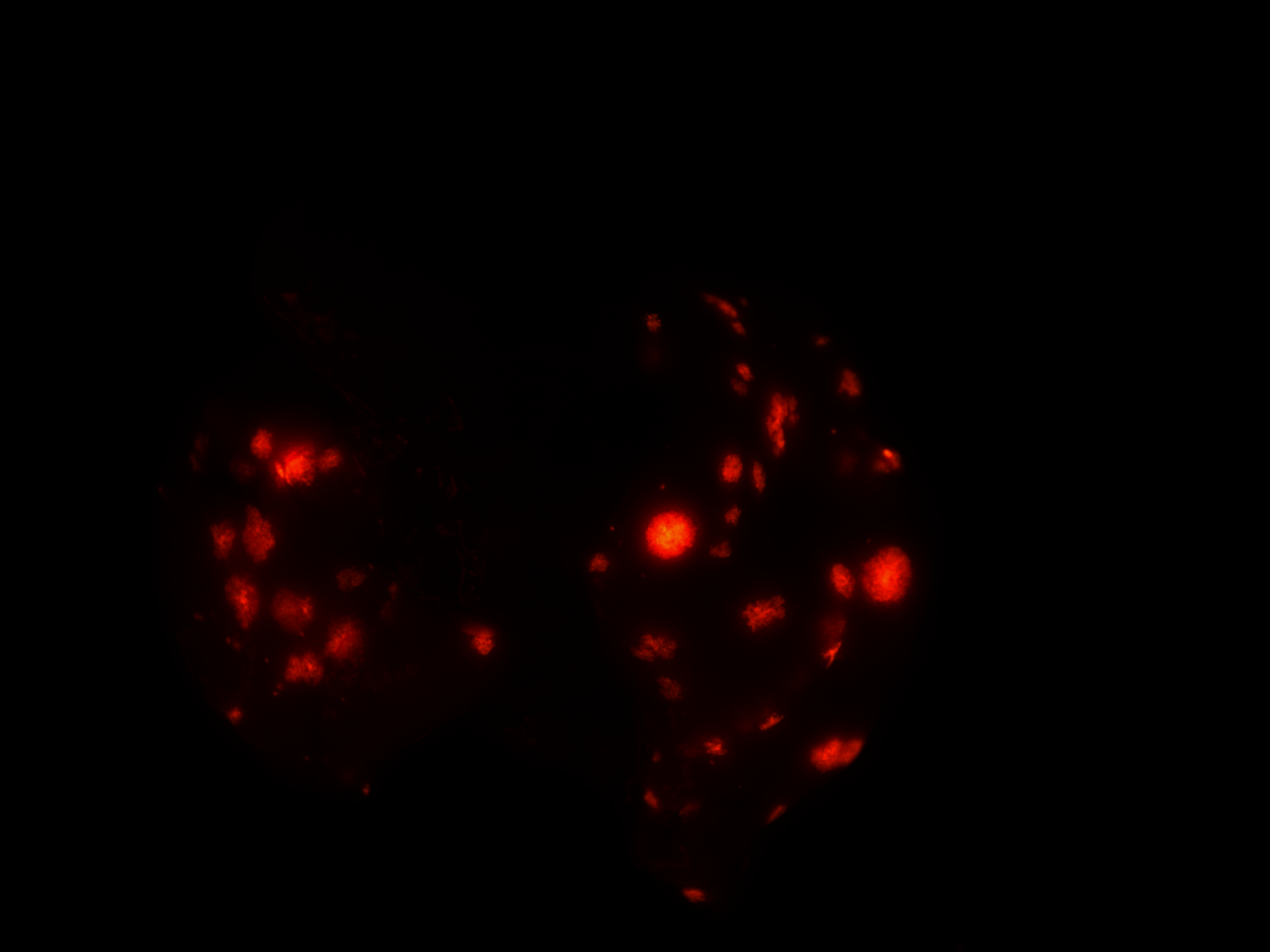

Supplement: Supplementary file 9 — Source data for all images: a zipped file divided into three folders for original source data images for Ki67 images, mouse images, and immunofluorescence images. Subfolders are labeled with the corresponding Figure number in which the image appears. [file 41586_2024_7812_MOESM9_ESM.zip › Images/Mouse Images/Extended_Data_FIg_8_mouse_images/o/Metlow_cyto_lbnox_2.tif]

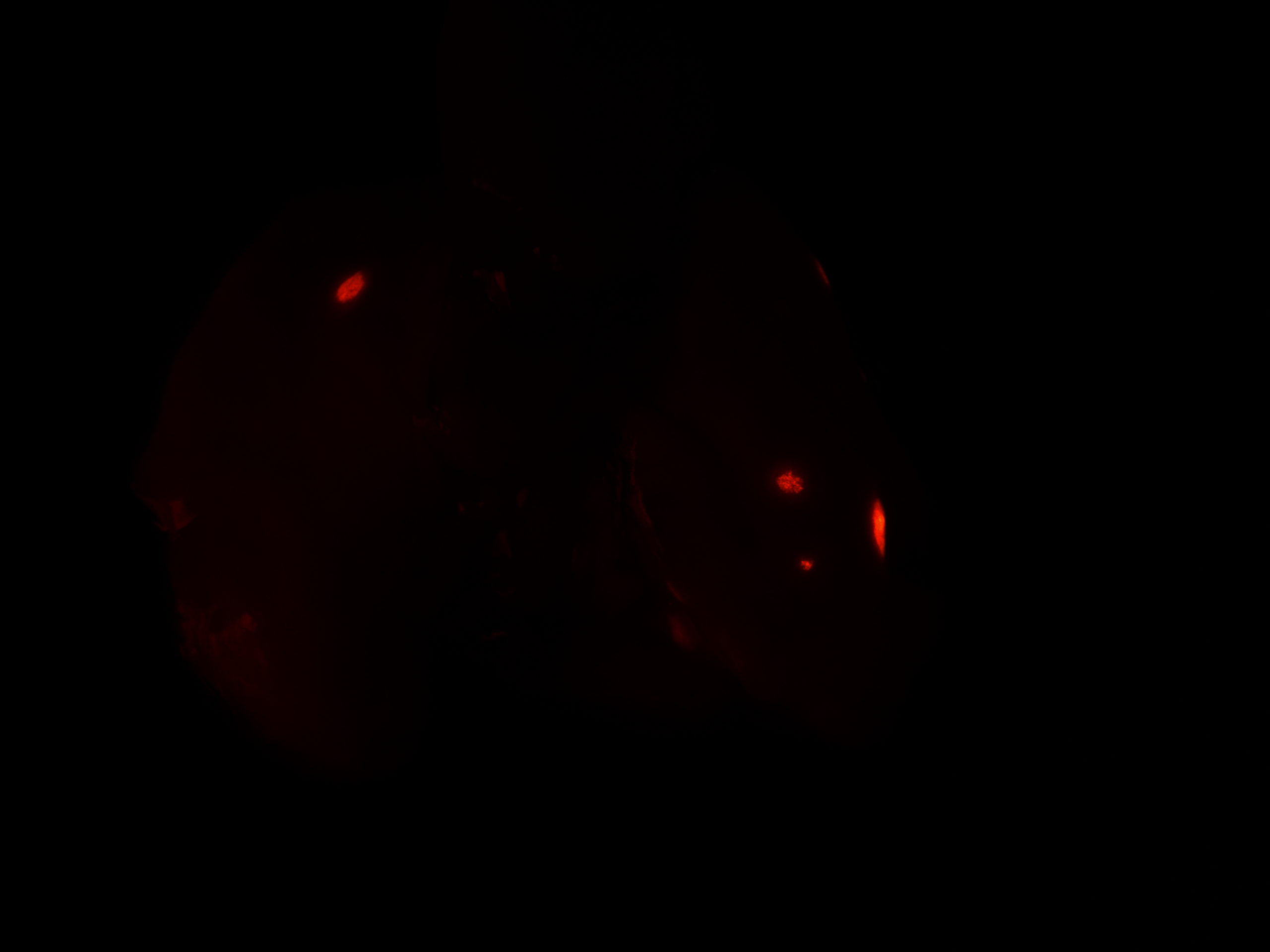

Supplement: Supplementary file 9 — Source data for all images: a zipped file divided into three folders for original source data images for Ki67 images, mouse images, and immunofluorescence images. Subfolders are labeled with the corresponding Figure number in which the image appears. [file 41586_2024_7812_MOESM9_ESM.zip › Images/Mouse Images/Extended_Data_FIg_8_mouse_images/o/Metlow_EV_2.tif]

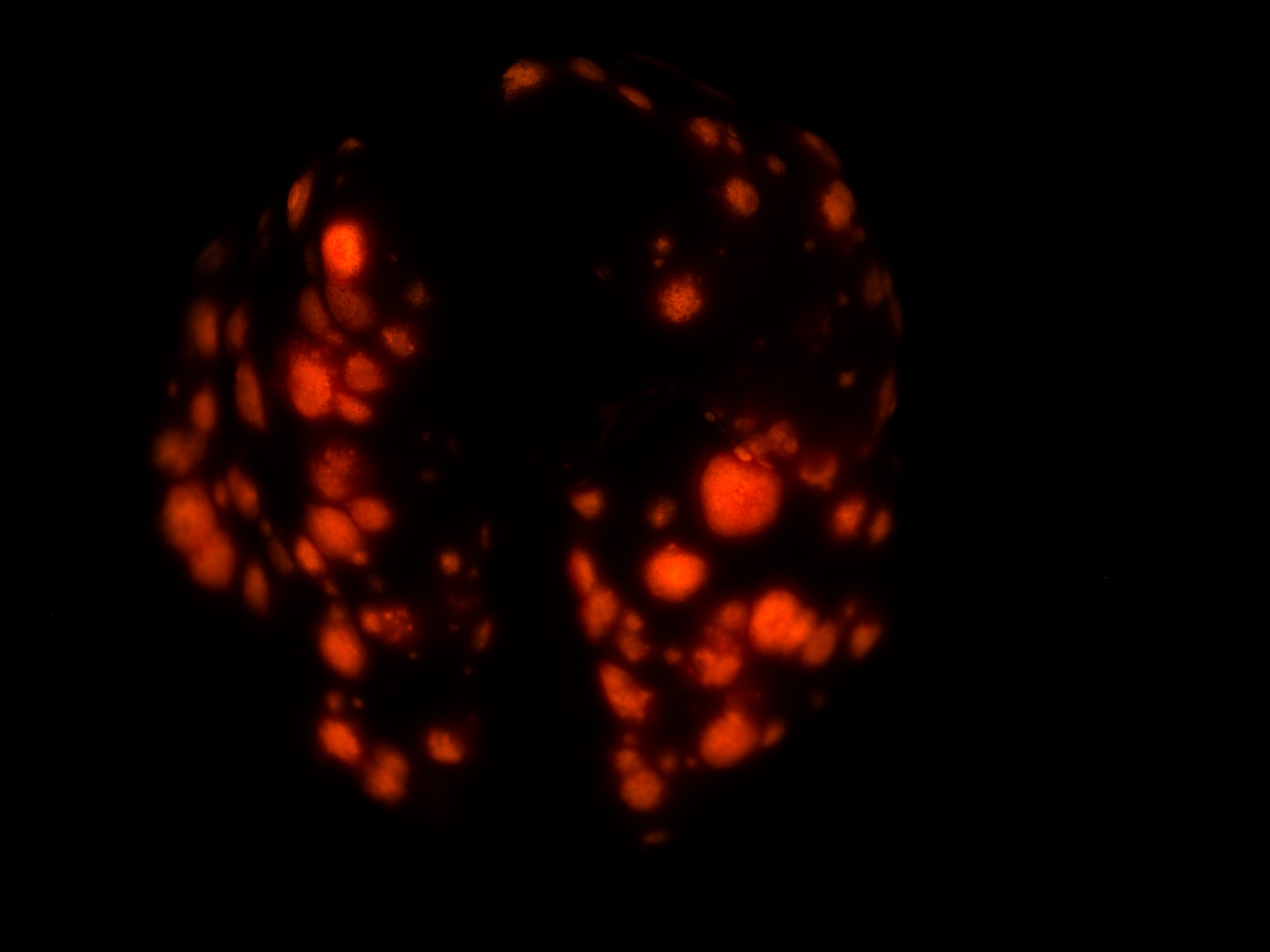

Supplement: Supplementary file 9 — Source data for all images: a zipped file divided into three folders for original source data images for Ki67 images, mouse images, and immunofluorescence images. Subfolders are labeled with the corresponding Figure number in which the image appears. [file 41586_2024_7812_MOESM9_ESM.zip › Images/Mouse Images/Extended_Data_FIg_8_mouse_images/b/Methigh26_Vehicle_2.tif]

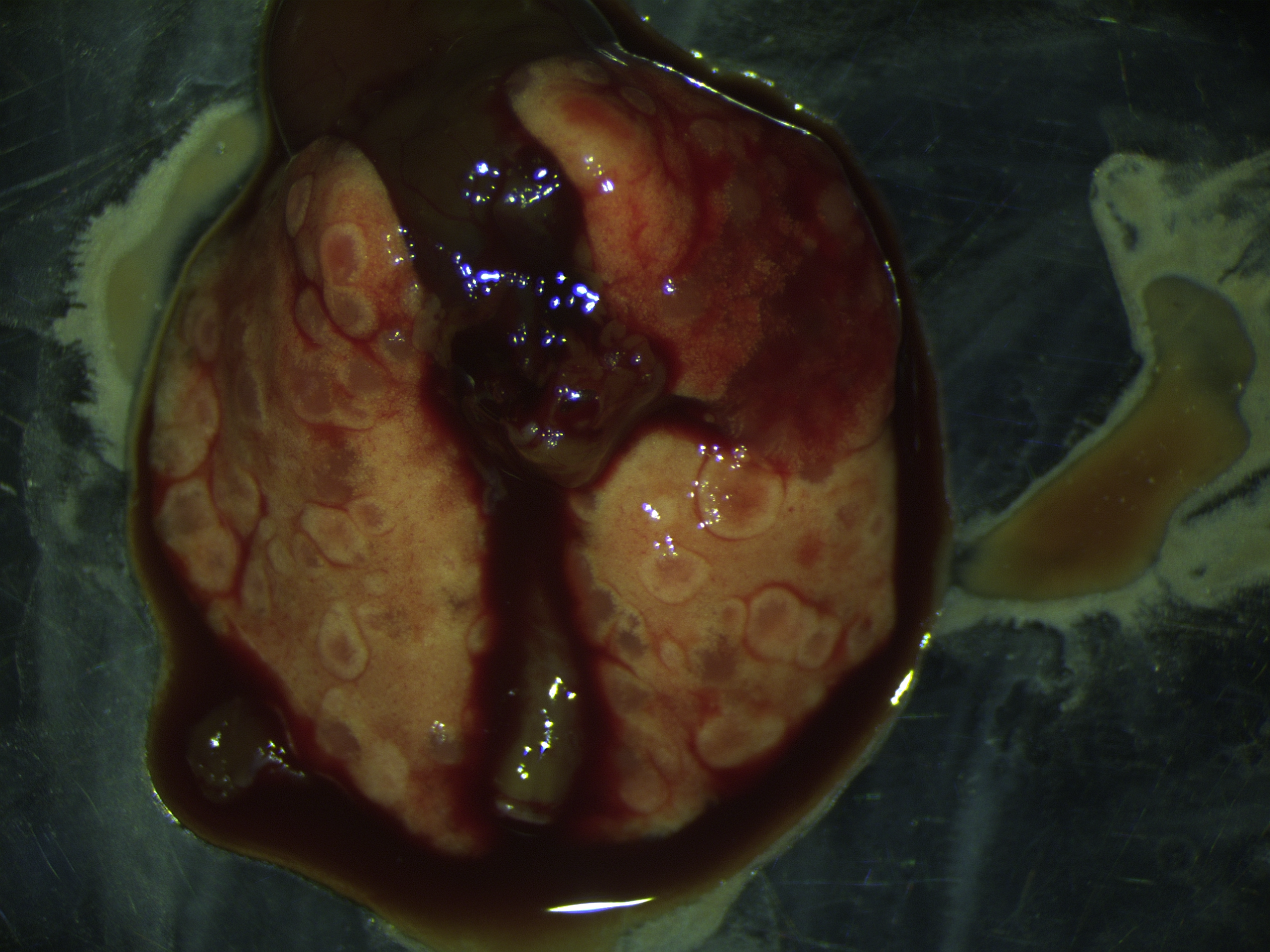

Supplement: Supplementary file 9 — Source data for all images: a zipped file divided into three folders for original source data images for Ki67 images, mouse images, and immunofluorescence images. Subfolders are labeled with the corresponding Figure number in which the image appears. [file 41586_2024_7812_MOESM9_ESM.zip › Images/Mouse Images/Extended_Data_FIg_8_mouse_images/b/Methigh26_Vehicle_1.tif]

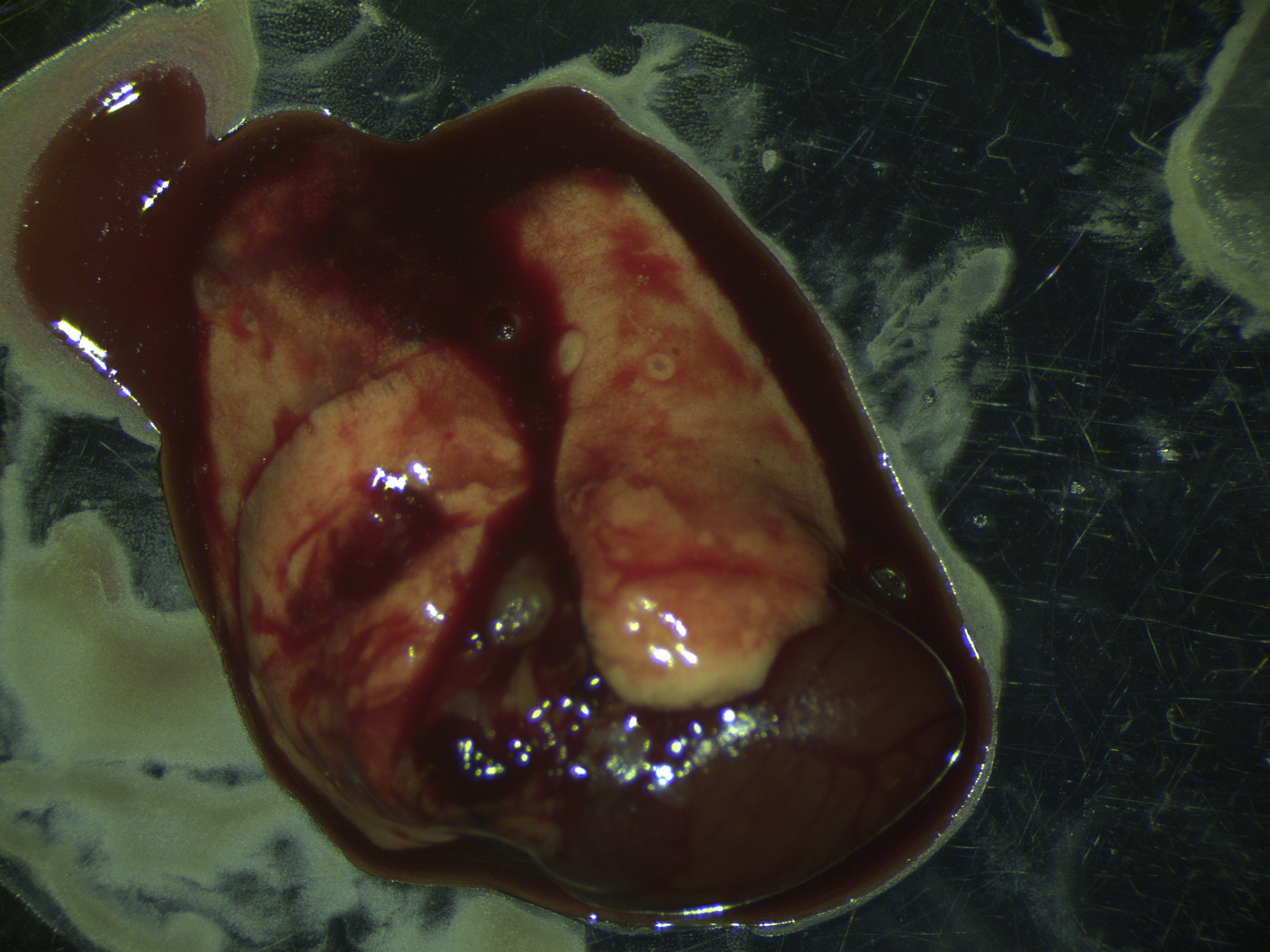

Supplement: Supplementary file 9 — Source data for all images: a zipped file divided into three folders for original source data images for Ki67 images, mouse images, and immunofluorescence images. Subfolders are labeled with the corresponding Figure number in which the image appears. [file 41586_2024_7812_MOESM9_ESM.zip › Images/Mouse Images/Extended_Data_FIg_8_mouse_images/b/Methigh26_IACS_1.tif]

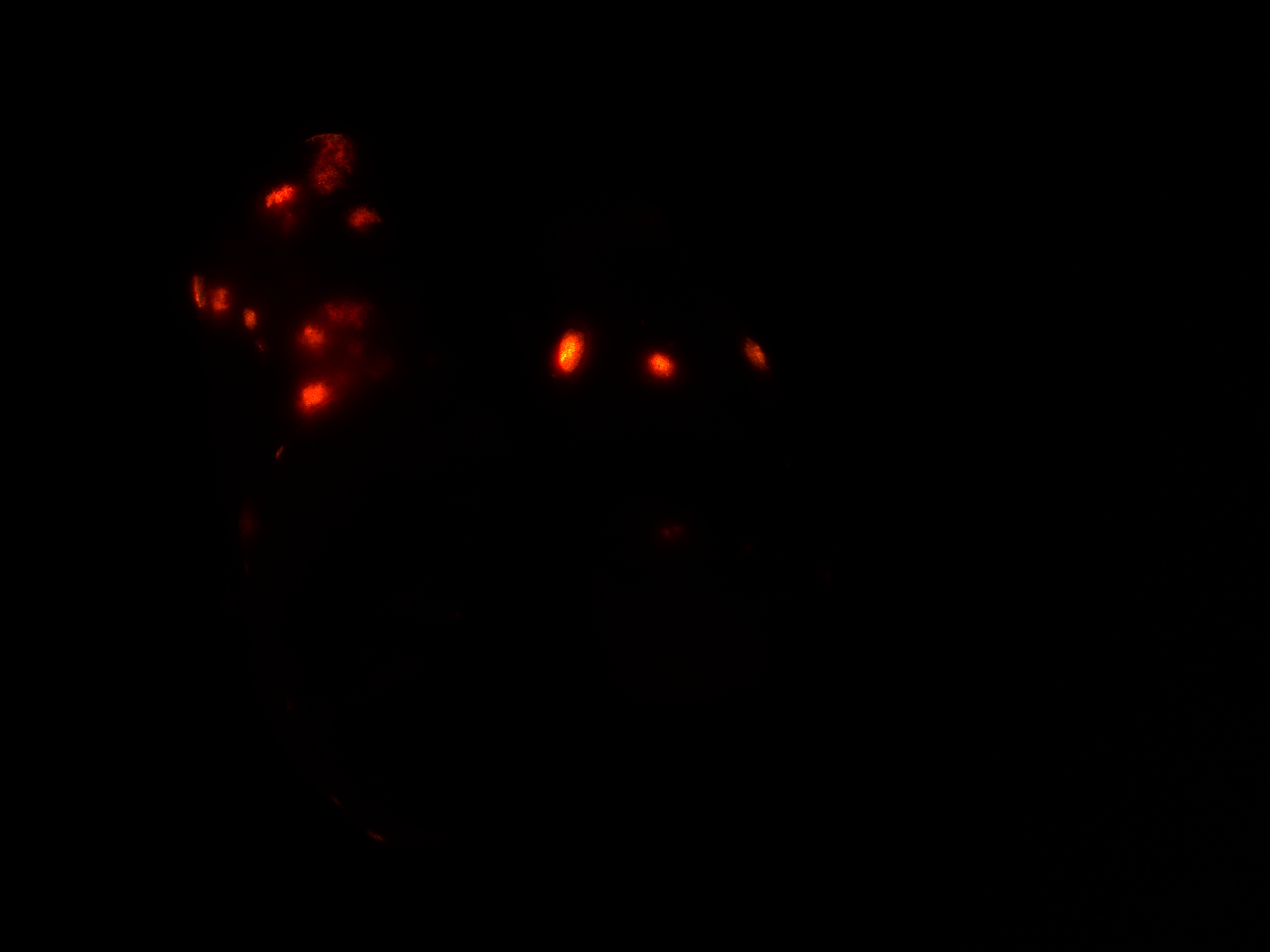

Supplement: Supplementary file 9 — Source data for all images: a zipped file divided into three folders for original source data images for Ki67 images, mouse images, and immunofluorescence images. Subfolders are labeled with the corresponding Figure number in which the image appears. [file 41586_2024_7812_MOESM9_ESM.zip › Images/Mouse Images/Extended_Data_FIg_8_mouse_images/b/Methigh26_IACS_2.tif]
