# Supplementary material for: Mitochondrial complex I promotes kidney cancer metastasis
Source: Nature. Author manuscript; Available in PMC 2024 Sep 26. (PMC11424252; doi:10.1038/s41586-024-07812-3)

Supplementary Figure 1: Uncropped Western blots from Extended Data Fig. 8m and Extended Data Fig. 9l. FLAG and β-actin were probed on the same membrane.

FLAG:

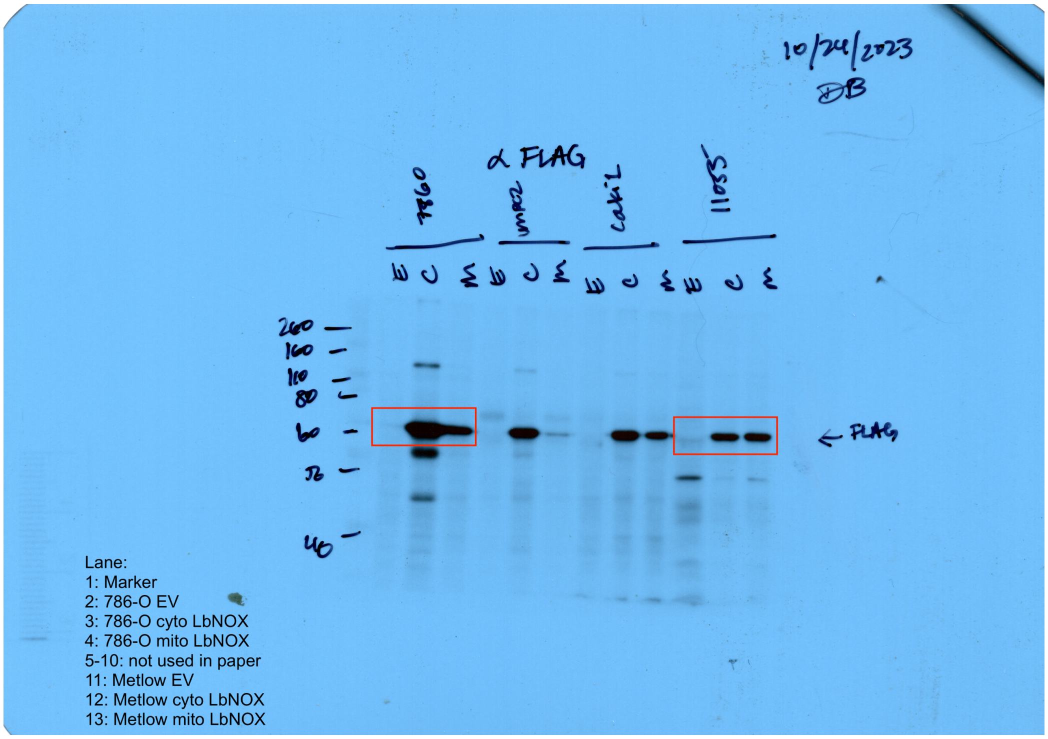

β-actin:

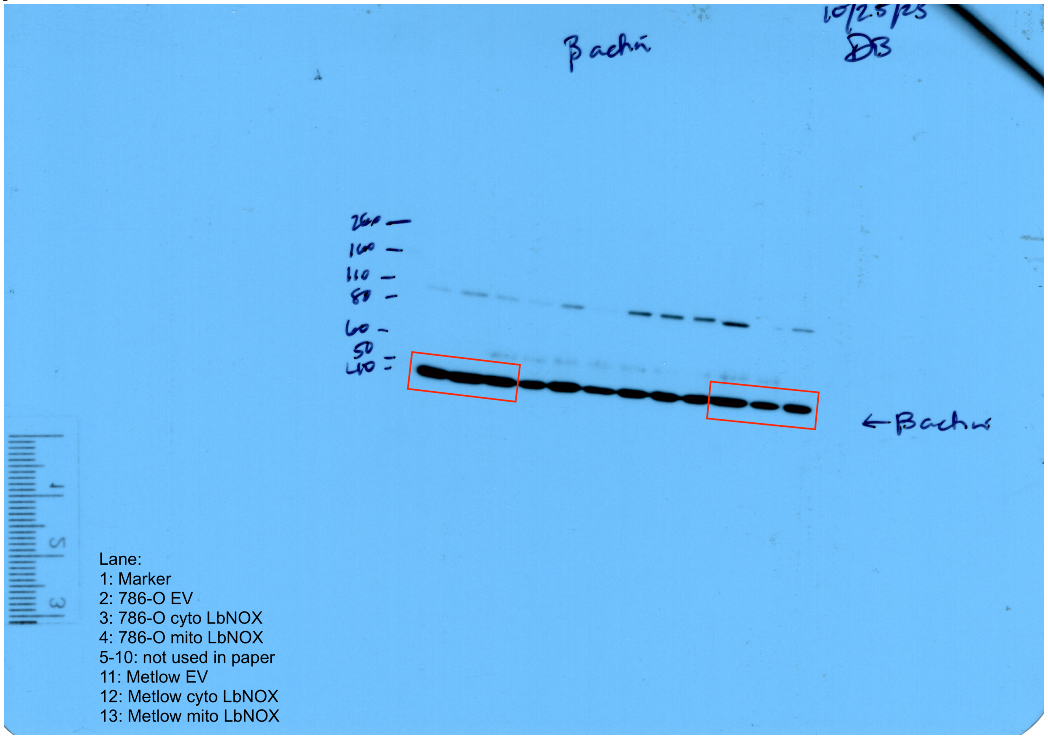

Supplementary Figure 2: FACS plots exemplifying the gating strategy used to sort dsRed positive cells.

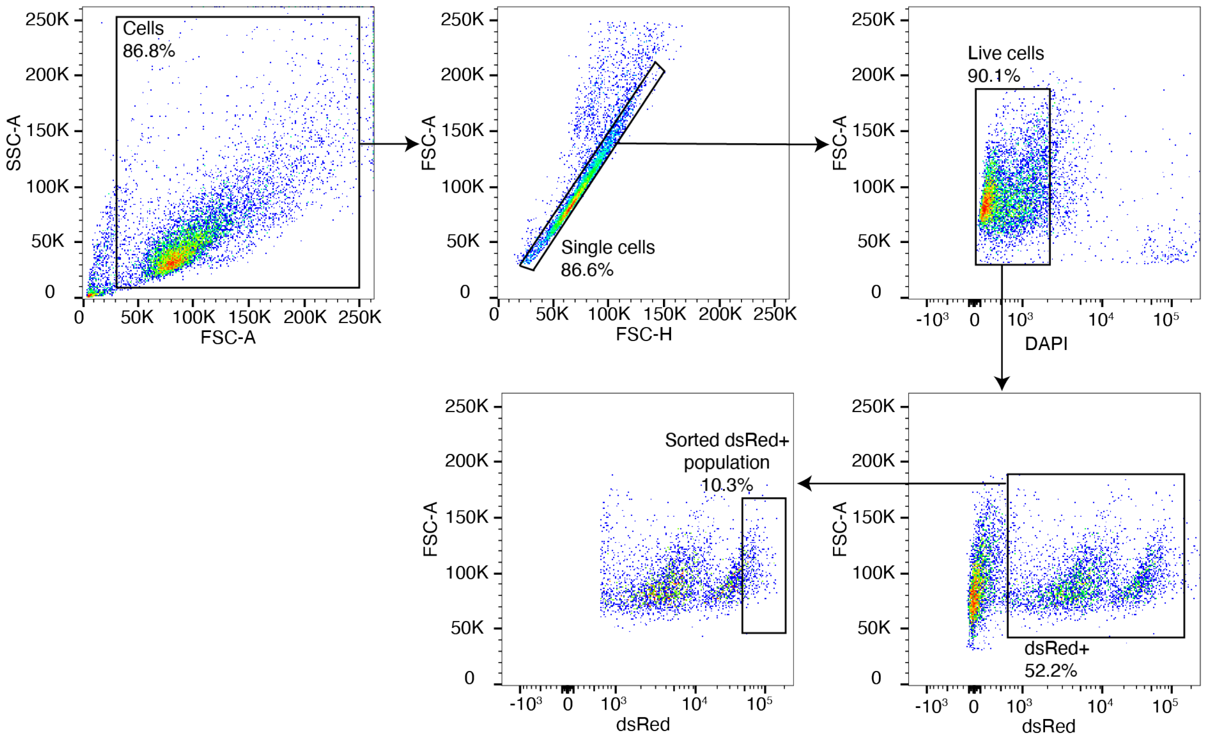

Supplement: Supplementary file 1 [file NIHMS2016485-supplement-Supplementary_Figures.pdf]
