# Supplementary material for: Mitochondrial complex I promotes kidney cancer metastasis
Source: Nature. Author manuscript; Available in PMC 2024 Sep 26. (PMC11424252; doi:10.1038/s41586-024-07812-3)
Supplement: Supplementary file 3 [file NIHMS2016485-supplement-Supplementary_Information_Guide.doc]

Supplementary Information Guide

Bezwada et al, 2024

Supplementary Figure 1: Uncropped Western blots from Extended Data Fig. 8m and Extended Data Fig. 9l. Supplementary Figure 1 and 2 are provided as a single PDF.

Supplementary Figure 2: FACS plots exemplifying the gating strategy used to sort dsRed positive cells. Supplementary Figure 1 and 2 are provided as a single PDF.

Supplementary Data Table 1: Clinical information of ^{13}C-nutrient-infused patients. Provided as an .xlsx file.

Supplementary Data Table 2: RNA sequencing for indicated patient tissues. Gene expression is shown as $\log_2$(counts per million (CPM)). Genes with 0 values for more than 75% of samples were removed. Provided as an .xlsx file.

Supplementary Data Table 3: Metabolomics of patient tissues Provided as an .xlsx file.

Supplementary Data Table 4: Isotopologue data from [U-^{13}C]glucose-infused patients. Provided as an .xlsx file.

- 4.1: Isotopologue data from [U-^{13}C]glucose-infused patient tissues
- 4.2: Isotopologue data from [U-^{13}C]glucose-infused patient plasma
- 4.3: Plasma glucose m+6 at time of tissue resection

Supplementary Data Table 5: Isotopologue data from [1,2-^{13}C]acetate-infused patient tissues. Provided as an .xlsx file.

Supplementary Data Table 6: Isotopologue data from [U-^{13}C]glutamine-infused patients. Provided as an .xlsx file.

- 6.1: Isotopologue data from [U-^{13}C]glutamine-infused patient tissues
- 6.2: Isotopologue data from [U-^{13}C]glutamine-infused patient plasma

Source data for all images: .zip file divided into three folders for original source data images for Ki67 images, mouse images, and immunofluorescence images. Subfolders are labeled with the corresponding Figure number in which the image appears.
